# A Case-Based Critical Care Curriculum for Internal Medicine Residents Addressing Social Determinants of Health

**DOI:** 10.15766/mep_2374-8265.11128

**Published:** 2021-03-18

**Authors:** Deepa Ramadurai, Ellen E. Sarcone, Mark T. Kearns, Anna Neumeier

**Affiliations:** 1 Fellow of Pulmonary, Allergy, and Critical Care Medicine, University of Pennsylvania; 2 Assistant Professor, Division of Hospital Medicine, Denver Health and Hospital Authority; 3 Assistant Professor, Division of Pulmonary Sciences and Critical Care Medicine, Denver Health and Hospital Authority; Assistant Professor, Division of Pulmonary Sciences and Critical Care Medicine, University of Colorado Anschutz Medical Campus

**Keywords:** Social Determinants of Health, Social Risk, Interdisciplinary Medicine, Critical Care Medicine, Case-Based Learning, Diversity, Inclusion, Health Equity

## Abstract

**Introduction:**

Graduate medical education on social determinants of health (SDOH) is limited. Residents often directly care for vulnerable populations at safety-net hospitals, yet curricula thus far are based in the ambulatory setting.

**Methods:**

We developed a case-based curriculum integrating SDOH with critical care topics to standardize knowledge and improve skills and attitudes of internal medicine residents working with these patients. We conducted a needs assessment, identified systematic social risk domains, and modified a published curriculum to develop the content. Case-based discussions were conducted weekly in the medical intensive care unit, while knowledge, attitudes, and skills were assessed daily during multidisciplinary rounds. A 360-degree assessment was completed with pre- and postcurriculum surveys and self-reflection.

**Results:**

Eleven residents completed postcurriculum surveys. Both pre- and postcurriculum, residents reported confidence in identifying and describing how SDOH affect care. After the curriculum, residents could name more resources for patients experiencing health disparities due to substance abuse (pre: 47%, post: 73%) and financial constraints (pre: 50%, post:64%). This curriculum was recognized as the first training many residents received (pre: 31%, post: 91%) with formal feedback (pre: 16%, post: 64%).

**Discussion:**

Implementing a curriculum of social risk assessment in critically ill patients was difficult due to competition with clinical care. Participating residents said they “loved the open dialogue” to reflect on their experiences; this became an avenue to “debrief on specific patient encounters and [how] SDOH brought [patients] to the ICU.” Future directions include qualitative analysis of reflections and assessment of curricular impact on trainee resiliency.

## Educational Objectives

By the end of this activity, learners will be able to:

1. Identify the social determinants of health in care during critical illness.
2. Describe methods to screen patients for social determinants of health.
3. Gain confidence in discussing unique barriers to health care with patients.
4. Reflect on personal experiences with patients whose access to health care is limited due to social determinants.
5. Collaborate with a team of health care providers to determine appropriate resources for patients whose health is affected by social determinants.

## Introduction

Many national professional societies, including the Institute of Medicine,^1,2^ the American Academy of Family Physicians,^3^ the American Academy of Pediatrics,^4^ and the American College of Obstetricians and Gynecologists,^5^ recommend physicians screen their patients for social determinants of health (SDOH). Despite the importance of screening for SDOH within a practice and these organizational mandates, education on health disparities and treatment of vulnerable patient populations is limited during graduate medical education (GME) and clinical training. The Accreditation Council for Graduate Medical Education (ACGME) milestones linked to core competencies for training programs do mention customizing care in the context of the patient and partnership with a variety of health care providers, in addition to understanding how common socioeconomic barriers impact patient care.^6^ Undergraduate medical education (UME) programs have implemented teaching on the importance of SDOH in the scope of patient care,^7,8^ as this has been recognized on a national level as critical to education of the next generation of physicians.^9,10^ Existing SDOH curricula in GME have mostly occupied the primary care setting in internal medicine,^11,12^ family medicine,^13^ and pediatric residency programs.^14–16^ Notably, pediatric ACGME core competencies include communication with the interdisciplinary team to coordinate patient care and advocacy for quality of patient care,^17^ whereas family medicine ACGME core competencies specifically emphasize the importance of innovation and advocacy for populations and communities that experience health equities under systems-based practice.^18^ Studies in these programs have therefore largely focused on learner attitudes and competencies in the management of patients with significant social risk. However, there is a general lack of clarity in training programs across the country on what training should encompass, what the competencies should be for SDOH curricula, and what are resources available to structure curricula upon.^19^

Residents at the front line of care for vulnerable populations in safety-net hospitals are in an optimal position to initiate a screening tool for SDOH in order to facilitate interdisciplinary management during a hospital stay. Existing curricula at safety-net hospitals that have introduced specific training on SDOH focus on the ambulatory care setting using case-based discussion to improve familiarity with community resources.^12,16^ A structural competency curriculum in the San Francisco Bay area for interprofessional teams demonstrates the power of equipping learners to acknowledge and address SDOH in patient health.^20^ Medical schools have widely implemented curricula focused on SDOH,^8,21,22^ but the GME experience is much more strained for time, with limited space for didactic teaching and different learner needs more focused on understanding application and implementation within clinical care, and therefore, it is difficult to apply these curricula. No such curriculum exists in critical care, where the rapid pace of clinical changes, emphasis on understanding core pathophysiologic concepts of critical care medicine, and intensity of moral and ethical challenges at the end of life may overshadow the importance of a thorough social history for a new trainee.

Recent publications have highlighted the need to address SDOH and the importance of physician screening practices.^10,23,24^ Led predominantly by the American Heart Association, subspecialty societies also endorse the importance of recognizing and understanding SDOH in the context of acute and chronic illness.^25^ The American Thoracic Society and National Heart, Lung, and Blood Institute's workshop to address respiratory equality in the United States in 2015 identified screening and education for trainees as an integral part in slowing or halting the progression of disease risk to disease manifestation and poor outcomes.^26^ Beyond this, there is evidence to suggest that assessment of social risk in critically ill patients has significant benefit, specifically through the use of trauma-informed care screening to improve psychobehavioral outcomes.^27^ Additionally, it is known that mortality is disparate in medical intensive care unit (MICU) patients based on race (disparately high in Blacks and Hispanics amongst other racial minorities)^28^ and area of residence (more in underserved areas).^29^ End-of-life care also looks different for minority populations, and this is not fully explained by sociocultural preferences.^30^

We therefore sought to develop structured SDOH education in the MICU, interweaving it with essential critical care topics. We designed a multifaceted curriculum with the goal of improving residents' knowledge, attitudes, and skills in order to perform social risk assessment, identify patient needs, and enhance their contribution to the multidisciplinary care within a care management team. Adult learning strategies of problem-based learning, self-reflection, and performance feedback were pursued with hopes of improving clinical learning and therefore clinical outcomes.^31^

## Methods

### Needs Assessment

We performed a targeted needs assessment that surveyed internal medicine residents at the University of Colorado's Internal Medicine Residency Program, inquiring as to residents' confidence and ability to manage patients at high risk for disparate outcomes based on social circumstances (Appendix A). Thirty-eight residents responded to our needs assessment survey (21% response rate, 38 out of 180) and were evenly distributed amongst postgraduate years 1, 2, and 3: 29% (11 out of 38), 34% (13 out of 38), and 37% (14 out of 38), respectively. The majority reported they strongly agreed that understanding health disparities was important in order to provide care for ICU patients (71%, 27 out of 38). Most residents strongly agreed (47%, 18 out of 38) or somewhat agreed (50%, 19 out of 38) they could define SDOH, yet only somewhat agreed (70%, 26 out of 38) they could identify the health care disparities of patients. Even fewer somewhat agreed (55%, 21 out of 38) that they could discuss unique barriers to health care with their patients. There was a range of opinions on comfort with utilizing resources to care for patients with health disparities: Five strongly agreed (13%), 19 somewhat agreed (50%), eight neither agreed nor disagreed (21%), four somewhat disagreed (11%), and two strongly disagreed (5%). The majority of respondents agreed that they could define SDOH, but only about half of respondents knew how to utilize community resources to care for patients with health care disparities. Although residents believed they could define SDOH and agreed upon the importance of understanding food insecurity, health burdens amongst patients with homelessness and refugee population, and types of insurance subsidy programs available to patients, they assessed their current training in these domains to be inadequate.

### Curriculum Development

We developed curricular goals and objectives guided by the Association of American Medical Colleges (AAMC) quality improvement and patient safety (QIPS) competencies in health equity.^32^ Health equity in QIPS focuses on providing efficacious care to patient populations, focusing on utilizing patient data on SDOH, identifying physician factors that may contribute to disparities, and engaging patients and families with regard to reducing SDOH. The educational goals of the curriculum were to improve resident understanding and interpretation of SDOH in the context of critical illness and to develop the ability to perform comprehensive social risk assessment. We identified competencies 2, 5, and 10 in the AAMC QIPS report as the basis for our learning objectives. These competencies encompassed the following: (1) collection of an SDOH-focused history with description of their effect on quality of patient care, (2) knowledge and identification of implicit and explicit biases, and (3) recognition of systems factors and influence of systems facts on health care inequities of the local patient population. To achieve these educational goals and objectives, we used the following educational strategies: problem-based learning, self-reflection, and performance feedback as part of the supervised clinical experience. A detailed facilitator guide is provided in Appendix B.

Using the needs assessment, we identified SDOH topics to embed in the case-based critical care curriculum. This content was further refined based on the environmental needs of our patient population and the Accountable Health Communities Health-Related Social Needs Screening Tool (AHC HRSN), developed by the Centers for Medicare & Medicaid Services (CMS) to promote a systematic method to identify and address health-related social needs (Appendix C).^33^ This tool identified five domains for which explicit screening was needed regarding social risk: housing instability, food insecurity, transportation problems, utility help needs, and interpersonal safety. We chose this particular tool because it went beyond the typical social history (discussing alcohol use, illicit drug use, living situation, and general support system), probing into topics directly impacting social risk that could be addressed with tangible solutions from community resources. We identified 16 critical care topics; 10 cases were adapted from a prior *MedEdPORTAL* curriculum with author permission.^34^ We expanded these cases with an additional six topics based on common pathologies encountered in the MICU population in our safety-net hospital (Appendix D). The additional critical care case topics were designated by MICU faculty as core topics important for management of patients in our ICU.

### Curriculum Implementation

**Figure 1.**
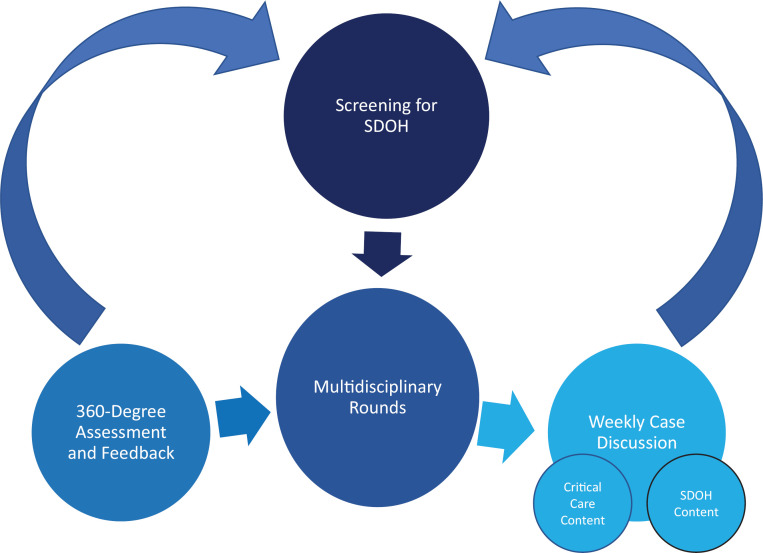
Curriculum map. The first session occurs during orientation and reviews how to screen for social determinants of health (SDOH). Learners then apply the screening in patient interactions and discuss social risks during multidisciplinary rounds. They receive feedback from the care management team and from their attendings, as well as individually reflecting on their knowledge before and after the curriculum, within the 360-degree assessment and feedback. The SDOH concepts are reiterated and applied to critical care cases during the weekly case discussions.

The chief resident (Deepa Ramadurai) and the unit social worker led the weekly curricular sessions, at times accompanied by the MICU fellow or attending. Seminars were delivered in the fall of the 2019–2020 academic year. On the first day of the rotation (Monday) during the typical afternoon MICU orientation, the chief resident reviewed the rationale and objectives of the curriculum and set expectations for learner participation, including a prompt for residents to start thinking of cases on their teams that would be helpful to review given the challenging topics related to patients' SDOH. The orientation to the curriculum also included a basic overview of what screening for social risk entailed and the core areas identified in the AHC HRSN. The chief resident attended the multidisciplinary rounds meeting (held each weekday morning in the MICU between 8:00 am and 8:15 am) once or twice a week to identify potential cases. Delivery of the curriculum occurred in the conference room in the MICU on Friday afternoons at 2:00 pm. The room included a central table around which the learners were seated as well as a computer connected to a projector that could display the patient's medical chart. Additionally, there was a whiteboard in the room. Materials for the SDOH topics were often printed in advance and passed around to the learners (e.g., a Medicaid application during the insurance subsidy discussion). A curriculum map in Figure 1 outlines how implementation was carried out from the orientation session to the weekly discussions and 360-degree assessment.

Training for the facilitator included review of the facilitator guide (Appendix B) as well as the prompt questions within the SDOH topics (Appendix C). Facilitators had to review the SDOH topic to be presented and often discuss it with the unit social worker, nutritionist, or another content expert (at our hospital, this was usually faculty members who had research interest in different SDOH topics) prior to the topic discussion to ensure high-yield points were brought up. The facilitator carried copies of the case templates and facilitator guide for each session. Depending on the comfort level of the faculty member or MICU fellow with the SDOH material, the chief resident may or may not have been present for each session to provide the SDOH content delivery. The content facilitator also had to be familiar with the open-discussion questions listed in the facilitator guide and typically introduced these during the discussion of each topic. The faculty and fellows were provided with the facilitator and topic guides.

To initiate the discussion, residents were asked to briefly present either a patient case prompted by the chief resident (identified to have significant social risk factors from multidisciplinary rounds) or a trainee-identified case where SDOH were a major factor in the patient's care (Appendix E). The critical care topic of that patient was discussed first, utilizing the learning objectives and general teaching points from the outlined critical care cases. After completion of the clinical care and management discussion, the chief resident prompted discussion of SDOH within the case. Discussion typically started with the question of how a provider should screen the patient and/or family member for social risk (using the AHC HRSN). We then asked residents which area of social risk they wanted to focus on within the case.

**Figure 2.**
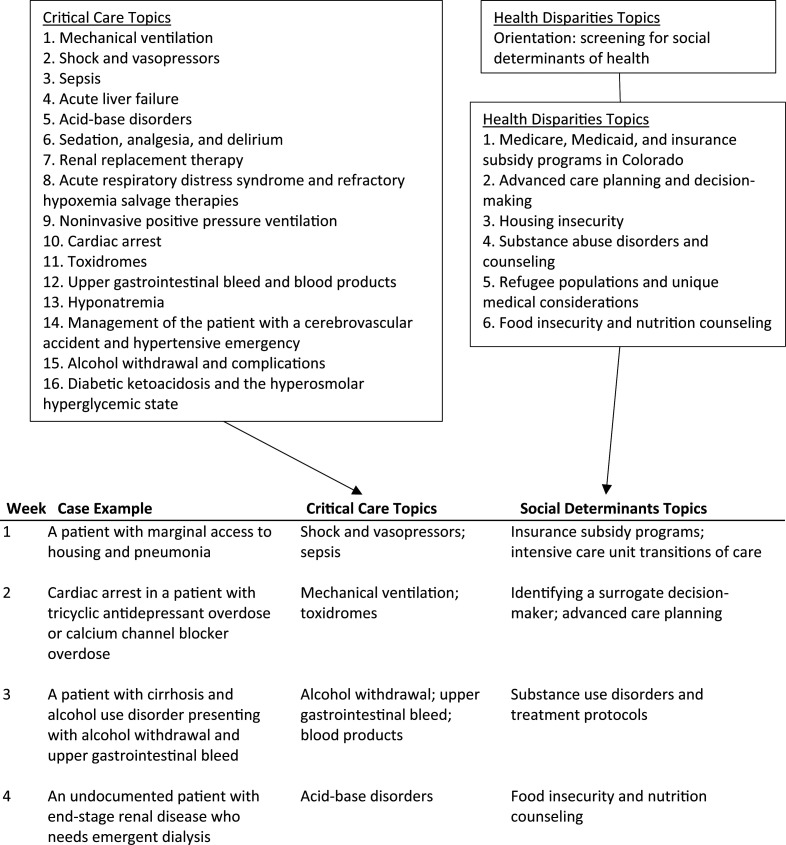
Sample schedule based on a 4-week medical intensive care unit rotation.

The first week typically was a discussion of Medicare, Medicaid, and insurance subsidy programs or advanced care planning in the critically ill patient. Subsequent topics were left to the discretion and desire of the learners. Figure 2 details a sample schedule with critical care topics and SDOH topics for a 4-week MICU rotation. The facilitator asked prompting questions and utilized the suggested learning activities associated with the SDOH topic. If a factual question came up from the group, it was fielded by either the social worker or the facilitator (most commonly the chief resident). After considering how that social risk factor was affecting the patient's case, we turned to a discussion of resources to address this social determinant, whether before, during, or after the patient's ICU stay. During this discussion, the facilitator elicited fears and concerns from the residents regarding how, when, and why each social determinant was being addressed. Residents were asked to recall any prior experiences with similar patients as well as how to learn from those scenarios moving forward. Open discussion ensued, with prompting questions from the facilitator and social worker. At the end of discussion, the resident MDR who presented the case summarized what had been learned from it, and any other closing remarks were elicited from the group.

Each session lasted approximately 30 minutes. Residents then had the opportunity to apply information learned during the daily multidisciplinary MICU rounds.

### 360-Degree Assessment

Learners completed pre- and postcurriculum surveys to assess their changes in attitudes and self-perceived knowledge and skills (Appendix F). Additionally, learners received direct observation and feedback on the incorporation of their social risk assessments during multidisciplinary rounds from the unit social worker, using a templated feedback form (Appendix G). For systems that do not have a social worker or case manager present, this portion of the assessment could be completed by the bedside nurse or another member of the interdisciplinary team (e.g., specialist, physical therapist, nutritionist). The critical care fellows and attending physicians were also encouraged to provide structured verbal or written feedback to the residents (Appendix H). Attendings could include this feedback in the Internal Medicine Residency Program's structured MedHub feedback for the rotation.

## Results

Between July and November 2019, 32 residents participated in the curriculum. Twenty-seven of these residents were from internal medicine, two from family medicine, and three from emergency medicine. Thirty-two precurriculum surveys and 11 postcurriculum surveys were completed. Approximately 50% of the cases discussed were brought forth by the residents, who identified SDOH as a major factor in ongoing care of the patient. The other 50% of the cases were triggered by the chief resident's identification during multidisciplinary rounds. Given that the critical care topic was taken from the patient case initially identified for social risk, the provided case was often modified slightly to more accurately reflect the ICU patient rather than use of the template.

Precurriculum, most residents felt comfortable identifying and describing how SDOH affect quality of care for patients experiencing disparities in the MICU (27 out of 32, 84%), and this was improved further postcurriculum (11 out of 11, 100%). Residents felt very comfortable throughout in recognizing more than one social domain that could contribute to social risk (pre: 20 out of 21, 96%; post: 11 out of 11, 100%) and recognized that providers should screen for social risk (pre: 91% vs. post: 100%). Despite this, the residents felt relatively uncomfortable in accessing resources for patients with health disparities. The most striking improvement between pre- and postcurriculum surveys was in the ability to name specific resources for patients with health disparities related to substance abuse (pre: 47% vs. post: 73%) and financial constraints (pre: 50% vs. post: 64%).

During the open discussion, residents were unfamiliar with specific domains. For example, most residents could not identify food insecurity, transportation needs, and utility needs as pertinent social domains in medical care. In conversation during sessions, while detailing the components of the AHC HRSN, trainees were surprised to hear that a number of their patients did not have basic amenities, including heat and electricity at home on a reliable basis.

All residents who completed the postcurriculum survey reported comfort in their ability to perform social risk assessment (11 out of 11). Residents could more readily name specific resources that could be valuable for patients with substance use disorders and financial constraints after the curriculum. Residents recognized this curriculum as their first formal training with dedicated feedback on social risk assessment. These results are presented in the Table.

**Table.**
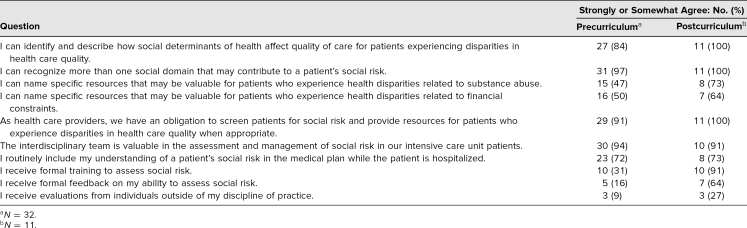
Resident Responses to Pre- and Postcurriculum Surveys

Open-ended responses on the postcurriculum survey included the following:

• “I loved the open dialogue.”
• “Discussion on insurance and how that effects patient care in the hospital.”
• “Information about resources available to our patients.”
• “Debriefs on specific patient encounters and discussing SDOH and how it brought [patients] to the ICU.”

At the open-format weekly discussion, residents additionally noted it was difficult to integrate social determinants screening when dealing with active critical care medicine. The often-rapid transfer of patients from emergency room to the ICU and then to the floor (patients were frequently transferred out of the ICU within 12 hours of admission) made continuity and relationship-building challenging. Residents sought tips or techniques to incorporate the social risk screening in a sensitive manner while building rapport.

Residents appreciated receiving this learning in the safety-net hospital setting. The applicability of the content in real time to their patients was satisfying. Residents sought out the unit social worker or the authors to discuss the specifics of the social aspects of a patient's care with more frequency since discussion of these aspects was not limited to just multidisciplinary rounds.

The social worker provided direct feedback to 10 residents over the course of the curriculum implementation, utilizing the brief template created. Attending feedback was performed through our evaluation templates and was included individually through our internal medicine program computer-generated evaluations in MedHub. The comments from the social worker were constructive and included key words or phrases that could have been utilized in each case and a general assessment of learner engagement in the multidisciplinary process. Based on discussion with the social worker and faculty, completion of feedback on their end took roughly 10 minutes for each trainee. Assessment was easy to incorporate as all individuals were present for daily multidisciplinary rounds, as previously mentioned.

## Discussion

Health disparities must be addressed in critically ill patients, per the American Thoracic Society,^26^ yet curricula to guide education in this domain remain limited. Our resource demonstrates an innovative platform to embed SDOH education in a critical care curriculum and therefore empower trainees within the multidisciplinary team to ally with the unit care management team. Our study drew from national resources to create a standardized and reproducible curriculum that can be adapted to individual communities. Prior studies that incorporated SDOH teaching at the UME and GME levels referenced community working groups^35^ or physician experts^12^ who had been working with the patient populations for years. We utilized a combination of resources from national societies, including the AHC HRSN and the AAMC QIPS competencies, to develop a structured approach to the education of trainees in SDOH. The strengths of this curriculum include the innovative design and multidisciplinary approach to feedback and evaluation of resident assessment of social risk. The process of screening for social risk is inherent in the social history, and additional questioning can be readily implemented as per the CMS guidance with the AHC HRSN. By interweaving the curriculum into multiple components of the clinical learning environment, the importance of the content is highlighted, with equal emphasis placed on SDOH topics and clinical medicine topics.

Our experience revealed valuable lessons learned. Creation of sustainable logistics with this novel curriculum was crucial to its success. This included incorporation of content into the existing rotation structure: the multidisciplinary rounds and the dedicated teaching time. Involving the multidisciplinary team allowed for more detailed conversation and more valuable feedback for the trainees who participated. The creation of a formal reflective space to discuss social risk during a safety-net hospital rotation also enhanced the perceived immediate applicability of some of these concepts. Residents reported fear and uncertainty over asking questions that they did not have answers to, therefore often omitting these questions altogether. They also reported having limited time as being a barrier to asking these questions and identified prioritizing the performance of a full medical history as needed for the acute condition. With reflective space, we navigated fears for time constraints with the residents and encouraged the dialogue that promotes the provider-patient relationship rather than feeding the sense of inadequacy in not knowing the answers. Through this process, it became clear to the residents that asking the questions to promote a better understanding of the patient, in order to adapt their disease management to something practical and patient centered, was ultimately more impactful.

Two responses to the resident survey had lower percentages of agreement postcurriculum. The first involved a statement about the value of the interdisciplinary team in the assessment and management of social risk in our ICU patients (pre: 94%, post: 91%). The small numbers of postcurriculum responses may have contributed to these differences. However, it is possible that residents did not initially understand the purpose and resources of the multidisciplinary team and that learning about their involvement in patient care through this curriculum altered the residents' views of the team's strengths. Beyond assessment and management of the patient, the multidisciplinary team plays a role in information gathering and sharing and is in a unique position to educate both patients and providers about the social complexity of these patients. Perhaps these qualities became more prominent as a result of the curriculum or perhaps residents became unsure of the specific responsibilities of the team, given that they were now involved in social risk assessment as well. The second survey response demonstrating a decline postcurriculum was whether the residents routinely included their understanding of a patient's social risk in the medical plan while the patient was hospitalized (pre: 72%, post: 60%). It is possible that residents realized they did not routinely include understanding of social risk in a patient's medical plan as a result of learning about the definitions of social risk through this curriculum. The adaptation and inclusion of social risk assessment in routine practice likely take much longer than the 4 weeks over which our assessment was performed. Residents previously may have thought they were addressing social risk but, after realizing the depth and breadth of these domains, understood that this was not routine in their assessment and plan.

There are several limitations to our curriculum. The evaluation of this program was limited by small numbers of postcurriculum survey respondents and therefore may have reflected the validity of the trend of improvement in self-reported skills and attitudes. It is possible that residents who took the postcurriculum survey were more invested and interested in understanding SDOH and therefore more likely to respond favorably to the concepts presented and to value this education. Additionally, although the target audience of our needs assessment was internal medicine residents, we ultimately delivered the curriculum to trainees from internal medicine, family medicine, and emergency medicine. Given their work roles, these groups may have had different needs that we did not identify. However, the curricular topic focus within the context of critical care provides a framework that can be generalized across specialties rotating within the critical care environment. However, this highlights that SDOH content is guided by the patient populations served, and therefore, the topics we developed may need some modification for other programs to implement the curriculum. The AHC HRSN and the AAMC QIPS competencies, the framework upon this curriculum is based, provide a backbone for topics to utilize. Topics can be further refined with assistance from faculty and the multidisciplinary team, which is crucial to delivering population-specific and appropriate content. Furthermore, although residents were evaluated by objective performance measures as a part of their rotation evaluation, whether or not the curriculum resulted in objective performance improvement could not be determined due to the anonymous survey methodology we used. Creating a designated time and space (e.g., after the last session of the 4-week rotation) for the attending physician to provide dedicated feedback on the residents' application of SDOH in each patient case could have been more robust. Future curricular directions and revisions should incorporate both effective real-time evaluation strategies and longitudinal assessment to measure impact across the continuum of learning. Implementation of this curriculum requires facilitation from social work disciplines external to medicine, which, although being one of the curriculum's great strengths, could pose implementation challenges depending on the setting and access to social work expertise. However, as multidisciplinary teams are composed differently across learning environments, identifying specialty content lead for SDOH content would be applicable to many disciplines.

Future directions involve expanded implementation and more in-depth curricular evaluation. This curricular framework, pairing clinical content with SDOH content, can be applied across clinical settings. Therefore, next steps in implementation would be to expand delivery across other clinical learning environments. Further assessment of the curriculum using qualitative methods could better identify trainee professional development needs as well as measure impact. Discussing SDOH with residents in clinically rigorous environments such as the MICU can provide an outlet to refocus on competence and relatedness—two components of care identified as important for maintaining resilience in an age of depersonalized care.^36^ Notably, this was something that was realized in Neff and colleagues' study of teaching structural competency to interprofessional teams including physicians, trainees, and students.^20^ Their qualitative analysis revealed a renewal of connectedness to why group members joined their professions as well as a reminder of empathy and humility in interacting with patients. The open format of discussion could provide valuable insight on connecting SDOH training to trainee resiliency, which should be assessed. Furthermore, longitudinal assessment of learners throughout their training to measure the effectiveness of this curriculum is also needed. An additional area in which to measure curricular impact is obtaining feedback from patients and their families regarding perceived attitudes and behaviors of their providers. Little is known about the effect of SDOH screening from the patient and family member perspective. This could further inform whether implicit and explicit bias training in this setting may be important to improving the patient and caregiver experience. Finally, expanding the 360-degreee assessment by assessing the perceptions of ancillary services in our MICU (including physical therapy, occupational therapy, nutrition, glucose management, and palliative care) regarding residents' ability to appropriately identify social factors necessitating interdisciplinary care could strengthen the assessment further.

This curriculum presents a novel design for discussing SDOH in the setting of critically ill patients. It not only reemphasizes the importance of instilling a consistent and purposeful social risk assessment as a best practice for trainees but also provides an opportunity for open dialogue and discussion with patients that facilitates more meaningful patient-centered care.

## Appendices

Needs Assessment.docxFacilitator Guide.docxSDOH Topics Guide.docxCritical Care Cases.docxMDR Checklist.docxPre- and Postcurriculum Surveys.docxCare Team Checklist.docxAttending Checklist.docx
All appendices are peer reviewed as integral parts of the Original Publication.
