# Supplementary material for: A Case-Based Critical Care Curriculum for Internal Medicine Residents Addressing Social Determinants of Health
Source: MedEdPORTAL. 2021 Mar 18;17:11128. doi: 10.15766/mep_2374-8265.11128 (PMC8015637; doi:10.15766/mep_2374-8265.11128)
Supplement: Supplementary file 1 — Needs Assessment.docxFacilitator Guide.docxSDOH Topics Guide.docxCritical Care Cases.docxMDR Checklist.docxPre- and Postcurriculum Surveys.docxCare Team Checklist.docxAttending Checklist.docx [file mep_2374-8265.11128-s001.zip › A. Needs Assessment.docx]

1. Please indicate your year of training:

- PGY-1
- PGY-2
- PGY-3
- PGY-4

Please read each of the following statements and indicate to what extent you agree or disagree.

2. Understanding health disparities is important when it comes to providing care to patients in the intensive care unit.

**Strongly agree**

**Somewhat agree**

**Neither agree nor disagree**

**Somewhat disagree**

**Strongly disagree**

3. I can define the concept of “social determinants of health”.

**Strongly agree**

**Somewhat agree**

**Neither agree nor disagree**

**Somewhat disagree**

**Strongly disagree**

4. I feel comfortable discussing with my patients what unique barriers to health care exist for them.

**Strongly agree**

**Somewhat agree**

**Neither agree nor disagree**

**Somewhat disagree**

**Strongly disagree**

5. I can identify the healthcare disparities of patients in the intensive care unit

**Strongly agree**

**Somewhat agree**

**Neither agree nor disagree**

**Somewhat disagree**

**Strongly disagree**

6. I know how to utilize resources to care for patients with health disparities.

**Strongly agree**

**Somewhat agree**

**Neither agree nor disagree**

**Somewhat disagree**

**Strongly disagree**

7. It is important to understand homelessness in the Denver population in order to provide and facilitate care for our patients at Denver Health.

**Strongly agree**

**Somewhat agree**

**Neither agree nor disagree**

**Somewhat disagree**

**Strongly disagree**

8. It is important to inquire about substance use disorders and how to counsel patients appropriately in order to facilitate care for our patients at Denver Health.

**Strongly agree**

**Somewhat agree**

**Neither agree nor disagree**

**Somewhat disagree**

**Strongly disagree**

9. It is important to understand the unique health issues of refugee populations in the Denver metro area in order to provide and facilitate care for our patients at Denver Health.

**Strongly agree**

**Somewhat agree**

**Neither agree nor disagree**

**Somewhat disagree**

**Strongly disagree**

10. It is important to understand the practice of emergent hemodialysis for those without access to regular hemodialysis in order to provide and facilitate care for our patients at Denver Health.

**Strongly agree**

**Somewhat agree**

**Neither agree nor disagree**

**Somewhat disagree**

**Strongly disagree**

11. It is important to understand food security and nutrition counseling in order to provide and facilitate care for our patients at Denver Health.

**Strongly agree**

**Somewhat agree**

**Neither agree nor disagree**

**Somewhat disagree**

**Strongly disagree**

12. It is important to understand differences between insurance subsidy programs in order to provide and facilitate care for our patients at Denver Health.

**Strongly agree**

**Somewhat agree**

**Neither agree nor disagree**

**Somewhat disagree**

**Strongly disagree**

13. I receive adequate education on homelessness and its effects on health care in the Denver.

**Strongly agree**

**Somewhat agree**

**Neither agree nor disagree**

**Somewhat disagree**

**Strongly disagree**

14. I receive adequate education on substance abuse disorders and counseling the patients we care for at Denver Health.

**Strongly agree**

**Somewhat agree**

**Neither agree nor disagree**

**Somewhat disagree**

**Strongly disagree**

15. I receive adequate education on refugee populations of the Denver metro area in order to provide care for our patients at Denver Health.

**Strongly agree**

**Somewhat agree**

**Neither agree nor disagree**

**Somewhat disagree**

**Strongly disagree**

16. I receive adequate education on emergent hemodialysis for those without access to regular hemodialysis in Denver in order to provide care for our patients at Denver Health.

**Strongly agree**

**Somewhat agree**

**Neither agree nor disagree**

**Somewhat disagree**

**Strongly disagree**

17. I receive adequate education on food security in Denver in order to provide care for our patients at Denver Health.

**Strongly agree**

**Somewhat agree**

**Neither agree nor disagree**

**Somewhat disagree**

**Strongly disagree**

18. I receive adequate education on nutrition counseling in order to provide care for our patients at Denver Health.

**Strongly agree**

**Somewhat agree**

**Neither agree nor disagree**

**Somewhat disagree**

**Strongly disagree**

19. I receive adequate education on the differences between Medicaid, Medicare, CICP, and DFAP.

**Strongly agree**

**Somewhat agree**

**Neither agree nor disagree**

**Somewhat disagree**

**Strongly disagree**

20. I want to learn more about the following social topics with relation to how they affect my patients’ health care:

- Homelessness in the Denver population
- Inquiring about substance abuse disorders and counseling patients appropriately
- Refugee populations of the Denver metro area
- Emergent hemodialysis for those without access to regular hemodialysis
- Food security in Denver
- Nutrition counseling
- Differences between Medicaid, CICP, and DFAP

21. Please indicate any additional topics you would like to learn about with regards to social determinants of health: _________________________________________________________
