# Supplementary material for: A Case-Based Critical Care Curriculum for Internal Medicine Residents Addressing Social Determinants of Health
Source: MedEdPORTAL. 2021 Mar 18;17:11128. doi: 10.15766/mep_2374-8265.11128 (PMC8015637; doi:10.15766/mep_2374-8265.11128)
Supplement: Supplementary file 1 — Needs Assessment.docxFacilitator Guide.docxSDOH Topics Guide.docxCritical Care Cases.docxMDR Checklist.docxPre- and Postcurriculum Surveys.docxCare Team Checklist.docxAttending Checklist.docx [file mep_2374-8265.11128-s001.zip › B. Facilitator Guide.docx]

**Medical Intensive Care Unit (MICU) Health Disparities Curriculum: Facilitator Guide**

Educational Objectives

By the end of this activity, learners will be able to:

1. Identify the social determinants of health in care during critical illness.

2. Describe methods to screen patients for social determinants of health.

3. Gain confidence in discussing unique barriers to health care with patients.

4. Reflect on personal experiences with patients whose access to health care is limited due to social determinants.

5. Collaborate with a team of health care providers to determine appropriate resources for patients whose health is affected by social determinants.

Curriculum Overview

There are three parties necessary to complete an afternoon curriculum session:

1. The facilitator of the curriculum (either the chief resident, MICU fellow, or faculty member or a combination of these individuals). This individual should be well-versed in the critical care content and objectives for one of the core topic areas listed below. The facilitator may also have additional knowledge on the social determinants of health (SDOH) topics to be discussed.
2. The MICU social worker, who will provide background information and knowledge on the social determinants of health (SDOH) topics as listed below.
3. The resident trainees).

Curriculum Schedule (an example):

The following is a calendar outline of the schedule of curriculum implementation for a 4-week ICU rotation where curricular activities occur on weekdays only. Every afternoon has reserved time for didactic/group teaching with one session per week dedicated towards paring a critical care topic with a SDOH topic. Orientation occurs on the first Monday of the rotation in the afternoon.

| Monday | Tuesday | Wednesday | Thursday | Friday |
| --- | --- | --- | --- | --- |
| **8-8:15am** MDR*  **2pm** Resident Orientation  Curriculum Intro  - Pre-Survey Completion  - Screening for SDOH Session | **8-8:15am** MDR  2pm Teaching | **8-8:15am** MDR  2pm Teaching | **8-8:15am** MDR  2pm Teaching | **8-8:15am** MDR  **2pm** Critical Care Case and SDOH Topic |
| **8-8:15am** MDR  2pm Teaching | **8-8:15am** MDR  2pm Teaching | **8-8:15am** MDR  2pm Teaching | **8-8:15am** MDR  2pm Teaching | **8-8:15am** MDR  **2pm** Critical Care Case and SDOH Topic |
| **8-8:15am** MDR  2pm Teaching | **8-8:15am** MDR  2pm Teaching | **8-8:15am** MDR  2pm Teaching | **8-8:15am** MDR  2pm Teaching | **8-8:15am** MDR  **2pm** Critical Care Case and SDOH Topic |
| **8-8:15am** MDR  2pm Teaching | **8-8:15am** MDR  2pm Teaching | **8-8:15am** MDR  2pm Teaching | **8-8:15am** MDR  2pm Teaching | **8-8:15am** MDR  **2pm** Critical Care Case and SDOH Topic |

*Multidisciplinary rounds: Residents, faculty, care management team (social worker, case manager), physical and occupational therapists, nutritionists, and respiratory therapists gather to briefly review each case in the ICU and discuss disposition planning and major changes for the day (e.g. transfer to the floor, engage hospice, etc.)

**Part 1: Curriculum Orientation and Introductory Session – Screening for Social Determinants of Health**

The goals of this session are to introduce the curricular expectations and how to screen for social determinants of health.

Activities:

1. Learners complete the pre-curriculum survey

2. Learners review and apply SDOH screening questionnaire (details in Screening for Social Determinants of Health session)

**Part 2: Discussion and Management of Identified Social Needs During Multidisciplinary Rounds**

Multidisciplinary rounds occur every morning, where residents present the patient’s identified medical needs, disposition needs ,and review social needs that have been identified through their screens.

**Part 3: Case Selection and Identification of the SDOH and Critical Care Topic Sessions**

Guided by cases of patients currently admitted, the facilitators should select a case that highlights one of the social determinates of health topics and then pair that topic with a relevant critical care topic.

The facilitator should attend 1-2 multidisciplinary rounds (MDR) sessions over the course of the week to identify potential cases that would be pertinent to discuss in the event that the residents are not able to initially identify these cases. Cases will typically be identified by a high-yield SDOH topic (based on the predetermined topics listed above) but can also be identified by an interesting critical care teaching case (based on the core topics listed above), to which a social determinants topic can be applied. Examples of general pairings with specific cases are outlined below.

Sample Schedule Based on a 4-Week Medical Intensive Care Unit Rotation

Health Disparities Topics

Orientation: Screening for Social Determinants of Health

Critical Care Topics

1. Mechanical Ventilation

2. Shock and Vasopressors

3. Sepsis

4. Acute Liver Failure

5. Acid-Base Disorders

6. Sedation, Analgesia, and Delirium

7. Renal Replacement Therapy

8. Acute Respiratory Distress Syndrome and Refractory Hypoxemia Salvage Therapies

9. Non-Invasive Positive Pressure Ventilation

10. Cardiac Arrest

11. Toxidromes

12. Upper Gastrointestinal Bleed and Blood Products

13. Hyponatremia

14. Management of the Patient with a Cerebrovascular Accident and Hypertensive Emergency

15. Alcohol Withdrawal and Complications

16. Diabetic Ketoacidosis and the Hyperosmolar Hyperglycemic State

Health Disparities Topics

1. Medicare, Medicaid, and insurance subsidy programs in Colorado

2. Advanced care planning and decision-making

3. Housing insecurity

4. Substance abuse disorders and counseling

5. Refugee populations and unique medical considerations

6. Food insecurity and nutrition counseling

**Week Case Example Critical Care Topics Social Determinants Topics**

| 1 | A patient with marginal access to housing and Pneumonia | Shock and vasopressors; Sepsis | Insurance subsidy programs; ICU transitions of care |
| --- | --- | --- | --- |
| 2 | Cardiac arrest in a patient with tricyclic antidepressant overdose or calcium channel blocker overdose | Mechanical ventilation; Toxidromes | Identifying a surrogate decision maker; Advanced Care Planning (ACP) |
| 3 | A patient with cirrhosis and alcohol use disorder presenting with alcohol withdrawal and upper gastrointestinal bleed | Alcohol withdrawal; upper gastrointestinal bleed; blood products | Substance use disorders and treatment protocols |
| 4 | An undocumented patient with end-stage renal disease who needs emergent dialysis | Acid-base disorders | Food insecurity and nutrition counseling |

The chief resident and unit social worker should review one of the social determinants of health (SDOH) topics for content delivery prior to each afternoon session (typically the first afternoon is a discussion of Medicare, Medicaid, and insurance subsidy programs). Below are the learning objectives for each topic session to help with session selection. The learning activities and discussion guides are detailed in the SDOH Topics guide (Appendix C).

| **Topic** | **Learning Objectives** |
| --- | --- |
| Medicare, Medicaid, and insurance subsidy programs | 1. Differentiate between resources provided by Medicare and Medicaid.  2. Identify different insurance subsidy programs for your patient populations and what they cover.  3. Tailor your approach to caring for patients under each health assistance program. |
| Advanced care planning and decision-making | 1. List and describe the different types of advanced care directives.  2. Differentiate between a living will and durable power of attorney.  3. Describe documents that complement advanced care directives.  4. Discuss differences in advanced care directives by state.  5. Identify where advanced care planning documentation lives in the medical chart.  6. Outline the approach to discussion of advanced care directives in the context of critical illness. |
| Housing insecurity | 1. Discuss the importance of addressing housing insecurity as a healthcare provider.  2. Describe what medical respite entails and how to see if a patient is eligible.  3. List community resources available for a homeless patient at the time of care transitions. |
| Substance abuse disorders and counseling | 1. Screen a patient for substance use disorders.  2. Identify social determinants of health that contribute to substance use disorders in individual cases.  3. Describe options for substance abuse disorder treatment. |
| Refugee populations and unique medical considerations | 1. Identify refugee populations in your city and the basic process through which refugees came to live in the metro area.  2. Describe the unique health considerations for refugee populations in the metro area.  3. Utilize culturally appropriate communication skills to screen refugee populations for social determinants of health. |
| Food insecurity and nutrition counseling | 1. Screen patients for food insecurity.  2. Describe the impact of food insecurity on acute and chronic medical illness.  3. Utilize resources in the hospital to address food insecurity in patients with severe nutritional deficiencies. |

Once the SDOH topic is selected, guided by the clinical details of the patient, the critical care topic is selected. The facilitator topic guides for this case material were adapted from a prior Critical Care curriculum^1^ are provided in appendix D.

**Part 4: Weekly Case-Based Discussion Sessions**

At the start of the session, the facilitator should state the critical care topic and social determinant to be discussed along with the learning objectives (these can be printed if needed). One resident should be asked to provide a brief summary of the patient history and presentation thus far. The facilitator should review the critical care teaching, emphasizing the points made in the critical care cases.

Once this has been completed, the facilitator should ask the residents to apply the CMS AHC HRSN Screening Tool to reiterate the types of questions to ask the patient and their family upon admission. If the resident caring for the patient has answers to these questions, these can be provided. Then, utilize the prompt questions for the appropriate SDOH topic to discuss why this SDOH topic is important with regards to the clinical care of the patient. Proceed to a discussion of resources available for that patient, utilizing the knowledge of the social worker or faculty present if they are a content expert. Identify questions that still need to be answered at the end of the session. Explore with the group other thoughts and emotions that come to mind when discussing the case. Conclude by having the resident who presented the case summarize their takeaway for the importance of identifying that social determinant within the case. You may have each person from the group list one new takeaway they have from the session.

Additional educational activities and online resources to consider are included in each SDOH Topic guide. Planning with your social worker, care management team member, nutritionists, and physical/occupational therapists in the ICU may bring particular value to some of these discussions:

**Part 5: Learner Assessment and Session Evaluation**

During the last session, provide the residents with the post-curriculum survey to be completed and assess for any improvements that can be made for the next iteration of the curriculum. Distribute the care management feedback form to each resident when available.

References

1. Çoruh B, Kritek P. A case-based critical care curriculum for resident physicians. MedEdPORTAL. 2012;8:9276.

2. Billioux, A., K. Verlander, S. Anthony, and D. Alley. 2017. Standardized screening for health-related social needs in clinical settings: The accountable health communities screening tool. Discussion Paper, National Academy of Medicine, Washington, DC. https://nam.edu/wp-content/uploads/2017/05/Standardized-Screening-for-Health-Related-Social-Needsin-Clinical-Settings.pdf.
