# Supplementary material for: A Case-Based Critical Care Curriculum for Internal Medicine Residents Addressing Social Determinants of Health
Source: MedEdPORTAL. 2021 Mar 18;17:11128. doi: 10.15766/mep_2374-8265.11128 (PMC8015637; doi:10.15766/mep_2374-8265.11128)
Supplement: Supplementary file 1 — Needs Assessment.docxFacilitator Guide.docxSDOH Topics Guide.docxCritical Care Cases.docxMDR Checklist.docxPre- and Postcurriculum Surveys.docxCare Team Checklist.docxAttending Checklist.docx [file mep_2374-8265.11128-s001.zip › C. SDOH Topics Guide.docx]

**1. Screening for Social Determinants of Health**

Learning Objectives

1. Describe methods to screen patients for social determinants of health.

2. Gain confidence in discussing unique barriers to health care with patients.

3. Reflect on personal experiences with patients whose access to health care is limited due to social determinants.

4. Collaborate with a team of health care providers to determine appropriate resources for patients whose health is affected by social determinants.

This discussion of screening for SDOH should be completed during the orientation session. Below is a list of the social determinants of health (SDOH) topics and associated learning objectives that can be paired with the critical care cases. The following are questions to ask patients and families upon admission to the intensive care unit (ICU) as a basic, comprehensive screen for social risk.^1^ The results of these questions should be discussed at multidisciplinary rounds.

Suggested Learning Activities

1. Group Discussion: Learners identify what topics they screen for regarding social determinants of health . Of the topics identified, compare that list to the Centers for Medicare & Medicaid Services Accountable Health Communiities Health-Related Social Needs (CMS AHC HRSN) topics (below).
2. Pair/Shar: Split the learners up into pairs or using the small group, discuss what questions they would ask under each category. Finally, review the AHR HRSN sample questions (below).

Housing Instability

- What is your housing situation today?
- Do you have problems with bug infestation, mold, inadequate heat, lead paint/pipes, appliances not working, water leaks, lack of smoke detectors?

Interpersonal Safety

- How often does anyone, including family:
- Physically hurt you?
- Talk down to you?
- Threaten you with harm?

Transportation Needs

- In the past 12 months, has lack of transportation kept you from medical appointments, meetings, work, or from getting things needed for daily living?

Food Insecurity

- Within the past 12 months, have you worried that your food would run out before you got money to buy more?

Utility Needs

- In the past 12 months has the electric, gas, oil, or water company threatened to shut off services in your home?

Questions to Prompt Discussion

- What fears do you have about asking these questions?
- Have you asked patients any of these questions previously?
  - If yes, were you surprised by the answers you heard?
- What alternative ways of wording these questions can you think of?

References

1. Billioux, A., K. Verlander, S. Anthony, and D. Alley. 2017. Standardized screening for health-related social needs in clinical settings: The accountable health communities screening tool. Discussion Paper, National Academy of Medicine, Washington, DC.

**2. Medicare, Medicaid, and Insurance Subsidy Programs**

Learning Objectives

1. Differentiate between resources provided by Medicare and Medicaid.

2. Identify different insurance subsidy programs for your patient populations and what they cover.

3. Tailor your approach to caring for patients under each health assistance program.

Suggested Learning Activities

1. Group discussion, guided by question prompts (below) and learner questions. Facilitator can go through each question with the group, go around the room asking each participant a question, or utilize think-pair-share for larger groups.

2. Print out the Medicare and Medicaid online applications and have learns fill them out from the perspective of a patient.

3. Discuss individual patients in the MICU. Present different outcomes for patients based on current physical therapy/occupational therapy recommendations, wound care, nutrition needs (enteral feeding vs parenteral nutrition) and discuss the challenges related to each insurance program.

Questions to Prompt Discussion

- How might a patient’s insurance come up during your social risk screen utilizing the CMS AHR HRSN Screening Tool?
- How might social determinants of health be barriers for patients to obtain insurance?
- What are the differences between Medicare, Medicaid, and the insurance subsidy programs you have heard of for the patient population you care for?
  - Medicaid is managed by federal and state groups, who is eligible varies based on level of income in each state. Pregnant women and children are also covered.
  - Medicare is a federal program that provides health insurance for individuals aged 65 and older or under 65 with a disability and otherwise without income (including end-stage renal disease).
    - Part A: Hospital insurance (inpatient stay, nursing facilities, hospice)
    - Part B: Medical insurance (outpatient care and physician services)
    - Part C: A and B plus additional coverage
    - Part D: Prescription drug coverage for outpatients
    - You can have both Medicare and Medicaid.
- How might this patient’s care have looked differently if they had Medicare, Medicaid, or were uninsured?
- What is the process in our hospital for an uninsured patient to obtain emergency medical insurance?
- What barriers exist for patients who are both undocumented and uninsured?
  - Are they able to be covered under an insurance subsidy program?
- What are federally qualified health centers (FQHC)?
  - These are community-based providers that receive federal funding in order to deliver primary care services to underserved patient populations.
  - They utilize a sliding fee scale and have stringent guidelines on the care they can provide.
  - Their operations are regulated by a governing board that includes patients.
- Why is it important to recognize the differences in patient insurance in the ICU setting?
- Helps provide context for care the patient was receiving or eligible for before ICU admission and will help in thinking about patient disposition after the ICU.
- ICU patients frequently require rehabilitation or advanced therapeutic care before or after hospitalization and it is important to know their insurance to understand their eligibility for certain services.

| **Insurance** | **Home Health** | **Acute Rehab** | **Sub-Acute Rehab** | **Long-Term Acute Care** | **Durable Medical Equipment** | **Oxygen** |
| --- | --- | --- | --- | --- | --- | --- |
| Medicaid | X |  |  |  | X | X |
| Medicare | X |  | X | X | X | X |
| DH* Medicare | X |  | X |  | X | X |

*DH, Denver Health (If the institution where this curriculum is implemented, has a relevant insurance coverage plan, appropriate to review here)

Resources

<https://www.medicareinteractive.org/>

<https://www.cms.gov/Outreach-and-Education/Medicare-Learning-Network-MLN/MLNProducts/Downloads/ProgramBasics.pdf>

**3. Advance Care Planning and Decision-Making**

Learning Objectives

1. List and describe the different types of advanced care directives.

2. Differentiate between a living will and durable power of attorney.

3. Describe documents that complement advanced care directives.

4. Discuss differences in advanced care directives by state.

5. Identify where advanced care planning documentation lives in the medical chart.

6. Outline the approach to discussion of advanced care directives in the context of critical illness.

Suggested Learning Activities

1. What are the differences between advanced care planning documents? Have learners work in pairs and assign them to look up different advanced care planning documents (or have these available in print for them) and explain them to the group after 5 minutes: living will, power of attorney, MOLST, and DNR order.

2. Have the learners work in pairs. Assign your state to one pair and any number of bordering states to each of the subsequent pairs. Have them look up what the surrogate decision making policy is and compare and contrast between each of these states.

Questions to Prompt Discussion

- What role did SDOH have in delineating advanced care planning for this patient?
- Who has experienced a situation where a patient does not understand the code discussion in the hospital? What were the challenges during this conversation?
- How often do you ask your patients about whether they have advanced directives on file when they come into the ICU?
- How often do you look for advanced directives in the medical chart?
- What challenges arose with advanced care planning for this patient?
- How would addressing SDOH have helped with advanced care planning for this patient?
- Can we find the advanced care planning documentation for all of our patients in the electronic medical record?
- What will you do differently knowing more information about advanced care planning?

*Advanced directives* are legal documents that allow you to spell out your wishes for end-of-life care ahead of time. Act as a surrogate to inform your family, friends, and health care professionals to avoid confusion if you cannot speak for yourself.

- Living will discusses which treatments you want if you are dying or permanently unconscious. You can accept or refuse medical care. Includes resuscitation (in the setting of cardiopulmonary arrest), artificial nutrition and hydration, dialysis, mechanical ventilation, organ/tissue donation.
- Durable power of attorney for health care (MDPOA) is a document that names your health care proxy legally. This is someone you trust to make health decisions for you if you cannot.
- DNR orders can be placed in the hospital and exist separately from DNR order for use outside the hospital, the latter of which must be signed by the patient and doctor.

*POLST, MOST, and MOLST*

POLST = Physician Orders for Life Sustaining Treatment

MOST = Medical Orders for Scope of Treatment

MOLST = Medical Orders for Life-Sustaining Treatment

- POLST/MOST forms are appropriate for those who are in their final year of life or suffering from an advanced stage terminal illness/illness they are not expected to recover from. It is complementary to and NOT considered an advanced directive. These forms vary state by state.
- Compared to advanced directives, more for the seriously ill, current care (vs future care), completed by health care professionals (rather than by the patient).
- A POLST form represents and summarizes a patient's wishes in the form of medical orders for end-of-life care. The POLST form is designed to be most effective in emergency medical situations.

*What if there is no proxy delineated?*

- In states where default surrogate decision makers are authorized, the typical order of priority is a spouse (or domestic partner in jurisdictions that recognize this status), an adult child, a parent, a sibling, and then possibly other relatives or a close friend. If more than one person has the same priority (eg, several adult children), consensus is preferred, but some states allow health care practitioners to rely on a majority decision. However, dissension among authorized decision-makers merits further counseling or consultation with an institutional ethics committee or similar resource.
- All surrogate decision makers, whether appointed by the person, by the court, or by default, have an obligation to follow the expressed wishes of the adult person and take into account the person’s values if known. Health care practitioners are responsible for honoring these wishes and values as well. If the person’s wishes and values are not known, the surrogate decision maker must always be guided by the best interests of the person.
- Health care practitioners are not required to provide treatments that are medically inappropriate, such as those that are against generally accepted health care standards. If a particular treatment is against a practitioner’s conscience but is still within generally accepted health care standards, the practitioner should try (and may be legally obligated to try) to transfer a person to another doctor or institution willing to comply.
- As a practical matter, the first step in making a treatment decision as a person's agent or surrogate decision maker is to get all the facts from health care practitioners about diagnosis, prognosis, and alternative treatments. When facing a critical treatment decision, agents and surrogate decision makers should ask themselves questions such as the following:
  - Will this treatment or test make a difference? How?
  - Do the burdens or risks of this treatment outweigh the benefits?
  - Is there hope of recovery, and, if so, what will life be like afterwards?
  - What is the goal of this treatment? Is it aligned with the patient's goals?

Resources

Where to refer patients for informed decision-making?

- National Institute on Aging: <https://www.nia.nih.gov/health/advance-care-planning-healthcare-directives>
- American Academy of Family Physicians: <https://familydoctor.org/advance-directives-and-do-not-resuscitate-orders/?adfree=true>
- National Hospice and Palliative Care Organization: <http://www.caringinfo.org/files/public/brochures/End-of-Life_Decisions.pdf>
- The Conversation Project: <http://theconversationproject.org/>
- <https://www.gyst.com/>

**4. Housing Insecurity**

Learning Objectives

1. Discuss the importance of addressing housing insecurity as a healthcare provider.

2. Describe what medical respite entails and how to see if a patient is eligible.

3. List community resources available for a homeless patient at the time of care transitions.

Suggested Learning Activity

After reviewing the questions to prompt discussion, utilize the four articles listed below and treat this session as a real-time journal club. Have the participants work in pairs, with each pair reading and evaluating one of those articles. After 10 minutes, have them explain the findings of the article to the group.

Questions to Prompt Discussion

- How often do you screen your patients for housing insecurity?
- What other social determinants go hand-in-hand with housing insecurity?
- What resources are available for our patients during transitions of care with regards to housing?
- How does caring for homeless individuals make you feel?
- What challenges do you experience in trying to ensure safe transitions of care for patients who are homeless?

Definition of Homelessness:

- People living in a place not meant for human habitation, in an emergency shelter, transitional housing, or are exiting institution where they temporarily resided for up to 90 days if they were in a shelter or place not meant for human habitation prior.
- Losing their nighttime residence (motel, hotel), lack resources or support to remain in housing.
- Families with children or unaccompanied youth with two or more moves in the past 60 days, not had a lease nor ownership interest in a housing unit in the past 60 days or are likely to remain unstably housed because of disability or barriers to employment.
- People who are fleeing domestic violence, dating violence, sexual assault, dangerous/life-threatening situations to avoid violence with no other residence nor means to other permanent housing.
- People who were in transitional housing prior to entering an institution are not considered homeless.

Why is it important to understand which patients are dealing with housing insecurity?

- Individuals who experience homelessness often have comorbid chronic disease, disability, mental health issues, substance abuse disorders; become “super-utilizers” of emergency rooms and hospitals, have higher rates of abuse/trauma and domestic violence, have increased mortality.
- Homelessness overall is an independent risk factor for death.

What evidence is there for improving health outcomes with providing housing?

Housing First Policy:

Excellent data to demonstrate that people experiencing homelessness can achieve stability in permanent housing if provided with the appropriate level of services. Studies show this yields higher housing retention rates, drives significant reductions in the use of costly crisis services and institutions, and helps people achieve better health and social outcomes.

- Lipton FR, et. al. Tenure in supportive housing for homeless persons with severe mental illness. Psychiatric Services. 2000, 51(4): 479-486.
- Larimer M, Malone D, Garner M, et al. Health Care and Public Service Use and Costs Before and After Provision of Housing for Chronically Homeless Persons with Severe Alcohol Problems. Journal of the American Medical Association, April 1, 2009, pp. 1349-1357.
- Tsai J, Gelberg L, Rosenheck RA. Changes in Physical Health After Supported Housing: Results from the Collaborative Initiative to End Chronic Homelessness. J Gen Intern Med. 2019 Sep;34(9):1703-1708.
- Baxter AJ, Tweed EJ, Katikireddi SV, Thomson H. Effects of Housing First approaches on health and well-being of adults who are homeless or at risk of homelessness: systematic review and meta-analysis of randomised controlled trials. J Epidemiol Community Health. 2019 May;73(5):379-387.

What is medical respite?

Acute and post-acute medical care for homeless persons too ill or frail to recover on the street and not ill enough to remain hospitalized.

- Respite beds are a scarce resource. Must be requested the day of discharge, may not be available every day, on weekends. Strict eligibility criteria.
- Qualifications: must have state ID, acute medical need lasting <1 month and able to complete all activities of daily living (ADLs) independently with or without DME (oxygen alone does not qualify someone).
- There are no RNs nor CNAs on site to assist with medications or any additional needs.
- Patients who require minimal assistance with ADLs are excluded.
- Difficult to arrange and maintain home health
- Patients must be sober with none or minimal substance abuse support.
- There is a high ratio of case manager to patients.
- Length of stay is limited as are links to transitional housing.
- Scarcity and over-utilization of beds has led to further rationing scenarios.

**5. Substance Abuse**

Learning Objectives

1. Screen a patient for substance use disorders.

2. Identify social determinants of health that contribute to substance use disorders in individual cases.

3. Describe options for substance abuse disorder treatment.

Suggested Learning Activities

1. Review the diagnosis and treatment of alcohol and opiate use disorder. Consider discussion of other substance use disorders that may be more prevalent in your patient population.

2. After reviewing the questions to prompt discussion, have the learners discuss specific patient cases where they have treated substance use disorders including rationale for their choice of therapy and monitoring while on therapy.

Questions to Prompt Discussion

- Who has taken care of a patient with a substance use disorder? How many of these patients have you screened for social risk? What might you expect from a systematic screen for social risk (with the CMS AHC HRSN) in these patients?
- How does caring for patients with substance use disorders make you feel?
- What are local resources for patients with substance use disorders?

*Alcohol Use Disorder*

Diagnosis: mild (2-3), moderate (4-5), or severe (6 or more) based on the number of DSM-5 criteria present

- Tolerance
- Cravings
- Withdrawal or use of alcohol to relieve withdrawal
- Drinking in larger amounts over progressively longer periods of time
- Continued drinking despite social or interpersonal problems related to alcohol use
- Continued drinking despite knowledge of physical or psychological problems related
- Recurrent drinking resulting in failure to fulfill obligations
- Giving up important activities due to alcohol use
- Recurrent drinking in hazardous situation
- Significant time spent obtaining, using, or recovering from use
- Persistent desire or unsuccessful attempts to stop or decrease quantity

1. Naltrexone

- First-line therapy.
- Can be used while patient is still drinking, one pill daily. Long-acting injectable is available for those with concerns regarding compliance.
- Wait 4-5 half lives since last opioid medication prior to initiation if no dependence.
- If opioid dependent- wait 7-10 days. LFTs< 3x upper limit of normal. Start at 25mg PO once daily x 3 days if history of anxiety or recent opioid use. Otherwise start at 50mg PO once daily.

2. Acamprosate

- Should be started after abstinence is achieved.
- Can be used in patient with hepatic impairment, contraindicated if severe renal dysfunction is present.

3. Disulfiram

- Can also be used by abstinent patients.

*Opiate Use Disorder (OUD)*

Definitions: problematic pattern of opioid use leading to clinically significant impairment or distress, manifested by two or more of the following within a 12-month period:

- Opioids are often taken in larger amounts or over a longer period than was intended
- A persistent desire or unsuccessful efforts to cut down or control opioid use
- A great deal of time is spent in activities necessary to obtain the opioid, use the opioid, or recover from its effects
- Craving, or a strong desire or urge to use opioids
- Recurrent opioid use resulting in a failure to fulfill major role obligations at work, school, or home
- Continued opioid use despite having persistent or recurrent social or interpersonal problems caused or exacerbated by the effects of opioids
- Important social, occupational, or recreational activities are given up or reduced because of opioid use
- Recurrent opioid use in situations in which it is physically hazardous
- Continued opioid use despite knowledge of having a persistent or recurrent physical or psychological problem that is likely to have been caused or exacerbated by the substance

Severity = Mild (2-3), Moderate (4-5), Severe (6 or more)

Experiencing tolerance or withdrawal while taking opioids solely under appropriate medical supervision does not meet the criteria for OUD.

Specifiers for the diagnosis include:

- In early remission – After full criteria for OUD were previously met, none of the criteria for OUD have been met (with the exception of craving) for at least three months but less than 12 months.
- In sustained remission – After full criteria for OUD were previously met, none of the criteria for OUD have been met (with the exception of craving) during a period of 12 months or longer.
- On maintenance therapy – If the individual is taking a prescribed opioid agonist or antagonist medication and none of the criteria for OUD have been met except tolerance to or withdrawal from the agonist.
- In a controlled environment – If the individual is in an environment where access to opioids is restricted.

Treatments:

Medication-assisted treatment or medication for addiction treatment (MAT)

Recommended for moderate to severe use disorder as initial treatment

Most clinical trials directly comparing MAT and psychosocial treatment favor MAT

1. Naltrexone (only after withdrawal fully completed)
   1. Long acting injectable option is available
   2. One study found significant proportion of patients cannot surmount the challenge of completing the full withdrawal from opioids required prior to treatment with naltrexone (Lee JD, Nunes EV Jr, Novo P, et al. Comparative effectiveness of extended-release naltrexone versus buprenorphine-naloxone for opioid relapse prevention (X:BOT): a multicentre, open-label, randomised controlled trial. Lancet 2018; 391:309.)
   3. Not often used first line.
   4. Sometimes used in mild disorder.
2. Methadone (opioid agonist)
   1. On average studies show slightly more efficacious than buprenorphine head to head.
   2. Higher risk for misuse and lethal overdose than buprenorphine.
3. Buprenorphine (partial agonist) – first line

Psychosocial treatment

More often as adjunct to MAT

Special groups:

- Ambivalence about treatment with ongoing opiate use = motivational interviewing
- Nonadherence = supervised medication administration with LAI naltrexone or long-acting subcutaneous buprenorphine
- Medications not allowed/available in many criminal justice settings (such as prison).
- Pregnancy = methadone and buprenorphine are safe (not teratogenic) and effective. Data for naltrexone is lacking.

**6. Refugee Populations**

Learning Objectives

1. Identify refugee populations in your city and the basic process through which refugees came to live in the metro area.

2. Describe the unique health considerations for refugee populations in your community.

3. Utilize culturally appropriate communication skills to screen refugee populations for social determinants of health.

Suggested Learning Activity

1. Discuss the definitions of a refugee and their access to health care services.

2. Have learners use their phones or computers to look up what refugee populations may reside in or around the city of their training program.

3. Have the group brainstorm barriers that may be unique to a refugee population with regards to health issues.

Questions to Prompt Discussion

- How do you know if your patient is a refugee?
- What refugee populations are present in our patient population?
- What might we expect during our SDOH screen for a refugee patient?
- What challenges do refugees face with regards to navigating health care?

Who is considered a refugee?

- A refugee is a person who is outside the country of his or her nationality due to a well-founded fear of persecution (due to race, religion, nationality, social group, political opinions for example) who is unable or unwilling to return to that country due to said fears.
- A refugee has gone through a legal process (either though an overseas process, including US embassies or refugee camps) to be granted refugee status before arrival into the US.
- Refugees are assigned a state to go to once they arrive in the US. The state-based refugee program then coordinates their resettlement.

This is different from an asylee.

- An asylum seeker is someone who flees his or her own country (for reasons similar to a refugee) to seek sanctuary in another country.
- An asylum seeker applies for asylum at the port of entry of the destination country.
- When an asylum seeker reaches the port of entry at a destination country, they ask for asylum; they can be detained, or released to a family member (many times with an ankle bracelet or other form of tracking) while waiting for asylum to be granted (a legal process involving lawyers).
- Asylees (asylum seekers who have been granted asylum legally) are not assigned a state or a resettlement agency but are given the option to enroll with a resettlement agency to go through a similar process as refugees.

Healthcare for Refugees and Community Resources

- Through the resettlement process, each refugee that comes to the US undergoes a health screening at a federally qualified health center (FQHC) within 3 months of arrival.
- This FQHC goes through a medical screening exam and labs as recommended by the Centers of Disease Ccontrol, including screening for infectious disease. These centers often have basic bloodwork done and have requested records from the patient’s prior country to aid in continuity.

Refugees face a number of challenges regarding their health, including language barriers, competing financial demands (health versus food, housing), competing demands on time (focusing on integration and work, versus health), undiagnosed chronic medical conditions due to lack of access to care in country of origin, mental health issues given exposure to trauma and warfare. All refugees start a Medicaid application within 7 days of arrival in the US with their resettlement agency. There are health coordinators at each FQHC that can help with this process.

Resources

“For Refugees.” *Department of Human Services*, 19 Apr. 2019, [www.colorado.gov/pacific/cdhs/refugees](http://www.colorado.gov/pacific/cdhs/refugees).

Jackson, J. Carey, et al. “Harvard Public Health Review: A Student Publication.” *Healthcare Recommendations For Recently Arrived Refugees: Observations from EthnoMed | Harvard Public Health Review: A Student Publication*, harvardpublichealthreview.org/case-based-recommendations-for-the-health-care-of-recently-arrived-refugees-observations-from-ethnomed/.

“Refugee Health Guidelines | Immigrant and Refugee Health | CDC.” *Centers for Disease Control and Prevention*, Centers for Disease Control and Prevention, www.cdc.gov/immigrantrefugeehealth/guidelines/refugee-guidelines.html.

**7. Food Insecurity**

Learning Objectives

1. Screen patients for food insecurity.

2. Describe the impact of food insecurity on acute and chronic medical illness.

3. Utilize resources in the hospital to address food insecurity in patients with severe nutritional deficiencies.

Suggested Learning Activities

1. Involve the nutritionist in the discussion to explain their approach to the critically ill patient

2. Discuss enteral versus parenteral feeding of ICU patients.

Questions to Prompt Discussion

- Why is food insecurity a core component of the CMS Screening Tool for SDOH?
- How often do you ask your patients about their access to food?
- What does our care management team typically offer to patients who are struggling with food insecurity? Is there a list of resources or is there online information I can access to provide patients with directly?
- How does our nutritionist or dietician counsel patients specifically in the ICU?

Is food insecurity an issue where I live for the patients I serve?

- At least 10.5% of United States households were food insecure at some point in 2019.

How can I screen for food insecurity?

- The US Department of Agriculture has an official Food Security survey.
- You can also screen patients by asking if they are on food stamps, as a number of people will respond with “No, but how can I get on them?”
- How often in the last 3 months a person has worried about not having food, or having the food run out, or not having money for food? It is important to involve a dietician if you identify a severe nutritional deficiency, as these colleagues can assist on how to maximize diet choices with a limited budget.
- Did you cut the size of a meal or skip a meal because you were worried about running out of food?
- Have you previously been hungry but didn’t eat due to fear of running out of food?

Impact of food insecurity on health

- In 2016, the US Department of Agriculture reported that 11.5% of US adults loved in food-insecure households. There are chronic diseases and health conditions associated with limited access to food, including diabetes, hyperlipidemia, hypertension, and mental health issues.
- Given limited financial resources, people with food insecurity often choose food over medications, which can further a chronic disease.
- High-calorie foods (such as fast food) are cheaper than fruits and vegetables and are a quick option for adults working multiple jobs.
- Food insecurity is a strong predictor of increased health care utilization and costs.

Resources

*The Impact of Poverty, Food Insecurity, and Poor Nutrition on Health and Well-Being*. *The Impact of Poverty, Food Insecurity, and Poor Nutrition on Health and Well-Being*, frac.org/wp-content/uploads/hunger-health-impact-poverty-food-insecurity-health-well-being.pdf.

<https://www.ers.usda.gov/topics/food-nutrition-assistance/food-security-in-the-us/>
