# Supplementary material for: A Case-Based Critical Care Curriculum for Internal Medicine Residents Addressing Social Determinants of Health
Source: MedEdPORTAL. 2021 Mar 18;17:11128. doi: 10.15766/mep_2374-8265.11128 (PMC8015637; doi:10.15766/mep_2374-8265.11128)
Supplement: Supplementary file 1 — Needs Assessment.docxFacilitator Guide.docxSDOH Topics Guide.docxCritical Care Cases.docxMDR Checklist.docxPre- and Postcurriculum Surveys.docxCare Team Checklist.docxAttending Checklist.docx [file mep_2374-8265.11128-s001.zip › D. Critical Care Cases.docx]

**1. Shock and Vasopressors**

Adapted from Çoruh B, Kritek PA. A case-based critical care curriculum for resident physicians. *MedEdPORTAL.* 2012;8:9276. <https://doi.org/10.15766/mep_2374-8265.9276>

Learning Objectives

1. List the common types of shock and explain their pathophysiology.
2. Describe the role of crystalloid and colloid in resuscitation.
3. Describe the mechanism of action of the commonly used vasopressors.
4. Select vasopressor(s) appropriate to the clinical scenario.

Case

You are called to the general surgical floor to evaluate Mr. G, a 72 year old man with a history of colon cancer who underwent a total abdominal colectomy with end ileostomy four days prior. He was progressing towards discharge in the next day or two, when he was found by his nurse to be hypotensive and tachycardic.

On exam, temperature is 37.9˚C, BP is 70/42, HR 132, RR 26, O2 saturation 96% on 2 L/min via nasal cannula. He is drowsy and difficult to arouse. Heart is tachycardic, but regular. Lungs are clear, though diminished in the bases. His abdominal exam is notable for a healing scar and a new ileostomy. He is nondistended and nontender. A Foley catheter is draining clear, yellow urine.

Questions

**Define shock.**

Shock is a state of diminished oxygen delivery, increased oxygen consumption, and/or inadequate oxygen utilization resulting in cellular and tissue hypoxia. Hypotension does not equal shock – which is important to differentiate when treating shock as the goal should be improving end-organ perfusion.

**What is the pathophysiology of different categories of shock and what is on your differential diagnosis?**

Four types are recognized, although many patients have more than one (multifactorial). Undifferentiated shock refers to a situation where the etiology of shock is unknown.

| **Type** | **Preload**  **(CVP, PCWP)** | **Cardiac Output** | **Afterload**  **(SVR)** | **SvO_2_** |
| --- | --- | --- | --- | --- |
| Distributive | Low | High | **LOW** | High |
| Cardiogenic | High | **LOW** | High | Low |
| Hypovolemic | **LOW** | Low | High | Low |
| Obstructive | **DEPENDS ON ETIOLOGY** | Low | High | Low |

*Primary derangement noted in capital, bold letters.

Forms of distributive shock include: septic, neurogenic, anaphylactic, SIRS, drug and toxin-related, and endocrine. Obstructive shock is most commonly related to extracardiac causes of pump failure and results in poor right ventricular output.

| **Distributive** | | **Cardiogenic** | | | **Hypovolemic** | | **Obstructive** | |
| --- | --- | --- | --- | --- | --- | --- | --- | --- |
| Septic | Non-septic | Myopathy | Arrhythmia | Mechanic | Hemorrhage | Other | Vascular | Mechanical |
| Gram +  Gram –  Fungal  Viral  Parasitic  MB | Burns  Trauma  Pancreatitis  Neurogenic  Anaphylaxis  Liver failure  Transfusion  TSS | MI (>40%)  RV infarct  HFrEF  Stunning  Advanced sepsis  Myocarditis  Contusion  BB overdose | Afib  Aflutter  SVT  Vtach  Vfib  CHB  Mobitz II | AVI  MVI  Valve rupture  cMVS  A/VSD  Aneurysm  Myxoma | Trauma  GIB  Uteral/vaginal bleed  RP hematoma  A-E fistula  AA rupture | Diarrhea  Vomiting  Skin loss  3^rd^ spacing  Renal loss | PE  pHTN  TV stenosis  PV stenosis  Air embolus | Tension PTX  Hemothorax  Tamponade  Pericarditis Restrictive CM  High PEEP  IAH  Aorto-caval compression |

**Mixed:**

Endocrine 🡪 adrenal insufficiency, thyrotoxicosis, myxedema coma

Metabolic 🡪 acidosis, hypothermia

Polytrauma

Poisonings

Consider if Mr. G is bleeding post-operatively, if he is having a post-operative myocardial infarction or arrhythmia, pulmonary embolus given known malignancy, alternative prior valvular pathology (critical aortic stenosis), post-operative infection or hospital-acquired infection, or anaphylaxis to new exposure in the hospital.

**What exam findings and measurements will help you sort out what type of shock is present?**

- Preload: Jugular venous pressure, central venous pressure, pulmonary capillary wedge pressure, straight-leg raise
- Cardiac Output: Capillary refill, mental status, urine output, echocardiography
- Afterload: Temperature, systemic vascular resistance
- Check SVO2 if a central or pulmonary arterial catheter is in place

**Shock can be characterized by a supply-demand problem: increased demand (O2 consumption) without sufficient supply (O2 delivery). How can we express oxygen delivery and oxygen consumption?**

SUPPLY = Oxygen delivery

*Determined by Cardiac Output (HR x SV) + Arterial Oxygen Content (Hb and affinity)

*Stroke Volume determined by preload, afterload, and myocardial contractility.

*Systemic vascular resistance is determined by vessel length, viscosity, and vessel diameter.

DO2 = CO x CaO2

= CO x [(1.34 mL O2/g Hb) (Hb in g/dL) (SaO2) + 0.003 (PaO2)]

DEMAND = Oxygen consumption

VO2 = CO x (CaO2-CvO2)

= CO x [[(1.34) (Hb) (SaO2) + 0.003 (PaO2)] – [(1.34) (Hb) (SvO2) + 0.003 (PaO2)]

Venous oxygen saturation is slightly different in the periphery versus central (ScvO2) versus from the pulmonary artery (SvO2). ScvO2 has higher O2 extraction (blood from brain) whereas

mixed venous blood in PA has IVC blood, which has more O2, making SvO2 slightly higher. Normal O2 extraction is about 25-30%, therefore a normal ScvO2 is 65-70%. Low ScvO2 indicates impaired tissue oxygenation (<65%) whereas high ScvO2 indicates inadequate perfusion or hyperdynamic flow.

**Mr. G is now in the ICU and is febrile to 38.7˚C. Labs reveal a leukocytosis with left shift, a rise in his creatinine from 0.8 to 1.3, HCO3 of 17, and a lactate of 4.7. You suspect septic shock and place an internal jugular central venous catheter; CVP is 3. What now?**

Fluids, given in well-defined (500 or 1000 mL) boluses. The volume given is more important than the type of fluids. Many studies have looked at the efficacy of crystalloid versus colloid and none have shown superiority of one type of fluid over another. Two examples:

- SAFE (Saline versus Albumin Fluid Evaluation)^1^: 6997 patients randomized to 4% albumin versus normal saline for up to 28 days. No difference in mortality. Among those with severe sepsis (18% of patients), no difference in outcome.
- VISEP (Efficacy of Volume Substitution and Insulin Therapy in Severe Sepsis)^2^: 537 patients randomized to receive 10% pentastarch or modified Lactated Ringers for resuscitation. No difference in 28-day mortality, but higher rates of acute kidney injury and need for renal replacement therapy in pentastarch group with trend toward increased 90-day mortality in this group as well.

**After adequate fluid resuscitation (CVP is now 12), Mr. G remains hypotensive with mean arterial pressure of 51. You decide to start vasopressors. List the commonly used vasopressors, their mechanisms of action, and their hemodynamic effects.**

| **Drug** | **Alpha-1** | **Beta-1** | **Beta-2** | **Dopamine** | **V1** | **PDE inhibitor** | **Notes** |
| --- | --- | --- | --- | --- | --- | --- | --- |
| Norepinephrine | ++++ | +++ |  |  |  |  | a1 > B1, B2  No reflex bradycardia  #1 Sepsis/Distributive Shock  #1 Cardiogenic Shock  #1 Hypovolemic Shock  HRS |
| Epinephrine | +++ | +++ | ++ |  |  |  | a1, B1 > B2  Inotropy and chronotropy  #1 Anaphylactic Shock  #2 Septic Shock  A > B as dose increases  May induce tachyarrhythmia |
| Dopamine | ++ | +++ | + | + |  |  | Dose-dependent: DA, a1, B1  1-2, D1 vasodilation  5-10, B1 increase SV  >10, A1 vasoconstriction |
| Phenylephrine | ++++ |  |  |  |  |  | Pure vasopressor: a1, a2  Aortic Stenosis  No tachyarrhythmia induced |
| Vasopressin |  |  |  |  | +++ |  | v1, v2  #2/3 vasopressor  Pure vasoconstriction |
| Dobutamine |  | +++ | + |  |  |  | B1 > B2, weak a1  Inotropy and chronotropy  Afterload reduction  Cardiogenic Shock  Decompensated HF |
| Milrinone |  |  |  |  |  | +++ | Inotropy and chronotropy  Decompensated HF |
| Isoproterenol |  | ++++ | ++++ |  |  |  | Inotropy and chronotropy  Hypotension 2/2 bradycardia |

**You decide to start norepinephrine as your first line agent for septic shock. Mr. G remains hypotensive despite high-dose norepinephrine and your fellow suggests adding vasopressin; why?**

Vasopressin (VP) stores are depleted in sepsis (reasons unclear), so it makes intuitive sense that vasopressin may help in septic shock. Studies to date have only revealed that vasopressin in useful as a catecholamine-sparing agent.

The VASST trial by Russell et al. in 2008 randomized 778 patients already on a minimum dose of norepinephrine (NE) to receive either low-dose vasopressin or higher doses of norepinephrine.

- No significant difference in 28-day mortality (35.4% with VP vs. 39.3% with NE).
- No significant difference in serious adverse events (10.3% with VP vs. 10.5% with NE).
- In patients with less severe septic shock, 28 day mortality rate was lower in the VP group (26.5% VP vs. 35.7% NE).

Notably, a randomized trial of dopamine versus norepinephrine^4^ indicated that dopamine had no advantage as the first line agent and induced more arrhythmias, associated with an increased 28-day rate of death among patients with cardiogenic shock.

**Mr. G is now achieving an adequate MAP with norepinephrine and vasopressin, but he develops atrial fibrillation with rapid ventricular response that is refractory to DC cardioversion, amiodarone, and digoxin. What pressor change might be helpful in this situation?**

Consider discontinuing norepinephrine in favor of phenylephrine for its pure alpha effect.

References

1. Çoruh B, Kritek PA. A Case-Based Critical Care Curriculum for Resident Physicians. *MedEdPORTAL.* 2012;8. *Adapted by the authors with permission.*

2. The SAFE Study Investigators. A Comparison of Albumin and Saline for Fluid Resuscitation in the Intensive Care Unit. N Engl J Med 2004;350:2247-2256.

3. Brunkhorst FM, et al. Intensive insulin therapy and pentastarch resuscitation in severe sepsis. N Engl J Med. 2008;358(2):125-39.

4. Russell JA, et al. Vasopressin versus norepinephrine infusion in patients with septic shock. N Engl J Med. 2008;358(9):877-87.

5. De Backer D, Biston P, Devriendt J, et al. Comparison of dopamine and norepinephrine in the treatment of shock. N Engl J Med 2010;362:779-789.

6. Vincent JL, De Backer D. Circulatory shock. N Engl J Med 2013; 369:1726.

**2. Sepsis**

Adapted from Çoruh B and Kritek PA.

Learning Objectives

1. Define sepsis and discuss the new classification of this spectrum of disease.
2. Outline an approach to managing septic shock including antimicrobial therapy and intravascular volume resuscitation.

Case

The MICU team is called to admit Mr. A, a 68-year-old man who is sent from his nursing home for fever and tachypnea. He has been residing in a nursing home ever since a stroke six months prior. Other medical history includes hypertension, hyperlipidemia, and type 2 diabetes. He is unable to provide further history due to tachypnea and obtundation.

Initial vital signs: T is 39.1˚C, BP 77/30, HR 132, RR 32, and O2 sat 100% on a nonrebreather with a GCS of 8. He is intubated and sedated before your arrival in the ED. On your exam, lungs are notable for right-sided crackles, no wheezing. Heart is tachycardic, but regular. Abdominal exam is benign. A Foley catheter has been placed with minimal urine output.

Labs reveal Na 138, K 4.1, Cl 98, HCO3 17, BUN 46, Cr 2.7, glucose 160, total bilirubin 1.1

WBC 17,000 with 12% bands, Hct 39%, platelets 302,000

Lactate 6.2 A

BG post-intubation: 7.28/38/120/17 on FiO2 1.0, PEEP 5

CXR with dense RLL opacity

Questions

**How would you classify Mr. A’s illness in the spectrum of sepsis?**

Sepsis represents a spectrum of disease involving infection and can be so severe as to cause shock and multiorgan dysfunction syndrome (MODS). Prior definitions of sepsis used the systemic inflammatory response syndrome (SIRS) criteria to assess for sepsis, however recent opinion of task forces generated by national societies including the Society of Critical Care Medicine (SCCM) and the European Society of Intensive Care Medicine (ESICM) no longer use the SIRS criteria, given lack of specificity for an infectious process. Severe sepsis is also no longer an accepted terminology. However, Medicare and Medicaid do still use the SIRS, sepsis, and severe sepsis definitions.

Critical care societies now place emphasis on the identification of early sepsis, specifically in guidelines through utilization of a modified version of the Sequential (Sepsis-related) Organ Failure Assessment score (SOFA) called the quickSOFA (qSOFA)^1-3^. It is easy to calculate, using three components, one point for each:

- Altered mentation or encephalopathy
- Respiratory rate ≥ 22 per minute
- Systolic blood pressure ≤100 mmHg

The score was initially studied in patients suspected of having sepsis who were located outside of the ICU and subsequent studies of ED and ICU patients have yielded conflicting results. The full SOFA score is well-validated and has a better predictive value (as well as more variables needed to calculate as below)^4^. Individuals with scores of 15 or more have a mortality rate of 90%.

- Partial pressure of arterial oxygen (PaO2) / fraction of inspired oxygen (FiO2) ratio aka the P/F ratio (including whether mechanical ventilation is required)
- Platelet count
- Serum total bilirubin
- Serum creatinine or urine output
- Glasgow Coma Scale
- Hypotension and vasopressor requirement

SOFA Scoring and Mortality:

0-6 🡪 less than 10%

7-9 🡪 15-20%

10-12 🡪 40-50%

13-14 🡪 50-60%

15 🡪 greater than 80%

15-24 🡪 greater than 90%

*Sepsis* is defined as life-threatening organ dysfunction caused by a dysregulated host response to an infection, with organ dysfunction classified as an increase of two or more points on the full SOFA score. The presence of definite infection is per physician judgment.

*Septic shock* is classified as vasodilatory or distributive shock with greater risk of mortality overall (≥40% versus ≥10%) requiring vasopressors to maintain a mean arterial pressure (MAP) ≥65 mmHg and have a lactate >2 mmol/L.

For our patient, the qSOFA score was 3 and the full SOFA score (assuming the patient was shortly started on a vasopressor at a moderate dose) is 13, indicating a 60% hospital mortality.

**What are potential sources of infection and how do we assess for them? What antibiotics will you choose?**

The obvious source here is pulmonary given tachypnea, hypoxemia, and a compatible CXR finding. Consider other sources including skin, urine, biliary (e.g., cholangitis), and GI (e.g., C. difficile colitis). Initial workup should include sputum culture, blood cultures x 2, urinalysis and urine culture, LFTs, and stool PCR for C. difficile if diarrhea is present.

Overwhelmingly, evidence suggests that EARLY and APPROPRIATE antibiotics and source control decrease mortality. The key is to get it right up front, then to de-escalate after 48-

72 hours, when microbiologic data is available. The Surviving Sepsis Campaign calls for antibiotics within the first hour of presentation after cultures have been obtained. Don’t forget to evaluate for undrained pockets of infection that cannot be treated with antibiotics alone.

In this case, the patient is from a nursing home and should be covered for a health-care

associated pneumonia. In addition to covering the usual pneumonia suspects, the patient needs

coverage for MRSA, Pseudomonas, and other MDR organisms.

**What is “early goal-directed therapy” and why do we use this strategy in septic patients?**

Rivers et al.^5^ randomized 263 patients with severe sepsis and septic shock to early goal-directed therapy (EGDT) in the first six hours vs. usual care:

- Inclusion criteria: At least 2 SIRS criteria and SBP < 90 mm Hg despite a bolus of fluids (20-30 mL/kg over 30 minutes) or lactate ≥ 4
- Exclusion criteria: <18 years of age, pregnancy, acute stroke, acute coronary syndrome, pulmonary edema, status asthmaticus, primary cardiac dysrhythmia, contraindication to central venous catheter placement, GI bleed, seizure, drug overdose, burns, trauma, need for surgery, active cancer, immunosuppression, do-not-resuscitate (DNR) status
- Findings: In hospital mortality: 30.5% (EGDT) vs. 46.5% (control), p=0.009
- Patients receiving EGDT also had higher mean ScvO2, lower lactate, lower base deficit, higher pH, and lower mean APACHE II scores

**How will you approach fluid resuscitation for Mr. A’s hypotension?**

Aggressive, rapid IV fluids is a cornerstone of treating sepsis and intravascular hypotension. Initial therapy should include a 30 mL/kg bolus of fluids within the first three hours of presentation to restore tissue perfusion. If patients demonstrate clinical or hemodynamic indicators of fluid responsiveness (example: straight leg raise positive) after the initial bolus, additional IV fluid can be administered. This may continue until blood pressure and tissue perfusion are acceptable, no further strides are made towards improving these variables, or pulmonary edema occurs.

Randomized trials have found no convincing difference between using crystalloid solutions (including saline and lactated ringer’s) and albumin solutions in treating sepsis or septic shock^7^. The cost of albumin can be prohibitive with the associated lack of benefit, although some may reach for this solution when hyperchloremia with crystalloid solutions becomes limiting. Pentastarch, hydroxyethyl starch, and hypertonic saline have no role in intravascular volume resuscitation in sepsis.

**After 6 L of crystalloid, Mr. A has a mean arterial pressure of 47. What is your next step?**

Vasopressors. The Rivers study used either dopamine or norepinephrine. Multiple head-to-head trials of norepinephrine against phenylephrine, vasopressin, terlipressin, and epinephrine suggest norepinephrine should be first line. The SOAP II trial demonstrated that norepinephrine may be superior and/or have less side effects than dopamine (mainly tachyarrhythmias)^6^. Vasopressin can be added as a catecholamine-sparing pressor if needed or to reduce the dose of norepinephrine and epinephrine can be used for refractory hypotension.

References

1. Çoruh B, Kritek PA. A Case-Based Critical Care Curriculum for Resident Physicians. *MedEdPORTAL.* 2012;8. *Adapted by the authors with permission.*

2. Singer M, Deutschman CS, Seymour CW, et al. The Third International Consensus Definitions for Sepsis and Septic Shock (Sepsis-3). JAMA 2016; 315:801.

3. Shankar-Hari M, Phillips GS, Levy ML, et al. Developing a New Definition and Assessing New Clinical Criteria for Septic Shock: For the Third International Consensus Definitions for Sepsis and Septic Shock (Sepsis-3). JAMA 2016; 315:775.

4. Seymour CW, Liu VX, Iwashyna TJ, et al. Assessment of Clinical Criteria for Sepsis: For the Third International Consensus Definitions for Sepsis and Septic Shock (Sepsis-3). JAMA 2016; 315:762.

5. Vincent JL, de Mendonca A, Cantraine F, et al. Use of the SOFA score to assess the incidence of organ dysfunction/failure in intensive care units: results of a multicenter, prospective study. Working group on "sepsis-related problems" of the European Society of Intensive Care Medicine. Crit Care Med 1998; 26:1793.

6. Rivers E, et al. Early goal-directed therapy in the treatment of severe sepsis and septic shock. N Engl J Med. 2001;345(19):1368-77.

7. De Backer D, Biston P, Devriendt J, et al. Comparison of dopamine and norepinephrine in the treatment of shock. N Engl J Med 2010;362:779-789.

8. Finfer S, Bellomo R, Boyce N, et al. A comparison of albumin and saline for fluid resuscitation in the intensive care unit. N Engl J Med 2004; 350:2247.

**3. Sedation, Analgesia, and Delirium**

Adapted from Çoruh B and Kritek PA.

Learning Objectives

1. List the commonly used sedatives and analgesics in the ICU and their side effects.
2. Explain how spontaneous awakening trials are performed and their purpose.
3. Describe how to assess for sedation and delirium in the ICU using RASS and CAM-ICU, respectively.
4. Describe non-pharmacologic and pharmacologic ways to treat delirium.

Case

Ms. B is a 67-year-old woman who presents with two days of fevers, dyspnea, and cough productive of yellow sputum. She has a history of type 2 diabetes and hypertension but is otherwise healthy. Medications include metformin, HCTZ, lisinopril, and ASA.

Initial vital signs are temperature of 39.4˚C, BP 132/80, HR 114. O2 sat is 87% on a non-rebreather mask and she is intubated in the ED for hypoxemic respiratory failure. Labs are notable for creatinine of 2.4 (baseline 0.8) and leukocytosis with left shift. LFTs are normal. CXR reveals multilobar pneumonia.

Questions

**What are commonly used sedatives and analgesics in the ICU and their side effects? What agents will you choose for this patient?**

Sedatives:

- Propofol (5 mcg/kg/minute or 0.3 mg/kg/hour initial infusion; increase 5-10 mcg/kg/minute every 5-10 minutes until desired sedation; usual maintenance 5-50 mcg/kg/minute)
  - MOA: unknown, potentiates GABA_A_ receptors and blocks sodium channels
  - Physiologic effects: hypotension (decreases SVR), bradycardia, decreased ICP, respiratory depression
  - Benefits: short acting, quick on and off
  - Adverse effects: hypotension, elevated triglycerides, Propofol infusion syndrome (lactic acidosis, rhabdomyolysis, renal failure, and circulatory collapse) with prolonged use at high doses
- Lorazepam or Midazolam
  - MOA: GABA_A_ agonist
  - Physiologic effects: hypotension (decreased SVR) at high doses, respiratory depression
  - Adverse effects: delirium, accumulation in hepatic insufficiency, propylene glycol toxicity (with lorazepam, in the solution and causes gap acidosis)
  - OVERALL should be avoided when able, midazolam is shorting acting so preferred over lorazepam
- Dexmedetomidine (load with 1mcg/kg over 10 minutes followed by 0.2-0.7 mcg/kg/hour)
  - MOA: alpha_2_ agonist
  - Physiologic effects: hypotension (decreased SVR), bradycardia, minimal respiratory depression
  - Benefits: decreased opiate and benzodiazepine use, facilitates extubation in patients who fail due to agitation, data for shorter length of stay in cardiac surgery patients
  - Adverse effects: hypotension, bradycardia, cost
- Ketamine (0.1-0.5 mg/kg initial infusion followed by 0.05-0.4 mg/kg/hour)
  - MOA: noncompetitive NMDA antagonist (at sub-anesthetic doses also opioid, muscarinic, catecholamine agonist as well as nicotinic blockade)
  - Physiologic effects: analgesia, amnesia, and sedation; sympathetic stimulation (however patients who are depleted of catecholamines at increased risk for shock); bronchodilation; increases ICP (sympathetic stimulation)
  - Benefits: maintains cardiac output and mean arterial pressure without inhibition of respiratory drive, preserves protective airway reflexes
  - Adverse effects: sympathetic stimulation (increased HR and myocardial O2 demand), hallucinations, delirium, dissociative effects, hypersalivation, nausea/vomiting
  - NOT APPROVED for ICU use for sedation

Analgesics:

- Fentanyl: safest in hepatic and renal dysfunction
- Morphine: can cause hypotension, contraindicated in renal dysfunction due to accumulation of active metabolite
- Hydromorphone: can cause hypotension, safe in renal dysfunction
- Remifentanil: ultra-short acting, metabolized by tissue esterases
  - Hypotension, bradycardia, increased ICP

Ms. B is likely to remain intubated > 24 hours, so midazolam and fentanyl would be a reasonable choice. Avoid morphine as an analgesic due to presence of renal failure.

**How will we assess Ms. B’s level of sedation and what is your goal for sedation?**

One way of monitoring sedation setting sedation goals is the Richmond Agitation Sedation Scale (RASS). For Ms. B, your RASS goal may be 0 to -1. Patients on more uncomfortable ventilator modes (e.g., low tidal volume, inverse I:E ratio) may require deeper sedation.

| **Score** | **Term** | **Description** |
| --- | --- | --- |
| +4 | Combative | Overtly combative, violent, imminent danger to staff |
| +3 | Very agitated | Pulls on, removes tubes/catheters, aggressive to staff |
| +2 | Agitated | Frequent non-purposeful movement or patient-ventilator dyssynchrony |
| +1 | Restless | Anxious or apprehensive, not aggressive |
| 0 | Alert and calm |  |
| -1 | Drowsy | Not fully alert, sustained awakening >10 seconds, eye contact to voice |
| -2 | Light sedation | Briefly (<10 seconds) awakens with eye contact to voice |
| -3 | Moderate sedation | Any movement to voice (but no eye contact) |
| -4 | Deep sedation | No response to voice, arouses to physical stimulation |
| -5 | Unarousable | No response to voice nor physical stimulation |

**The next morning, Ms. B undergoes a spontaneous awakening trial (SAT). How is this performed and what are the potential benefits?**

Avoiding excess sedation is crucial in critically ill patients, as this may unnecessarily prolong mechanical ventilation and other invasive treatments. Two methods have been proven in randomized trials to decrease the duration of mechanical ventilation and related complications:

1. Intermittent infusions: An observational study^1^ of 242 patients compared duration of mechanical ventilation among patients with continuous versus intermittent sedative-analgesic infusions or none on a nursing protocol.

- Intermittent infusions or no medication led to shorter duration of mechanical ventilation (median of 56 hours) than continuous infusion (median of 185 hours).

2. Daily interruption and nursing protocolized sedation

- A trial^2^ of 128 mechanically ventilated patients on continuous sedative-analgesics randomized to conventional management (clinician decides how sedatives are titrated) or daily spontaneous awakening trials.
  - Interruption group had shorter mechanical ventilation (4.9 versus 7.3 days) and length of stay in ICU (6.4 versus 9.9 days); fewer neurodiagnostic tests as well (9% vs 27%).
  - Single center trial, ventilator weaning not standardized.
- A second trial in 2008^3^ randomly assigned 336 patients to daily SAT and SBT versus usual care + daily SBT; primary endpoint was breathing without mechanical ventilation.
  - Days breathing without assistance was less in the intervention group (14.7 versus 11.6 days) as well as median ICU LOS (9.1 versus 12.9 days) and median hospital LOS (14.9 versus 19.2 days).
  - NNT was 7.4
  - Less cognitive impairment at three months (ARR 20%) in a follow-up study although no different at 12 months.
- Meta-analysis of six trials^4^ demonstrated protocolized sedation (algorithm or daily interruption) was associated with reduced overall mortality (15%), length of hospital stay (3.5 days), and tracheostomy (31%). No difference in duration of mechanical ventilation and rate of self-extubation or re-intubation.

**How will you assess for delirium in Ms. B?**

The Confusion Assessment Method for the ICU (CAM-ICU) is one validated tool that is used in concert with a sedation assessment.

A two-step approach:

1. Sedation assessment (RASS)

- If RASS is -4 or -5, stop and re-assess patient later
- If RASS is above -4 (-3 to +4), then proceed to Step 2

2. Delirium assessment (CAM-ICU)

A. Acute onset of mental status changes or a fluctuating course AND

B. Inattention AND EITHER

C. Disorganized thinking OR Altered Level of Consciousness

CAM-ICU Testing:

A. Acute onset or fluctuating course -> if NO, stop. No delirium.

B. Inattention

- Read the letters SAVEAHAART; ask patient to squeeze on “A”
- Error if patient fails to squeeze on “A” or squeezes on other letters
- If <3 errors, stop. No delirium.

C. Altered level of consciousness.

- Assess RASS, if anything other than 0, patient is delirious.

D. Disorganized Thinking

- Ask patient:
- “Will a stone float on water?”
- “Are there fish in the sea?”
- “Does one pound weigh more than two pounds?”
- “Can you use a hammer to pound a nail?”
- Ask patient to follow commands: “hold up this many fingers.”
- If < 2 errors, stop. No delirium.

**On hospital day #3, Ms. B screens positive for delirium using the CAM-ICU. What are non-pharmacologic and pharmacologic ways to treat delirium?**

Non-pharmacologic

- Ensure daily spontaneous awakening trials
- Continually re-orient the patient
- Encourage family/friends at the bedside
- Early mobilization
- Promote effective sleep/wake cycles, minimize noise and stimulation at nights
- Timely removal of catheters and physical restraints
- Ensure the use of glasses and hearing aids
- Minimize benzodiazepines for sedation

Pharmacologic (guidelines do not recommend one over the other given lack of adequate evidence)

- Haloperidol (no evidence for reduction of mechanical ventilation, duration of delirium)
  - Adverse effects: extra-pyramidal symptoms (EPS), prolonged QTc and associated torsades de pointes
  - Monitoring: baseline and routine EKG, caution with drug interactions (ex. amiodarone, quinolones, macrolides, azoles)
- Olanzapine: causes less EPS, not available in intravenous form

References

1. Çoruh B, Kritek PA. A Case-Based Critical Care Curriculum for Resident Physicians. *MedEdPORTAL.* 2012;8. *Adapted by the authors with permission.*

2. Brook AD, Ahrens TS, Schaiff R, et al. Effect of a nursing-implemented sedation protocol on the duration of mechanical ventilation. Crit Care Med 1999; 27:2609.

3. Kress JP, et al. Daily interruption of sedative infusions in critically ill patients undergoing mechanical ventilation. N Engl J Med. 2000;342(20):1471-7.

4. Girard TD, et al. Efficacy and safety of a paired sedation and ventilator weaning protocol for mechanically ventilated patients in intensive care (Awakening and Breathing Controlled trial): a randomised controlled trial. Lancet. 2008;371(9607):126-34.

5. Minhas MA, Velasquez AG, Kaul A, et al. Effect of Protocolized Sedation on Clinical Outcomes in Mechanically Ventilated Intensive Care Unit Patients: A Systematic Review and Meta-analysis of Randomized Controlled Trials. Mayo Clin Proc 2015; 90:613.

**4. Non-Invasive Positive Pressure Ventilation**

Adapted from Çoruh B and Kritek PA.

Learning Objectives

1. Describe indications for BPAP as compared to CPAP.
2. List the contraindications to BPAP and CPAP.
3. Explain the evidence for the use of NIPPV in respiratory failure due to COPD exacerbations.

Case

The MICU team is called to the ED to evaluate Mr. T, a 69 year old man with a history of severe COPD (FEV1 <1 L) on 2 L/min continuous home oxygen and atrial fibrillation. He notes several days of worsening cough and dyspnea and states his baseline white sputum is now green. No fevers, chills, N/V, abdominal pain, diarrhea, or myalgias. His granddaughter recently had an upper respiratory illness that resolved without treatment.

On exam, temperature is 37.0˚C, BP is 142/84, HR 104 and irregular, RR 26, O2 saturation 100% on a non-rebreather. He appears to be in mild distress and is speaking in short sentences. He is also lethargic, but arousable. Heart is irregularly irregular without murmurs. Lung exam is notable for diffuse wheezing and prolonged expiratory phase. The remainder of the exam is unremarkable.

Labs reveal Na 133, HCO3 32, BUN 20, Cr 0.7 WBC 12,000, Hct 44%, platelets 371,000

CXR reveals large lung volumes and flattened diaphragms, there are no focal opacities

Questions

**What is your assessment of Mr. T’s presentation and how will you treat him?**

The patient appears to have a COPD exacerbation as evidenced by increased dyspnea, increased cough, and change in volume/appearance of sputum. Most common triggers for exacerbations are infections (bacterial and viral) and environmental pollutants.

Initial steps:

- Turn down the oxygen. Your goal should be to treat hypoxemia while avoiding any secondary hypercapnia from worsening V/Q mismatch. The issue here is not diminishing hypoxic drive, but rather interfering with hypoxic vasoconstriction, leading to perfusion of poorly ventilated lung. Aim for a PaO2 of 60-70 mm Hg and SpO2 of 90-94%.
- Scheduled beta-adrenergic agonists and anticholinergic agents: no difference between MDI and nebulizers, although patients in respiratory distress often have difficulty using MDIs appropriately, even with a spacer.
- Glucocorticoids: no consensus on route (oral vs. IV), dose, or frequency. GOLD guidelines advise prednisone 40mg once daily, regimens however can range from 30-60mg daily or methylprednisolone 60-125mg two to four times daily. Literature states this improves symptoms, lung function, and decreases length of hospitalization. The REDUCE trial^1^ demonstrated that a short course (5 days) is non inferior to longer courses (14 days).
- Sputum culture is usually not useful for identifying bacterial infection unless symptoms fail to respond to initial therapy.
- Consider viral panel testing.
- Consider additional testing including troponin to evaluate tachycardia or myocardial ischemia, BNP for heart failure, an D-dimer to assess for PE.

**Does Mr. T need antibiotics? If so, which antibiotic will you choose?**

Antibiotics for COPD exacerbations are often debated, and most guidelines do recommend them in patients requiring hospitalization or with complicated COPD (age > 65 years, FEV1<50%, 2 or more exacerbations a year, and cardiac disease). GOLD guidelines recommend antibiotics for patients with a severe exacerbation requiring mechanical ventilation (noninvasive or invasive) or for patients with increased sputum purulence + either increased dyspnea or increased sputum volume.

You want to cover the usual bacterial pneumonia suspects, so for hospitalized patients this usually means a respiratory fluoroquinolone or a third generation cephalosporin + macrolide. If Pseudomonas is suspected, antibiotics should be broadened.

**An arterial blood gas is obtained on a non-rebreather: 7.24/74/249/31. How would you interpret Mr. T’s ABG?**

Acute on chronic respiratory acidosis as evidenced by elevated serum HCO3 of 32. This is an important point as it will affect your treatment goals for his respiratory failure. You do not want to “normalize” his pCO2 to 40 mm Hg in this case, but should aim instead to normalize the pH.

**You next need to address the patient’s respiratory failure. What are indications and contraindications for non-invasive positive pressure ventilation (NPPV) and is this an appropriate scenario for its use?**

Indications for NPPV

- Hypercarbic respiratory failure: best studied in COPD exacerbations, consider use in post-operative respiratory failure and after discontinuation of mechanical ventilation in patients with COPD.
- Hypoxemic respiratory failure: cardiogenic pulmonary edema, immunocompromised patients with fever and infiltrates on CXR (use CPAP in this setting).
- Chronic uses: obesity hypoventilation syndrome, restrictive thoracic disorders, neuromuscular weakness.
- Consider use for palliation in patients who decline intubation while considering overall goals of care.

*NPPV is not a good therapy for failed extubation (in contrast to extubating to NPPV in COPD) as studies have shown increased morbidity and mortality in this scenario.

*Note that NPPV or CPAP should be used carefully in cases of hypoxemic respiratory failure. There is some evidence that use of these modalities delays intubation and results in worse outcomes.

Contraindications to NPPV:

- Cardiac or respiratory arrest
- Inability to protect airway or impaired consciousness
- Excessive secretions
- Facial surgery/trauma/deformity
- Recent esophageal anastomosis

Mr. T is an excellent candidate for NPPV.

**Which noninvasive mode (NPPV or CPAP) is appropriate for this clinical scenario and what initial settings will you choose?**

1) CPAP: a constant level of pressure applied throughout the respiratory cycle to stent open the upper airway (e.g., OSA) or to prevent alveolar collapse and increase the surface area for gas exchange.

Affects OXYGENATION

2) Bi-level positive airway pressure: CPAP + an inspiratory pressure.

Affects OXYGENATION and VENTILATION

CPAP is helpful for OSA and cardiogenic pulmonary edema. In the latter, CPAP improves oxygenation both by keeping alveoli open and by decreasing preload and afterload to improve LV function. During systole, the increased intrathoracic pressure present because of CPAP decreases RV and LV preload, improving mechanics of an overloaded ventricle. In diastole, CPAP increases pericardial pressure, decreases transmural pressure, and decreases afterload.

In this case, ventilatory support is needed and NPPV should be used. You will need to select an inspiratory pressure (IPAP) and expiratory pressure (EPAP). These correlate with pressures we are familiar with in invasive mechanical ventilation:

EPAP = PEEP

IPAP-EPAP = PS

Consider starting at 10 cm H20/5 cm H20.

**Thirty minutes later, you repeat an arterial blood gas: 7.26/67/70/31. What now?**

Go see the patient! Is the patient synchronous with the machine? Is there an air leak from the mask? If Mr. T looks comfortable, he is improving, but needs additional ventilatory support. Try increasing the IPAP (to 12 or 14, if tolerated) to achieve this.

**What evidence supports the use of NPPV in hypercarbic respiratory failure due to COPD?**

Lots of good evidence for use of NPPV in this situation. Cochrane review in 2004 by Ram and colleagues^2^ demonstrated:

- NIV decreased mortality (11 versus 21 percent), intubation rate (16 versus 33 percent), and treatment failure (20 versus 42 percent).
- Hospital length of stay and complications related to treatment were also reduced by NIV.

**In what other clinical situations may NIV be beneficial?**

1. Cardiogenic pulmonary edema: There is high-quality evidence that NIV reduces the need for intubation and improves respiratory parameters, particularly in patients with hypercarbia. There is conflicting evidence regarding mortality.

2. Hypoxemic respiratory failure: There is conflicting evidence in this population, most likely due to differences in the causes of respiratory failure.

3. Asthma: Commonly used in severe exacerbations without significant supporting evidence. There is a low failure rate.

3. Post-extubation: May be beneficial in preventing recurrent respiratory failure if initiated early.

4. Immunocompromised: There is conflicting data – multiple small studies in immunocompromised patients with acute hypoxemic respiratory failure report that compared with mechanical ventilation or low-flow oxygen, NIV decreases mortality, intubation rate, and length of stay in the ICU. Other studies have demonstrated no benefit or harm.

References

1. Çoruh B, Kritek PA. A Case-Based Critical Care Curriculum for Resident Physicians. *MedEdPORTAL.* 2012;8. *Adapted by the authors with permission.*

2. Leuppi JD, et al. Short-term vs conventional glucocorticoid therapy in acute exacerbations of chronic obstructive pulmonary disease: the REDUCE randomized clinical trial. *JAMA*. 2013. 309(21):2223-2231.

3. Ram FS, Picot J, Lightowler J, Wedzicha JA. Non-invasive positive pressure ventilation for treatment of respiratory failure due to exacerbations of chronic obstructive pulmonary disease. Cochrane Database Syst Rev 2004; :CD004104.

**5. Mechanical Ventilation**

Adapted from Çoruh B and Kritek PA.

Learning Objectives

1. List the indications for invasive mechanical ventilation.
2. Describe and compare basic ventilator modes.
3. Describe how to evaluate peak and static pressures using the concepts of resistance and compliance.
4. Discuss how mechanical ventilation disrupts normal physiology and subsequent adverse events that may occur.

Case

The MICU team is called to the ED to evaluate Ms. N, a 72-year-old woman with advanced dementia, HTN, and atrial fibrillation who was sent in from her nursing home with fever and tachypnea. She is minimally communicative at baseline and is unable to provide further history. Her nursing home has provided an advanced directive which indicates that she is full code.

On exam, temperature is 39.8˚C, BP 100/72, HR 112, RR 30, O2 sat 90% on a non-rebreather mask. Height 64 inches, weight 102 lbs. Ms. N is frail and appears tachypneic and with use of accessory muscles of respiration. Neck veins are flat. Heart is tachycardic and irregularly irregular. She has left basilar crackles. Abdomen is soft and nontender. No peripheral edema.

ABG: 7.45/30/80/22

Labs: Na 130, K 3.2, Cl 96, HCO3 21, BUN 32, Cr 1.3, glucose 89

WBC 16,000 with left shift, Hct 32%, platelets 332,000

CXR reveals a left basilar opacity, mild cardiomegaly, and no pleural effusions.

Questions

**What are indications for mechanical ventilation? Should Ms. N be intubated and mechanical ventilation initiated?**

Indications for intubation and mechanical ventilation include:

- Improve oxygenation – hypoxic respiratory failure (no specific cutoff for PaO2)
- Improve ventilation – hypercarbic respiratory failure (no specific cutoff for PCO2)
- Reduce work of breathing (RR > 35/min)
- Airway protection (examples: encephalopathy, upper GI bleed, hemoptysis)
- Reduce systemic or myocardial oxygen demand (examples: shock, severe acidosis)
- Stabilize the chest wall (examples: trauma, flail chest)

**What are the basic ventilator modes? What initial ventilator settings will you choose for Ms. N?**

| **Mode** | **Variables** | **Advantages** | **Disadvantages** |
| --- | --- | --- | --- |
| Assist Mechanical Ventilation (AMV) or Assist-Control (A/C) | V_T_ = independent  RR = independent  PEEP = independent  FiO_2_ = independent  PIP = dependent | *Full support  *Decrease WOB  *Ensures minute ventilation  *Can measure lung mechanics | *Breath stacking and auto-PEEP  *Fixed flow rate/pattern |
| Pressure Control Ventilation (PCV) | V_T_ = dependent  RR = independent  PEEP = independent  FiO_2_ = independent  PIP = independent | *Full support  *Variable flor rate/pattern (more comfortable)  *Less barotrauma  *Increase mean airway pressure | *Minute ventilation not guaranteed  *V_T_ varies with changes in system compliance |
| Pressure Support Ventilation (PSV) | V_T_ = dependent  RR = dependent  PEEP = independent  FiO_2_ = independent  PIP = independent | *Patient comfort  *Assess readiness to extubate  *Weaning mode | *Requires respiratory drive to be intact |

There is no particular mode that is associated with a mortality benefit for the case of Ms. N. Our institution uses assist-control ventilation most commonly.

Settings:

- V_T_ (tidal volume): 6-8 mL/kg of predicted body weight (PBW)
  - Men: [(height in inches – 60) X 2.3] + 50
  - Women: [(height in inches – 60) X 2.3] + 45
- Rate: depends on the patient’s minute ventilation needs (normal 6-8 L/min)
- FiO_2_: start at 100%, wean based on oxygen saturation
- PEEP: set at 5 cmH_2_O and increase as needed
- PIP: typically set <20 cmH_2_O

One possibility for Ms. N: A/C at a rate of 16, VT 450 mL, FiO2 1.0, PEEP 5.

**Ms. N remains mechanically ventilated for the next several days with increasing oxygen requirements. On hospital day 3, you are paged because the peak pressure alarm is going off on her ventilator; the PIP is 42 cm H2O. What other information do you want and how will you sort out what is going on?**

Go see the patient! You can get a sense of what is going on just by watching the patient breathe:

- Is she dyssynchronous with the ventilator?
- Listen to the patient’s lungs: do you hear air movement bilaterally?
- At this point, you can ask the respiratory therapist to perform an inspiratory hold to determine the plateau (also called static) pressure. The Pplat is 36 cm H2O.

*Compliance*: change volume / change pressure

V_T_  🡪 Normal > 100 ml/cmH_2_O

Pplat – PEEP (50-60 mL/cmH2O on vent)

*Resistance*: change pressure / change volume

PIP – Pplat 🡪 Normal < 10 cmH_2_O/L/s

flow

**On exam, Ms. N has coarse breath sounds, but they are audible bilaterally. She appears to be breathing synchronously with the ventilator. What is her resistance and compliance and how will you evaluate further?**

Resistance = PIP – Pplat = 42 – 36 = 6 cm H20/L/s

flow 60 L/min

Compliance = V_T_ = 450 = 14.5 mL/cm H2O

Pplat – PEEP 36-5

She has a normal resistance, but markedly low compliance. Next step is to obtain a CXR…which now reveals diffuse bilateral opacities. You suspect ARDS as the cause of her worsening compliance.

References

1. Çoruh B, Kritek PA. A Case-Based Critical Care Curriculum for Resident Physicians. *MedEdPORTAL.* 2012;8. *Adapted by the authors with permission.*

2. <http://courses.washington.edu/med610/mechanicalventilation/mv_primer.html>

3. Clemons J and Kearns MT. Invasive Mechanical Ventilation. Hosp Med Clin 5 (2016) 17–29.

**6. Diabetic Ketoacidosis and the Hyperosmolar Hyperglycemic State**

Adapted from Çoruh B and Kritek PA.

Learning Objectives

1. Explain the pathophysiology of DKA and HHS.
2. Identify common precipitating factors of DKA and HHS.
3. Describe the management of DKA and HHS including the use of fluids, insulin, and electrolytes.
4. Describe complications that can occur in the management of DKA and HHS.

Case

The MICU team is called to the ED to evaluate Mr. P, a 33-year-old man with a history of poorly-controlled type 1 DM. He was brought in by the paramedics for abdominal pain and was noted to have a respiratory rate in the 30-40s and blood glucose of 357 in the field.

On your exam, temperature is 36.4˚C, BP is 104/66, HR 132, RR 32, and O2 sat 100% on 2 L/min oxygen via nasal cannula. Weight is 72 kg. Mr. P is thin, with dry mucous membranes. His respiratory pattern is notable for deep, rapid breathing. Lungs are clear; heart is tachycardic, but regular. Abdomen is soft and nondistended with mild tenderness throughout. No peripheral edema. Neurologically, he is alert, but distracted by abdominal pain. He appears mildly confused, but is following commands.

ABG: 6.89/14/132/3 on 2 L/min oxygen via nasal cannula

Labs reveal Na 134, K 5.2, Cl 92, HCO3 <5, BUN 18, Cr 1.7, glucose 361

WBC 26,000, Hct 36%, platelets 371,000

Lactate 3.9, U/A with 4+ ketones, serum ketones positive at 1:16

Questions

**Starting with the ABC’s, do you think we should intubate the patient?**

No. Although the patient is markedly tachypneic, his Kussmaul respirations are an attempt at compensation for profound acidosis, which should improve rapidly with fluids and insulin. He is protecting his airway and oxygenating well and we often cannot do as good a job increasing minute ventilation as patients can on their own. If he were to get intubated, keep this in mind when setting your RR and VT.

**What is the pathophysiology of Mr. P’s condition and your initial treatment?**

The patient has DKA as evidenced by hyperglycemia, gap metabolic acidosis, and ketonemia. Lack of insulin and elevated counter-regulatory hormones (glucagon, catecholamines, cortisol, growth hormone) lead to altered glucose production, increased lipolysis, and production of ketones. Blood glucose is often only modestly elevated. Hyperglycemia is due to increased gluconeogenesis and glycogenolysis and impaired glucose utilization in the tissues. Hyperglycemia causes osmotic diuresis, resulting in hypovolemia and decreasing GFR, thus worsening hyperglycemia.

Treatment begins with fluid resuscitation (2-3 L of isotonic fluids), followed by a loading dose of 0.1 units/kg IV regular insulin followed by a maintenance dosage of 0.1 units/kg/hr OR an insulin infusion at 0.15 units/kg/hr without a bolus. Milder cases of DKA may be managed with subcutaneous short-acting insulin analogs (i.e. lispro or aspart).

**What is the pathophysiology of HHS and how does your initial treatment differ from the treatment of DKA?**

The hallmarks of HHS are profound hypovolemia (more severe than is seen in DKA) and marked hyperglycemia (usually >600 mg/dL) with mild or no ketosis. HHS usually occurs in patients with type 2 DM who have insulin levels that are sufficient to prevent lipolysis and subsequent ketonemia. HHS results in massive glucosuric diuresis resulting in hypovolemia. The most important initial treatment is very aggressive fluid resuscitation (usually ~9L in first 48 hours). Insulin therapy is much less important than volume resuscitation, and patients can be initially managed with saline infusion alone. Initial insulin dosing is the same as for DKA, but patients with HHS often have underlying insulin resistance and may require higher insulin infusion rates to achieve blood glucose targets (glucose 140-180). HHS may result in a very high plasma osmolarity and neurologic abnormalities as a result.

**What are common precipitants of DKA and HHS and how will you work up Mr. P?**

- Infection [thorough physical exam, CXR, blood and urine cultures]
- Insulin, lack of [history]
- Ischemia/infarction: MI, stroke, pulmonary embolism, mesenteric ischemia [EKG, cardiac enzymes, lactate]
- Intra-abdominal process: pancreatitis, GI bleeding [LFTs, pancreatic enzymes]
- *Note that modestly increased serum amylase and lipase levels are seen in 16-25% of
- DKA cases and do not always signify acute pancreatitis
- Iatrogenic: drugs such as glucocorticoids, atypical antipsychotics, thiazides, beta blockers, phenytoin may contribute to HHS, but seldom (if ever) are the sole cause of DKA/HHS [history]
- Ingestion: alcohol (more likely to cause HHS), cocaine [blood alcohol level, urine toxicology]

*Consider SGLT2 inhibitors as well when reviewing the patient’s medication list – this usually does not have an associated anion gap, however.

**While Mr. P is getting his initial treatment, how do you correct his sodium for his hyperglycemia and how do you calculate his serum osmolarity (Sosm)?**

Corrected serum Na = [(BG - 100)/100) x 1.6] + measured serum Na [361-100/100 x 1.6] + 134

= 138

Sosm: [2 x Na (mEq/L)] + BUN/2.8 + glucose (mg/dL)/18 = (2 x 134) + 18/2.8 + 361/18

= 294 mOsm/kg

**How will you continue to treat him in regards to fluids, insulin, and electrolytes?**

At our hospital, this is protocolized within the DKA order set, which we can review. The general concepts are explained below.

Fluids: After adequate resuscitation with isotonic fluid, evaluate corrected serum Na and

continue IVFs at 5-15 mL/kg/h:

- If corrected serum Na is normal or high, change IVFs to ½ NS
- If corrected serum Na is low, continue with 0.9% NaCl

Once serum glucose reaches 250 mg/dl, change fluids to D51/2NS:

Insulin:

Insulin gtt at 0.1 units/kg/hr until anion gap closes; goal is to decrease BG by < 100 mg/dL/hr

Electrolytes:

- Potassium: Most patients in DKA will be admitted with normal or high potassium levels, but remember that they are total body potassium depleted and that this relatively high K is due to acidemia, insulin deficiency, and hypertonicity. Begin KCl supplementation when K < 5.0 mEq/L.
- Phosphate: Total body phosphate deficiency is also present in DKA, but its clinical relevance and benefits of replacement are uncertain. Replace phosphate only if PO4 < 1.0 mg/dL and monitor for hypocalcemia with phosphate repletion.
- Bicarbonate: Repletion controversial. No benefit seen in controlled studies in patients with pH 6.9-7.1, but most experts recommend treatment with bicarbonate if pH < 7.0

**What complications do you want to monitor for during your treatment?**

- Hypoglycemia
- Hypokalemia
- Hyperchloremic non-gap acidosis due to overly aggressive resuscitation with normal saline
- Cardiogenic pulmonary edema
- Thromboembolism due to enhanced hypercoagulable state (don’t forget venous thromboembolism prophylaxis)
- Cerebral edema (much more common in pediatric population): thought to be due to osmotic factors, hypocapnia, hypovolemia (latter two causing cerebral ischemia). Prevention is with slow correction of hyperglycemia. Treatment is mannitol and mechanical ventilation.

**Eight hours after admission, Mr. P’s glucose is 160, his abdominal pain has resolved, and he is tolerating a clear liquid diet. He has been on a steady infusion of 1.5 units of insulin per hour for several hours. How will you convert him to subcutaneous insulin?**

Options include:

A. Resume pre-hospitalization insulin regimen

B. Weight-based dosing. For patients with type 1 DM, this is 0.5-0.8 units/kg/day.

C. Convert based on in-hospital insulin requirements

- Calculate 24 hour daily IV insulin requirement based on hourly average. For Mr. P, this is 36 units/day.
- Convert 70% of the daily IV insulin requirement to subcutaneous insulin (approximately 25 units SC insulin/day). Note that this is a conservative estimate.
- Give ½ of subcutaneous insulin as basal (13 units daily of glargine or 6 units BID of NPH) and the remaining ½ as prandial (13/3 = 4 units regular insulin with meals)
- Don’t forget to overlap IV insulin and subcutaneous insulin for 1-2 hours

References

1. Çoruh B, Kritek PA. A Case-Based Critical Care Curriculum for Resident Physicians. *MedEdPORTAL.* 2012;8. *Adapted by the authors with permission.*

2. Umpierrez GE, et al. Diabetic Ketoacidosis and Hyperglycemic Hyperosmolar Syndrome. Diabetes Spectrum. 2002;15:28-36.

**7. Acute Respiratory Distress Syndrome and Refractory Hypoxemia Salvage Therapies**

Adapted from Çoruh B and Kritek PA.

Learning Objectives

1. Systematically evaluate the patient with hypoxemia.
2. Define ARDS and list common precipitants.
3. Discuss ICU treatment strategies for ARDS including low-tidal volume ventilation, PEEP, and fluid management.
4. Describe salvage therapies for refractory hypoxemia.

Case

You are called to evaluate Ms. D, a 56-year-old woman on the Neurosurgical floor who is post-operative day #5 from a craniectomy for a left frontal mass. Her post-operative course was complicated by witnessed aspiration on POD #2 for which she was started on ampicillin/sulbactam. Chest radiograph at that time revealed a right middle lobe opacity consistent with aspiration. Her oxygen requirements have been slowly increasing since that time (from 2 L/min to 5 L/min O2 via nasal cannula) and this evening she desaturated to 82% on 5 L/min O2 and was placed on a non-rebreather. She complains of dyspnea and a dry cough.

On exam, temperature is 36.8˚C, BP 132/72, HR 114, RR 28 and O2 sat is 92% on a non-rebreather mask. The craniectomy appears to be healing well. JVP is not elevated. Heart is tachycardic, but regular. Lungs have coarse crackles bilaterally. Abdomen is soft and nondistended. Trace pitting edema is present bilaterally. Neurologic exam is stable over the last several days.

ABG: 7.30/48/57/22 on a non-rebreather

CXR demonstrates diffuse bilateral opacities, no pneumothorax

Recent pre-operative TTE with EF 67%, no regional wall motion abnormalities, no evidence of diastolic dysfunction

Questions

**What are the five causes of hypoxemia and what do you think is the cause of Ms. D’s hypoxemia?**

1. Low FiO2 (normal A-a)

2. Hypoventilation (normal A-a)

3. V/Q mismatch

4. Shunt

5. Diffusion abnormality

The A-a O2 difference can be calculated by:

PAO2 = [FiO2 x (Patm – 47 mm Hg)] – (PaCO2/0.8)

A-a O2 difference = PAO2 – PaO2

In Denver, where the barometric pressure is typically 630-640mmHg instead of 760mmHg, we would calculate her A-a gradient to be 476mmHg. A normal A-a O2 difference is (age/4) + 4, or <18 mmHg for Ms. D. The most likely etiologies for her hypoxemia are V/Q mismatch and shunt, with the latter being more likely due to lack of correction with 100% FiO2. In the absence of known cardiac causes of R->L shunt, this raises the question of pulmonary shunt physiology.

**How is acute respiratory distress syndrome (ARDS) defined and what are common precipitants? Do you think Ms. D has ARDS?**

Using the Berlin definition, the criteria are as follows^1^:

1. Onset within 7 days after a known clinical insult or new or worsening respiratory symptoms

2. Bilateral radiographic opacities “consistent with pulmonary edema” although non-cardiogenic

3. Categorization by severity using PaO2:FiO2 ratio (P/F ratio)

MILD: 201-300 mmHg; mortality 27%

MODERATE: 101-200 mmHg; mortality 32%

SEVERE: ≤ 100 mmHg; mortality 45%

4. Minimum PEEP setting (or CPAP) of 5 cmH2O

Common precipitants:

- Sepsis
- Aspiration
- Pneumonia
- Pancreatitis
- Burns
- Trauma
- Blood transfusions
- Near drowning
- Drug overdose

Ms. D fulfills all the criteria for ARDS and has a recent aspiration event that may have been a precipitating factor.

**Ms. D is intubated for hypoxemic respiratory failure and promptly transferred to the ICU. Initial ventilator settings are AC at a rate of 18, V_T_ 8 mL/kg, FiO2 1.0, PEEP 5. What do you want to monitor on the ventilator?**

Peak inspiratory pressure (PIP) and plateau pressure (Pplat), the latter of which is assessed by performing an end-inspiratory hold.

**Ms. D’s PIP is 45 cm H2O and Pplat is 38 cm H20. What do these pressures tell you about her respiratory mechanics?**

The peak inspiratory pressure is high, which may be due to ventilator dyssynchrony, increased resistance, or decreased compliance. We can calculate resistance and compliance:

Resistance R = ΔP = PIP-Pplat Normal = <10 cm H2O/L/s

flow flow (usually 60 L/min)

Compliance C = ΔV = VT Normal = >100 mL/cm H2O

ΔP Pplat-PEEP (50-60 mL/cm H2O when on ventilator)

R = PIP-Pplat = 45-38 = 7 cm H20/L/s

flow 60 L/min

C = V_T_ = 500 = 15 mL/cm H2O

Pplat-PEEP 38-5

Resistance is normal, but compliance is very low. This is the expected finding in ARDS, where cytokine release causes capillary leak and non-cardiogenic pulmonary edema, resulting in very stiff lungs.

**Looking back at Ms. D’s initial blood gas (7.30/48/57/22), how do you explain her hypercarbia?**

Alveolar hypoventilation may be due to decreased minute ventilation or increase in dead space

(areas of lung that are ventilated, but not perfused). Increased dead space can sometimes be seen in ARDS and is thought to be due to microthrombi in pulmonary capillaries.

**What would you like to do with Ms. D’s ventilator settings at this point? Why?**

Given the presence of ARDS, you want to place the patient on “lung protective ventilation” or low tidal volume ventilation. Goal is to achieve a VT of 6 mL/kg or less to maintain Pplat < 30.

Data to support this ventilator strategy comes from the ARDSNet low tidal volume ventilation strategy^3-4^.

Recall that volume of ventilation depends on PBW (based on gender and height):

Men: [(height in inches – 60) X 2.3] + 50

Women: [(height in inches – 60) X 2.3] + 45

In the ARMA trial^5^, 861 patients were randomized to receive high (12 mL/kg) or low (6 mL/kg) tidal volume ventilation with a goal Pplat ≤ 30 cm H20 in the low tidal ventilation group. Goal PaO2 was 55-80 mm Hg (SpO2 88-95%) via combination of FiO2 and PEEP.

- Trial stopped early due to evidence of mortality benefit in low tidal volume group
- Mortality: 31.0% (low VT) vs. 39.8% (high VT), p=0.007
- Off ventilator by day 28: 65.7%(low VT) vs. 55.0% (high VT), p=<0.001
- No difference in barotrauma between groups
- Note that the low tidal volume and plateau pressure limitation are the key factors in decreasing mortality in ARDS. No study has shown a mortality benefit from any other aspect of ventilator management (e.g., mode of ventilation).

**Ms. D is now on AC at a rate of 28, VT 6 mL/kg, FiO2 0.7, and PEEP 12 (using the “low PEEP” ladder; refer to ARDSNet Mechanical Ventilation Protocol Summary^3^) and your next ABG is 7.26/58/67/23. What do you want to do with the ventilator now?**

No changes are necessary. The PaO2 is within the goal of 55-80 mm Hg. Permissive hypercapnia is part of the low tidal volume ventilation strategy and allows for pH ≥ 7.15. Alveolar hypoventilation occurs as a result of the decrease in minute ventilation that is employed to decrease alveolar overdistention.

**You recall learning that PEEP is helpful in diffuse lung processes. Would a further increase in PEEP help in this situation?**

Brower et al. studied this in the ALVEOLI trial. 549 patients with ALI/ARDS were randomized to lower or higher PEEP^6^.

- In an embedded trial within the study, 80 patients received a recruitment maneuver (35-40 cm H2O CPAP for 30 seconds); this was later stopped due to small and transient increase in PaO2.
- On average, low PEEP was 8 cm H20 and high PEEP was 13 cm H20.
- Protocol was changed during the study to allow for higher levels of PEEP in the high PEEP arm.
- No significant difference between low and high PEEP groups in terms of death and ventilator-free days.

*Note that some patients benefit from higher PEEP and that care should be individualized to the patient.

**An hour later, you get a call that her oxygen saturation has dropped to 80%. No changes have been made to the ventilator in the interim. Someone suggests performing a recruitment maneuver. What is your response? What other therapies could you consider at this point?**

Recruitment maneuvers involve the brief application of high continuous positive airway pressure (35-40 cmH2O) for 40-60 seconds to open collapsed alveoli. The ART trial randomized 1013 patients to incremental application of higher PEEP (25 cm H_2_O with an inspiratory driving pressure of 15 cm H_2_O above the PEEP, uptitrated to maximal of 35 cm H_2_O) with return to volume control and down-titration every 3 minutes or low PEEP guided by FiO2. Death at 28 days was higher in the experimental group with NNH of 17 as well as increased pneumothorax and barotrauma. There is insufficient evidence to support the routine use of recruitment measures and they can cause hypotension and desaturation.

Paralysis or neuromuscular blockade is reserved for patients with ARDS and severe, refractory, life-threatening hypoxemia to promote ventilator synchrony. They may assist in lung-protective ventilation by preventing spontaneous respiration, decreasing work of breathing, and preventing injurious overinflation. Cisatracurium is the most commonly used agent (although earlier studies used vecuronium and pancuronium).

- One of the first major randomized controlled trials (ACURASYS^8^) demonstrated that paralysis with cisatracurium for 48 hours (versus placebo) in 340 intubated patients with early severe ARDS improves 90 day survival and increases ventilator-free days.
- Undesirable effects associated with neuromuscular blockade include prolonged neuromuscular weakness and development of critical illness polymyoneuropathy.

Prone positioning improves oxygenation in the majority of patients with ARDS by altering the mechanics and physiology of gas exchange.

- There is greater expansion of ventral alveoli when the patient is proned. Excess lung weight in the supine position worsens the normal difference between the ventral and dorsal transpulmonary pressures.
- Ventilation becomes more homogenous in the prone position, decreasing ventral alveolar over-inflation and dorsal alveolar collapse.
- Reduced lung compression by the heart and diaphragm.
- Improved lung perfusion, especially of the dependent portions of the lung.

*One large RCT (PROSEVA)^9^ and several meta-analyses suggest improved survival from prone ventilation (especially in patients with severe ARDS when there is no improvement despite above ventilatory strategies).

*Proning is typically performed after 12-24 hours of initial stabilization. Optimal duration is unknown, with studies demonstrating single sessions 6-8 hours per day or prolonged sessions up to 20 hours per day; there is no difference in benefit between the two.

Inspiratory to expiratory ratios can be modified to increase the inspiratory component while sacrificing the expiratory component to improve alveolar recruitment and promote homogenous ventilation. This is typically done on a case-by-case basis with no evidence supporting definitive use of this strategy.

Switching modes of ventilation to increase mean airway pressure (especially if recruitment helped:

1. Pressure control ventilation with a long inspiratory time.
2. Airway pressure release ventilation (APRV) or high frequency oscillatory ventilation (HFOV) was used with more frequency during the 2009 H1N1 influenza pandemic to manage ARDS with refractory hypoxemia, however a randomized controlled trial demonstrated higher risk of mortality with this.

*The above will decrease preload, so be aware this can also lead to decreased cardiac output and decreased oxygen delivery.

Open-lung ventilation combines low tidal volume ventilation and a recruitment maneuver followed by applied titrated PEEP to achieve higher alveolar recruitment. There is no accepted protocol and no evidence that there is conclusive benefit. Given possible harm with excess hypercapnia and higher 28-day mortality, we do not use this routinely.

Esophageal pressure monitoring can provide an estimate of pleural pressure and therefore can be used to calculate the transpulmonary pressure. This then could theoretically be adjusted to titrate PEEP and reduce alveolar collapse, while also limiting alveolar overdistention. This strategy does not have any proven benefit for use on a routine basis.

P_transpulmonary_ = P_in_ – P_out_

Pt_ranspulmonary_ = P_alveolar_ – P_pleural_

Pleural pressure may be very high in the setting of a distended abdomen or severe anasarca. The transpulmonary pressure therefore may be negative at end-expiration and result in worsening atelectasis. This can therefore guide level of PEEP with the goal of being slightly positive (1-2cm H2O).

Extracorporeal membrane oxygenation (ECMO) may be indicated in very select severe cases (P/F<70). The EOLIA trial^10^ randomized patients with severe ARDS to early or late ECMO, demonstrating improved oxygenation, more days free of renal failure, and less ischemic stroke. The final difference in 60-day mortality was not significant. This is used as a last resort therapy after conventional methods have been unsuccessful.

**What additional therapies have been demonstrated to be effective or ineffective in ARDS?**

Despite a multitude of randomized controlled trials, no definitive drug therapy has been identified to improve survival in ARDS. Thromboxane synthetase inhibitors, inhaled nitric oxide, corticosteroids, surfactant, *N*-acetylcysteine, beta-agonists, statins, inhaled prostacyclin, liquid ventilation, and activated protein C all have no definitive benefit. The only proven therapies that definitively improve survival are low tidal volume ventilation and prone positioning.

The Fluids and Catheters Treatment Trial (FACTT)^11^ demonstrated that a conservative fluid strategy (based on CVP, goal<4) improves lung function, decreases ventilator days, and reduces ICU days compared to a liberal strategy. After randomizing 1000 patients to one of these strategies and treatment with diuretics (following hemodynamic stability, discontinuation of vasopressors) for a net even fluid strategy, there was no difference between mortality of the two groups at 60 days.

**Ms. D improves slowly over the course of the next week and is successfully extubated on hospital day 11.**

References

1. Çoruh B, Kritek PA. A Case-Based Critical Care Curriculum for Resident Physicians. *MedEdPORTAL.* 2012;8. *Adapted by the authors with permission.*

2. Thompson T, Chambers RC, and Liu KD. Acute respiratory distress syndrome. N Engl J Med 2017;377:562-72.

3. Acute Respiratory Distress Syndrome: The Berlin Definition. JAMA. 2012;307(23):2526-2533.

4. The Acute Respiratory Distress Syndrome Network. Ventilation with lower tidal volumes as compared with traditional tidal volumes for acute lung injury and the acute respiratory distress syndrome. N Engl J Med. 2000;342:1301-1308.

5. NIH NHLBI ARDS Clinical Network Mechanical Ventilation Protocol Summary: <http://www.ardsnet.org/system/files/Ventilator%20Protocol%20Card.pdf>

6. Brower RG, et al. Ventilation With Lower Tidal Volumes As Compared With Traditional Tidal Volumes For Acute Lung Injury And The Acute Respiratory Distress Syndrome. The New England Journal of Medicine. 2000. 342(18):1301-1308.

7. Brower RG, et al. Higher vs. lower positive end-expiratory pressures in patients with the acute respiratory distress syndrome. N Engl J Med. 2004;351(4):327-3.

8. Cavalcanti AB, et al. JAMA. 2017 Oct 10;318(14):1335-1345.

9. Papazian L, et al. Neuromuscular blockers in early acute respiratory distress syndrome. The New England Journal of Medicine. 2010. 363(12):1107-1116.

10. Guérin C, et al. Prone positioning in severe acute respiratory distress syndrome. The New England Journal of Medicine. 2013. 368(23):2159-2168.

11. Combes A, et al. Extracorporeal membrane oxygenation for severe Acute respiratory distress syndrome. The New England Journal of Medicine. 2018. 378(21):1965-1975.

12. Wiedemann HP, et al. Comparison of two fluid-management strategies in acute lung injury. The New England Journal of Medicine. 2006. 354(24):2564-75.

**8. Acute Liver Failure**

Adapted from Çoruh B and Kritek PA.

Learning Objectives

1. Define acute liver failure and identify its etiologies.
2. List the complications of acute liver failure.
3. Describe the ICU management of the complications of acute liver failure.

Case

The MICU team is called to the ED to admit Mr. S, a 29-year-old man with a history of depression who presents to the ED with nausea, vomiting, anorexia, and malaise x 3 days and was found to have markedly elevated aminotransferases and INR. He endorses a recent break-up with his girlfriend several days ago which led him to drink more alcohol than usual but denies other ingestions.

On exam, BP is 107/61, HR 122, RR 24, and O2 sat 100% on ambient air. Mr. S is somnolent, but arousable, with flat affect and irritability. He is disoriented with slurred speech and asterixis is present. Heart is tachycardic but regular; lungs are clear. Abdomen is soft and non-distended with mild tenderness to palpation in the epigastrium and RUQ. No stigmata of chronic liver disease. Skin is notable for jaundice, no rashes.

Labs reveal Na 133, K 5.2, Cl 85, HCO3 13, BUN 29, Cr 3.6, glucose 108

WBC 19,000, Hct 38%, platelets 371,000

AST 6000, ALT 6600, ALP 251, tbili 6.6, INR 9.1, lipase 21

ABG: 7.28/32/90/14 on ambient air, lactate 6.2

Questions

**How is acute liver failure defined? What is on your differential diagnosis?**

Definition: Severe liver injury with encephalopathy and impaired synthetic function (INR>1.5) without pre-existing disease, typically with onset < 26 weeks (to distinguish from chronic).

Differential diagnosis:

1. Direct cellular injury

Drugs (acetaminophen most commonly, antibiotics, antiepileptics, isoniazid)

Toxins (amanita mushrooms, carbon tetrachloride)

Viruses (HAV, HBV, HCV, HEV, VZV, HSV, CMV, EBV, adenovirus)

Autoimmune hepatitis (ANA, ASMA, anti-liver/kidney microsomal antibodies-ALKM)

2. Autoimmune: autoimmune hepatitis

3. Metabolic:

Wilson’s disease

HELLP

Acute fatty liver of pregnancy

3. Vascular:

Hypoperfusion from cardiac/pericardial disease, shock, sepsis

Outflow obstruction, Hepatic vein thrombosis (Budd Chiari), veno-occlusive disease

Compression on arterial or venous supply by mass/malignancy

**How would you grade the patient’s encephalopathy? Do you think he should be intubated?**

Grade 1: Disordered sleep/wake cycles, mild confusion, behavior changes, lack of awareness, subtle asterixis

Grade 2: Lethargy/apathy, moderate confusion, obvious asterixis

Grade 3: Somnolent or stuporous, significant confusion, incoherent speech, arousable with stimulation, may have clonus

Grade 4: Coma, loss of reflexes, unresponsive to painful stimuli

The patient appears to have grade 3 encephalopathy and should be intubated; patients should be intubated for grade 3-4 encephalopathy to prevent aspiration.

**Initial laboratory evaluation reveals an acetaminophen level of 73. How will you proceed with management?**

Toxic levels of acetaminophen increase the level of toxic metabolite NAPQI, which injures critical proteins of hepatocytes. Hepatotoxicity is actually lower in patients who acutely drink alcohol as this competitively utilizes CYP2E1 and decreases the amount of NAPQI produced. Chronic alcohol ingestion enhances CYP2E1 activity, but there is no evidence of increased hepatotoxicity associated.

IV N-acetylcysteine (NAC) should be instituted to replenish hepatic glutathione stores and with guidance from the Rumack-Matthew nomogram, may be indicated in acute acetaminophen ingestion alongside risk for hepatotoxicity. The 20-hour IV protocol is used most often (there are also 12-hour and 72-hour protocols and oral protocols), providing a total of 300 mg/kg. It should be continued until there is consistent evidence of hepatic recovery (resolution of encephalopathy, INR <2, ALT<50% of peak, or three serial measurements of ALT<1000 IU/L, and the acetaminophen concentration is undetectable). No randomized controlled studies evaluating efficacy but known for decreasing mortality and improving hepatic and cerebral function even when administered late.

One placebo-controlled trial^3^ of patients with acute liver failure due to causes other than acetaminophen (drug-induced, autoimmune, Hepatitis B virus predominantly) found higher transplant-free survival (40 versus 27 percent) in patients randomized to NAC (especially in those with early hepatic encephalopathy).

**What are the complications of acute liver failure and how will you monitor/treat them?**

1. Hepatic encephalopathy: Most sensitive way to monitor is with psychometric testing (ex. serial number connection test) in early stages.

- Avoid sedatives – intubated patients should receive propofol (may not be needed in stage IV encephalopathy); avoid benzodiazepines and opiates (decrease seizure threshold).
- There is no evidence that lactulose is helpful in acute liver failure.

2. Coagulopathy: Elevated INR due to decreased factor production and thrombocytopenia (as well as abnormal platelet morphology and function; mechanism unknown).

- INR is a very sensitive marker of synthetic function, so FFP transfusion should be avoided except in the setting of active bleeding or procedures (small trial did not influence mortality and can lead to fluid overload).
- It is acceptable to give vitamin K to treat any potential contribution of vitamin K deficiency.
- Stress ulcer prophylaxis (PPI or H2B) indicated.

3. Acute kidney injury: Seen in 40-50% of acute liver failure (70% of acetaminophen-induced acute liver failure).

- Commonly due to pre-renal state or acute tubular necrosis.
- Avoid nephrotoxins and consult Nephrology early in the course.

4. Hypoglycemia: Impaired gluconeogenesis in advanced liver failure.

- Treat sustained hypoglycemia with D10 gtt.

5. Infection: Second most common cause of death after cerebral herniation.

- Cause is multifactorial from complement deficiency, reduced opsonization, dysfunctional killer cells, and WBC dysfunction.
- Common sites of infection are blood, lung, and urine and common organisms are staphylococci, streptococci, and enteric Gram-negative rods.
- Fungal infections are seen in patients with prolonged hospitalization, those on continuous veno-venous hemodialysis (CVVH), and patients receiving glucocorticoids.
- AASLD guidelines suggest daily urine, sputum, blood cultures, and CXR; data is lacking for the value of surveillance cultures and positive results may be colonization or contamination. There is no role for prophylactic antibiotics.

7. Cerebral edema: Most common cause of death.

- Mechanism not well understood but is thought to be due to altered autoregulation of cerebral blood flow, osmotic derangements in astrocytes, and altered cellular metabolism.
- Symptoms – pupillary dilation and/or abnormal pupillary response to light, irregular respirations, increased motor tone, sudden rise or drop in systemic blood pressure, bradycardia.
- Consider a head CT for any sudden changes in mental status.
- Can be monitored with intracranial pressure (ICP) monitor (placed by Neurosurgery into epidural, subdural, intraparenchymal, or intraventricular space), but risk of bleeding is high (10-20%) and patients require frequent transfusions to correct coagulopathy while monitor is in place.
- Used to tailor therapy below with goal ICP <25 mm Hg and cerebral perfusion pressure (CPP) > 50 mm Hg (CPP = MAP – ICP).

Measures to decrease ICP:

- Decrease stimulation (quiet room, minimize suctioning)

- Elevate HOB to 30 degrees

- Maintain euthermia (i.e., avoid hyperthermia)

- Maintain euvolemia (i.e., avoid hypervolemia)

- Mannitol (0.5 to 1.0 g/kg) to target serum osmolarity of 320 mOsm/kg (caution with renal failure).

- Hypertonic saline to target serum Na 145-155 mEq/L

*Note that hyperventilation to pCO2 of 25 mm Hg is only helpful in emergencies (i.e., impending herniation). Goal pCO2 should be 35-40 mm Hg.

**Is Mr. S medically a transplant candidate and what is his prognosis?**

King’s College Criteria for Transplantation for acetaminophen-related acute liver failure:

- Arterial pH <7.3 OR
- Grade 3-4 encephalopathy AND
- PT >100 s (INR > 6.5) AND
- Serum creatinine > 3.4 mg/dL

King’s College Criteria for Transplantation for NON-acetaminophen-related acute liver failure:

- PT > 100 s (INR > 6.5) OR
- Any three of the following:
  - Age <10 or >40 years
  - Etiology (non-A, non-B hepatitis, halothane hepatitis, idiosyncratic drug reaction)
  - Jaundice preceding encephalopathy by 7 days
  - PT > 50 s (INR > 3.5)
  - Serum bilirubin > 18 mg/dL

*Note one study^4^ that compared MELD with King’s College Criteria demonstrated the King’s College Criteria was more sensitive and specific (88% and 71%) compared to MELD of 32 or higher (79% and 71%) respectively

Mr. S does meet these criteria, but the ultimate decision of whether he is a transplant candidate rests with the Hepatology and Transplant teams. Frequent prohibitive factors are poor psychosocial support and alcohol/drug use within the last six months. Note that a single suicide attempt does not preclude liver transplantation.

Transplant-free survival is highest for patients with acute liver failure due to acetaminophen (~70%) and hepatitis A (~50%). Overall spontaneous recovery in acute liver failure is estimated at 40%.

References

1. Çoruh B, Kritek PA. A Case-Based Critical Care Curriculum for Resident Physicians. *MedEdPORTAL.* 2012;8. *Adapted by the authors with permission.*

2. <https://www.aasld.org/sites/default/files/guideline_documents/alfenhanced.pdf>

3. <https://en.wikipedia.org/wiki/File:Rumack_Matthew_nomogram_with_treatment_(study)_line.pdf#globalusage>

4. Lee WM, Hynan LS, Rossaro L, et al. Intravenous N-acetylcysteine improves transplant-free survival in early stage non-acetaminophen acute liver failure. Gastroenterology 2009; 137:856.

5. Parkash O, Mumtaz K, Hamid S, et al. MELD score: utility and comparison with King's College criteria in non-acetaminophen acute liver failure. J Coll Physicians Surg Pak 2012; 22:492.

**9. Acid-Base Disorders**

Adapted from Çoruh B and Kritek PA.

Learning Objectives

1. Identify primary acid-base disturbances and the expected compensatory responses.
2. Generate a differential diagnosis for the four primary acid-base disorders, including both gap and non-gap metabolic acidosis.

Cases and Questions

Overview to the Approach:

1. Based on the pH, is the patient acidemic or alkalemic?

2. Is the primary disorder metabolic or respiratory?

3. Is there compensation?

Calculations to consider:

- Anion gap (in metabolic acidosis): Na – (Cl + HCO3)
  - Normal anion gap = serum albumin x 2.5 (10 +/- 2)
- Winter’s Formula (to assess adequate respiratory compensation in metabolic acidosis):

1.5*HCO_3_ + 8 (+/- 2)

- Delta-delta: Patient anion gap – 12 / Patient bicarb – 24
  - If the anion gap difference is bigger than the bicarb difference, there is a metabolic alkalosis
  - If the anion gap difference is smaller than the bicarb difference, there is a non-gap metabolic acidosis
  - Add the difference in anion gap (patient’s anion gap – 12) to the patient’s bicarb
    - If < 22 there is a non-gap metabolic acidosis (more ACIDEMIC than expected)
    - If > 26 there is a metabolic alkalosis (more ALKALEMIC than expected)
- Osmolar gap: serum – measured; calculate serum: 2(Na) + BUN + glucose + EtOH

2.8 18 4.6

- A-a gradient: P_A_O2 – P_a_O2
  - P_A_O2 = [FiO2 x (P_atm_ – PH_2_0)] – (P_a_CO_2_/0.8) A-a O2 difference
  - Normal A-a O2 difference = (age/4) + 4
- Urine anion gap (NAGMA) = Na + K – Cl
  - Positive with low urinary ammonia (example: RTA)
  - Negative with high urinary ammonia (example: diarrhea)

CASE 1

A 19-year-old man is brought to the ED by his roommate with somnolence. The roommate notes that the patient has been taking unknown pills for the last few weeks and acting irritable. **How would you interpret the blood gas and what is the differential diagnosis based on your interpretation?**

ABG: 7.16/70/55/24 on ambient air

Na 138, Cl 105, HCO3 24

This is an acute, uncompensated primary respiratory acidosis. The A-a gradient is also low.

Differential diagnosis of respiratory acidosis:

A. Inadequate alveolar ventilation

1) Central nervous system (CNS) depression

- Medications (e.g., opiates, sedatives)
- CNS trauma/tumor/hemorrhage
- Obesity hypoventilation

2) Neuromuscular disorders

- Guillain-Barre syndrome
- Myasthenia gravis
- Myopathies
- Toxins (e.g., organophosphates)

3) Lung or chest wall defects

- Chest trauma
- Pneumothorax
- Diaphragmatic paralysis

4) Airway disorders

- COPD exacerbation
- Restrictive lung disease
- Airway obstruction
- Bronchospasm/laryngospasm

B. Overproduction of CO2 (rare and not usually a lone cause of hypercarbia)

- Catabolic states (i.e., severe infections, burns, trauma, malignant hyperthermia)
- Iatrogenic overfeeding

C. Increased intake of CO2 (e.g., rebreathing CO2-containing gas), rare

CASE 2

A 23-year-old woman with type 1 diabetes mellitus presents with confusion. She has had gastroenteritis for the last 48 hours and has not been eating much. She endorses diffuse abdominal pain, nausea, and vomiting and admits that she has not taking any insulin for the last day and a half. **How would you interpret the blood gas and what is the differential diagnosis based on your interpretation?**

ABG: 7.20/25/88/10 on ambient air

Na 130, Cl 80, HCO3 10, glucose 524

This is a high anion-gap metabolic acidosis (AG is 40). The difference in the anion gaps is (40-12) 28 and the difference in the bicarb is (24-10) 14, indicating the presence of a delta-delta gap. There a concomitant metabolic alkalosis. The A-a gradient is elevated at 31.

Differential diagnosis of anion-gap metabolic acidosis (CUTE DIMPLES):

- Cyanide
- Uremia
- Toluene
- Ethanol
- DKA (ketoacidosis), D-lactic acidosis (carbohydrates and GI bacteria)
- Isoniazid, Iron
- Methanol
- Propylene glycol, Phenformin, Paraldehyde
- Lactic acidosis
- Ethylene glycol
- Salicylates

Differential diagnosis of metabolic alkalosis:

A. GI losses of H+ (urine chloride < 25 mEq/L)

- Vomiting, NG suction
- Antacids in advanced renal failure

B. Renal losses of H+ (urine chloride > 40 mEq/L)

- Primary mineralocorticoid excess
- Loop or thiazide diuretics
- Hypercalcemia and milk-alkali syndrome

C. Alkali administration

D. Contraction alkalosis

E. Laxative abuse

CASE 3

A 38-year-old man with a history of depression and suicide attempts is brought in by medics after being found obtunded by his neighbor. **How would you interpret the blood gas and what is the differential diagnosis based on your interpretation?**

ABG: 7.46/22/98/15 on ambient air

Na 135, Cl 85, HCO3 15

Anion gap =35

ΔAG = (35-10) = 25

ΔHCO3 = (24-15) = 9… so there is a concomitant metabolic alkalosis.

A-a O2 difference = PAO2 = [FiO2 x (Patm – PH20)] – (PaCO2/0.8)

PAO2 = 123

PAO2 – PaO2 = (123 – 98) = 25

**What other information do you want?**

- BUN 14
- Glucose 104
- Lactate 1.6
- Blood alcohol level 0
- Serum salicylate level 62 mg/dL

There is a primary anion-gap metabolic acidosis with a metabolic alkalosis and respiratory alkalosis. There is also an elevated A-a gradient. Salicylate directly stimulates the respiratory center (respiratory alkalosis) and the anion gap metabolic acidosis is due to accumulation of organic acids. The metabolic alkalosis in this case is likely due to vomiting from the gastric irritant effects of the drug.

Differential diagnosis of respiratory alkalosis:

A. CNS

- Pain, anxiety
- CNS tumor/trauma/hemorrhage/infection
- Fever

B. Endocrine

- Pregnancy
- Hyperthyroidism

C. Pulmonary (mediated by hypoxemia and/or increased stretch)

- Pneumonia
- Pulmonary edema
- Pulmonary embolism
- Asthma or COPD

D. Hypoxia-mediated

- High altitude
- Right to left shunts
- Severe anemia

E. Drugs

- Progesterone
- Salicylate

F. Miscellaneous: Chronic liver disease, hepatic failure

CASE 4

A 68-year-old woman who recently completed a course of antibiotics for sinusitis presents

with diarrhea for three days. She reports severe weakness and has lost 2 kg during the course of her illness. **How would you interpret the blood gas and what is the differential diagnosis based on your interpretation?**

ABG: 7.24/18/120/6 on ambient air

Na 133, Cl 118, HCO3 5, BUN 50, Cr 3.5

Urine Na 31, urine K 12, urine Cl 60

Winter’s formula for compensation:

pCO2 = 1.5 (HCO3) + 8 ± 2 pCO2 = 1.5 (5) + 8 ± 2 = 13.5-17.5

Anion gap =10, so no need for Δ/Δ

A-a O2 difference = PAO2 = [FiO2 x (Patm – PH20)] – (PaCO2/0.8)

PAO2 = 127.5

PAO2 – PaO2 = (127.5 – 120) = 7.5

This is a non-gap metabolic acidosis with complete respiratory compensation.

Differential diagnosis of non-gap metabolic acidosis (NAGMA):

A. GI losses

- Severe diarrhea (loss of Na, K, HCO3)
- Pancreatitis (loss of HCO3 production)
- Intestinal fistula (loss of Na, K, HCO3)

B. Renal tubular acidoses (RTAs)

- Proximal (type II) RTA: loss of HCO3 due to decreased tubular secretion of H+
  - Associated with multiple myeloma and amyloidosis
- Distal (type I) RTA: decreased reabsorption of HCO3
  - Most severe of RTAs, associated with autoimmune conditions (Sjőgren’s, RA, SLE)
- Type IV RTA: decreased aldosterone secretion or effect (associated with diabetes)

C. Drugs

- Potassium-sparing diuretics
- Angiotensin converting enzyme (ACE) inhibitors
- Cyclosporine
- Trimethoprim
- Carbonic anhydrase inhibitors (e.g., acetazolamide)

**The most common causes of NAGMA are diarrhea and RTAs. How will you differentiate between the two?**

Calculate the urine anion gap (UAG):

UAG = (urine Na + urine K) – urine Cl

A normal UAG is zero or slightly positive

The normal renal response to metabolic acidosis is the excretion of NH_4_^+^, which should

increase the urinary chloride, causing a NEGATIVE urine anion gap (non-renal

causes of NAGMA).

*A neGUTive urine anion gap is seen with GUT causes (ie. diarrhea).

If the kidneys are not working properly, NH_4_^+^ (and thus chloride) excretion will be impaired, resulting in a POSITIVE urine anion gap (renal causes of NAGMA).

UAG = (31 + 12) – 60 = -17 confirming that diarrhea is the cause of the patient’s NAGMA.

CASE 5

An 18-year-old man is evaluated in the ED because of confusion, nausea, headache, and blurry vision after a camping trip. The patient’s friends note that he became ill several hours ago and are not aware of any ingestions. **How would you interpret the blood gas and what is the differential diagnosis based on your interpretation?**

ABG: 7.29/26/100/14 on room air

Na 140, K 4.0, Cl 100, HCO3 12, BUN 14, Cr 1.0, glucose 108

Anion gap =28

ΔAG = (28-10) = 18

ΔHCO3 = (24-12) = 12… so there is a concomitant metabolic alkalosis.

A-a O2 difference = PAO2 = [FiO2 x (Patm – PH20)] – (PaCO2/0.8)

PAO2 = 132

PAO2 – PaO2 = (132 – 120) = 12

**What other information do you want?**

- Serum ketones negative
- Lactate 0.7
- BAL negative
- Salicylates negative
- Measured serum osmolarity 326

Calculated osmolarity = 2[Na] + BUN + glucose + EtOH = 2(140) + 14 + 108 = 291

2.8 18 4.6

The measured serum osmolarity (326) is significantly higher than the calculated value

(291), so an osmolar gap is present.

Differential diagnosis of an osmolar gap:

- Hyperproteinemia
- Hypertriglyceridemia
- Alcohols (ethanol, ethylene glycol, methanol, isopropyl alcohol)
- Mannitol

In this case, serum testing for methanol is positive (also suspected based on history of blurry vision).

References

1. Çoruh B, Kritek PA. A Case-Based Critical Care Curriculum for Resident Physicians. *MedEdPORTAL.* 2012;8. *Adapted by the authors with permission.*

**10. Renal Replacement Therapy**

Adapted from Çoruh B and Kritek PA.

Learning Objectives

1. List the indications for renal replacement therapy.
2. Compare and contrast hemodialysis from ultrafiltration.
3. Differentiate between the different types of continuous renal replacement therapy (CRRT).
4. Describe the advantages and disadvantages of intermittent hemodialysis and CRRT.

Case

A 25-year-old Hispanic male without prior medical history presents to the ED with numbness of fingers and lethargy. He has been working in an apple orchard for the past 2 months doing backbreaking work. He has lost weight since migrating from Mexico and his clothes are not fitting well. He denies fevers, chills, or sweats, but has dyspnea on exertion and poor appetite with nausea and intermittent vomiting.

On exam his BP is 160/95 and HR 115; he is orthostatic. He appears tired but is awake and oriented. CV without rub and lungs have rhonchi bilaterally without rales. No jaundice. Abdomen is percussed and the bladder is not distended.

Labs reveal Na 132, K 7.4, Cl 101, HCO3 15, BUN 150, and creatinine 21

Hct 42%

UA with 3+ protein, RBC 3-5 and WBC negative, casts noted

The patient is given 3L of NS in the ED and an ultrasound is performed that does not reveal obstruction. On repeat labs 2 hours later, his K is 7.0, HCO3 is 10, anion gap is 17, BUN is 140, and creatinine is 19. An EKG has not changed since initial testing. He was given kayexalate when the first potassium level came back along with IV insulin, D50, and sodium bicarbonate. He has not had diarrhea and the potassium level has changed marginally.

Questions

**Does this patient require dialysis at this time?**

Possibly. The real question: will his renal function improve before he needs dialysis, and what is

causing his acute kidney injury? If he ingested high-dose ibuprofen for the past week in

the setting of volume depletion, he probably will need dialysis if there is an acute indication. If

no acute indication (such as refractory hyperkalemia), then you can try to wait for his renal

function to improve. If he has RPGN, then maybe his kidney function will take some time to get

better and dialysis will be useful in the interim.

**What are the indications for emergent dialysis?**

A. Acidosis, refractory to medical therapy

E. Electrolyte abnormalities: hyperkalemia, hypermagnesemia, and hyperphosphatemia in setting of AKI

I. Intoxications (e.g., ethylene glycol, methanol, salicylate, lithium, valproate)

O. Overload of volume refractory to medical therapy

U. Uremia; typically with associated encephalopathy, pericarditis, nausea/vomiting, severe platelet dysfunction in the setting of AKI

Notably, the AKIKI trial^1^ included 620 critically-ill patients randomized to early or delayed RRT and there was no difference in mortality between the groups (48.5% vs. 49.7%; P=0.79) with a significant increase in catheter-related bloodstream infections in the early RRT group. A post-hoc analysis demonstrated the timing of dialysis initiation demonstrated higher mortality among those with earlier versus no initiation and individuals with delayed RRT received more diuretics.

The IDEAL-ICU^2^ study more recently demonstrated that an early versus delayed initiation of RRT in 447 ICU patients with septic shock and AKI did not reduce all-cause mortality at 90 days.

**The patient is in the ICU and Nephrology has been consulted. The next morning the patient has made 50 mL of urine and the Foley catheter is removed. His creatinine is 20 and BUN is 145. The K is still > 6.5 and EKG shows peaked T waves. He has lethargy and N/V and now has an O2 sat of 90% on ambient air. Would you dialyze him at this time? If so, what type of dialysis would you perform?**

Hemodialysis (HD) or peritoneal dialysis (PD) are both options. PD requires an empty bladder and a catheter could be placed below his umbilicus with a trochar. HD modalities include intermittent HD, SLED, and CRRT.

**What is the purpose of dialysis?**

Toxin removal through diffusion or volume removal through ultrafiltration; both processes occur against a semi-permeable membrane. Diffusion is the process of utilizing concentration gradients of molecules such as potassium from a state of high concentration against one of a lower concentration. Diffusion occurs through the pores located in the fiber microtubing in the dialysis membrane. Blood flows through the hollow fibers and is separated from the dialysate fluid that bathes the membranes. The degree of diffusion depends on the concentration difference of the solute, the size of the solute, the blood flow rate, and dialysate flow rate.

- In this case the patient’s potassium is 7 and the dialysis bath would have a concentration of 0-1 mEq/L.
- The urea level would be 0 in the dialysate.

Ultrafiltration is fluid removal from positive pressure in the blood compartment and negative pressure in the dialysis chamber. This negative pressure uses cohesion in the tubing from outside roller pumps that create a force that draws sodium and water through the dialysis membrane into the tubing outside the blood tubing. In the ultrafiltrate, potassium and urea and other non-albumin bound small molecules will move, in a process called convection. The larger the dialysis membrane pores and greater the pressures, the better the ability to ultrafiltrate.

**What type of HD should this patient have?**

Given the patient’s stable blood pressure, we could perform intermittent HD for four hours with a low potassium dialysis bath and try ultrafiltration to remove volume. We would try to improve his uremia. If there was concern that the patient had underlying chronic kidney disease (CKD) and not just an AKI, we should caution attempts to lower the urea level quickly, given the risk of dysequilibrium syndrome during first dialysis treatment^3^. This is a rare entity involving the development of cerebral edema caused by water movement into the brain in the process of a reverse osmotic shift induced by removal of urea and decreased cerebral pH. Patients may develop headache, nausea, altered mentation, blurred, vision, and asterixis with progress to seizures, coma, and even death in severe cases. Risk factors include BUN > 175mg/dL, older age, pre-existing structural or organic neurologic disease (traumatic brain injury, stroke, seizure disorder), other conditions that predispose to cerebral edema, or conditions that increase blood-brain barrier permeability.

Slow low efficiency dialysis (SLED) is used to slowly remove urea and volume if one is concerned about hypotension, but his blood pressure is acceptable. CRRT will not remove the potassium as fast, therefore this would not be employed. No study has demonstrated a benefit between intermittent HD, SLED, or CRRT. It is a matter of nephrologist or hospital preference.

**Will this patient likely require long term dialysis?**

He probably has ATN and his renal function will improve. He may just need one dialysis treatment as we await improvement in renal function. If his nausea and hyperkalemia were controlled with medications, some may have even waited until he improved on his own. However, the dialysis procedure will make him feel better faster.

**Where would you place the dialysis catheter and what are its unique properties?**

A temporary catheter would be appropriate (Mahurkar catheter in our institution). They typically have two large lumens (arterial and venous) and are placed in the superior vena cava or inferior vena cava (with the femoral site being an inferior option). Compared to triple lumen catheters, HD catheters have larger lumen diameters (ranging from 8 to 13.5 French) to deliver high flow rates (Pouiselle’s law, up to 300-400 mL/minute). Tunneled catheters can be used for intermediate or long-term HD if it is unknown whether or not the AKI will be permanent. We check PT/INR, PTT, and platelets before placing the catheter and obtain consent.

**Briefly discuss the dialysis orders for CRRT and intermittent HD.**

HD requires a dialysis membrane. There are large pore sizes that remove larger molecules or smaller molecules faster. The blood flow rate is typically 300 mL/min and the dialysis flow rate is about 600 mL/min. We give patients heparin to prevent clotting when blood is extracorpeal. We write for the amount of volume to remove and we prescribe a dialysis bath. The bath is for potassium, HCO3 of 35 mEq/L, Na of 135 mEq/L, and ionized calcium bath of 1.25 mmol/L and it comes in dextrose of 100 mg/dL.

CRRT will have a slower blood flow rate of 50-200 mL/min and is slower because the dialysis flow rate is rate limiting at 2-3 liters/hour.

**What are the complications of dialysis?**

- Air emboli may occur causing pulmonary embolism, but air prevention chambers in the dialysis
- machine should prevent this.
- Dysequilibirum occurs when urea or other ammonia-like toxins are removed when a patient has had high levels for a long period of time. Once the urea in the blood is reduced, the urea in cells creates water movement into the cells and causes brain swelling manifested as headache and perhaps seizures. This is rare, but a major concern in patients with hepatic encephalopathy.
- Other concerns are changes in electrolytes if the dialysis bath is inaccurate.
- Infection is a concern related to the catheter or rarely the dialysis solution.
- Rare complications occur if chlorine in city water is not removed.

**What are the differences between the alphabet soup of CRRT, CVVH, SCUF, SLED, CVVHD, and CVVHDF?**

CRRT is continuous renal replacement therapy and an umbrella term for the modalities below. They all work better and prevent clotting when heparin is used as an anticoagulant. If this is contraindicated, regional calcium citrate is used as an anticoagulant.

| **Abbreviation** | **Name** | **Purpose** | **Special considerations** |
| --- | --- | --- | --- |
| CVVH | Continuous veno-venous hemofiltration | A form of convective dialysis; SCUF is an example of this |  |
| CVVHD | Continuous veno-venous hemodialysis | HD that is slow and continuous |  |
| CVVHDF | Continuous veno-venous hemodiafiltration | Addition of convection to HD and UF | Added clearance of molecules with solution such as NS |
| SCUF | Slow continuous ultrafiltration | Slow removal of fluid |  |
| SLED | Slow low-efficiency dialysis |  |  |

References

1. Çoruh B, Kritek PA. A Case-Based Critical Care Curriculum for Resident Physicians. *MedEdPORTAL.* 2012;8. *Adapted by the authors with permission.*

2. Gaudry S, et al. Initiation strategies for renal-replacement therapy in the intensive care unit. The New England Journal of Medicine. 2016. 375(2):122-133.

3. Barbar SD, et al. Timing of renal-replacement therapy in patients with acute kidney injury and sepsis. The New England Journal of Medicine. 2018. 379(15):1431-1442.

44. Arieff AI. Dialysis disequilibrium syndrome: current concepts on pathogenesis and prevention. Kidney Int 1994; 45:629.

**11. Upper Gastrointestinal Bleed and Blood Products**

Learning Objectives

1. List the causes of upper gastrointestinal bleed and immediate management strategies.
2. Identify unique aspects on managing upper gastrointestinal bleed in patients with cirrhosis.
3. Discuss evidence-based strategies for management of acute upper gastrointestinal bleed.
4. Assess the role of various blood products (red blood cells, cryoprecipitate, fresh frozen plasma, prothrombin complex concentrate) in management of gastrointestinal bleed.
5. Describe the role of thromboelastography (TEG) in evaluation of coagulopathy.

Case

A 47-year-old male is admitted to the ICU from the emergency department. He has a history of cirrhosis secondary to hepatitis C virus (HCV) and was seen in hepatology clinic a year ago to be evaluated for HCV therapy. He underwent an EGD at that time, which demonstrated grade II esophageal varices that were not amenable to banding. He presented today after the police found him a few blocks from the hospital vomiting bright red blood.

Vitals include T 99.2F, HR 135 bpm, BP 78/45, RR 25/min, and SpO2 82% on ambient air

He appears jaundiced and lethargic and is occasionally responding to verbal commands. He is moving all extremities spontaneously. There is blood visible in his oropharynx and telangiectasias on his chest. His peripheral pulses are thready and his extremities are cool.

Questions

**How would you like to initially manage this patient?**

- First, address the ABC’s. He is encephalopathic, not protecting his airway, and experiencing hematemesis from a likely life-threatening upper GI bleed with known esophageal varices. He needs to be intubated and resuscitated.
- He needs adequate large-bore peripheral intravenous access (ideally 18 gauge or larger) or a Cordis (introducer sheath) which ranges from 4 French to 11 French (typically 8 Fr are stocked).
- You can consider initiating the massive transfusion protocol in which you call the blood bank to request trauma blood (O negative), cryoprecipitate, and fresh frozen plasma in bulk. Caution large volume resuscitation with crystalloid and blood products. Over-resuscitation can cause harm given rapid increases in central venous pressure therefore increase the driving pressure of the portal hypertension, therefore worsening the acute bleed.
- Gastroenterology should be aware of the patient in the emergency department, but it is best to formulate a plan for when endoscopic evaluation is possible.

Several risk assessment tools can be used to evaluate the patient with UGIB. The most common is the Glasgow-Blatchford score, which utilizes multiple variables (age, Hb, BUN, SBP, HR, clinical symptoms) to indicate whether hospitalization is needed, intervention would be required, and likelihood of death from the acute bleed^1^. This patient needs ICU level care given the hemodynamic instability and concern for variceal bleed.

**Your first set of labs return:**

**Na 130 mg/dL, K 4.0, Cr 1.8 mg/dL, glucose 101 mg/dL**

**WBC 8,000 mg/dL, Hg 6.2 mg/dL, Platelets 70,000 mg/dL**

**INR 2.2**

**What therapies will you initiate for the particular disease states that could be causing this presentation?**

Your leading suspicion is that the patient has an acute UGIB secondary to esophageal varices or another complication of portal hypertension such as portal hypertensive gastropathy. This is the most concerning, life-threatening bleed that you will treat for, however you also consider the following diagnoses:

- Aorto-enteric fistula (if there is a history of abdominal aortic aneurysm or aortic graft)
- Angiodysplasia (in patients with renal disease, aortic stenosis – Heyde syndrome)
- Peptic ulcer disease related to H pylori infection or NSAID use
- Malignancy
- Marginal ulcers (in a patient with a history of gastric bypass)

1. Blood transfusion. The landmark TRICC^2^ trial looked at restrictive (Hb >7) versus liberal (Hb>10) transfusion strategies in critically ill patients who were euvolemic after initial fluid resuscitation. The restrictive transfusion group had lower rates of in-hospital mortality compared with the liberal strategy. The major critique of this study was that actively bleeding patients were excluded. The subsequent Villaneuva trial^3^ specifically looked at 921 patients with acute upper GI bleeding randomized to restrictive (7 or greater) or liberal (9 or greater) transfusion strategies. The restrictive strategy had a 45% relative risk reduction in all-cause mortality at 45 days (5% versus 9%), shorter length of stay, and fewer adverse events.

2. Acid suppression. Begin with a bolus of 80mg of IV pantoprazole or omeprazole and continue at twice daily dosing of 40mg until the source of bleeding has been uncovered. Proton pump inhibitors (PPIs) neutralize gastric acid leading to coagulation and subsequent hemostasis. Previously, it was thought that an initial bolus followed by continuous infusion of a PPI was necessary for acute UGIB, however a meta-analysis in 2014^4^ demonstrated noninferiority for intermittent dosing, resulting in a change in the European guidelines.

3. Start a somatostatin analogue if there is concern for a variceal bleed. We use octreotide at all of our institutions (although terlipressin is used in Europe) and continue for 3-5 days after confirmation of the diagnosis. Although the effect on mortality is unclear, mechanistically it reduces splanchnic blood flow and to GI angiodysplasia.

4. Start antibiotic prophylaxis if there is concern for cirrhosis. There is mortality benefit to doing so in this patient group, most commonly ceftriaxone 1g daily (maximum 7 days).

5. Correct coagulopathy. There is actually no definitive evidence for this strategy, but often patients with acute upper GI bleed (especially those with cirrhosis, on anticoagulation) will have coagulopathy as evidenced by an elevated PTT or INR. Vitamin K, fresh frozen plasma, and prothrombin complex concentrate can be used to reverse coagulopathy and attempt to slow or stop bleeding. There are no guidelines on platelet transfusion in this clinical scenario.

**The GI fellow calls you before coming for the endoscopy and asks you to administer erythromycin now to the patient. What is the evidence for this?**

Prokinetic agents including erythromycin and metoclopramide can be used to improve gastric visualization at the time of endoscopy. It should be dosed somewhere between 30 and 90 minutes prior to the procedure. Erythromycin is a motilin receptor agonist, whereas metoclopramide enhances the response to acetylcholine in the GI tract therefore theoretically stimulating gastric emptying. One meta-analysis^5^ that reviewed 5 trials, 316 patients treated with erythromycin, metoclopramide, and placebo for this indication noted that prokinetic agents reduced the need for second-look endoscopy. However, they had no association with number of PRBCs transfused, hospital length of stay, nor need for surgery. Subgroup analysis gave erythromycin the edge over metoclopramide.

**Your critical care fellow suggests you add on thromboelastography (TEG) to your next set of labs so you can resuscitate the patient with better understanding of their coagulopathy. What does this testing entail and how does it provide more specific information about primary and secondary hemostasis?**

TEG provides a dynamic assessment of platelet integrity in whole blood. A cylindrical container holds a whole blood sample at 37 degrees Celsius and is oscillated over 10 seconds. Primary hemostasis occurs naturally over a small in immersed into the center of the cylinder. A transducer measures the properties of the fibrin-mesh clot formed via this pin. What is measured is as follows, with specific factors that can be used for repletion:

1. Clot initiation time (R time): how long it takes thrombin and fibrin to generation
   1. Prolonged with: clotting factor deficiency, hypofibroginemia
   2. Reverse with: FFP, protamine
2. Clot firmness (K) + Kinetics of clot initiation (alpha angle)
   1. Prolonged with: clotting factor deficiency, platelet dysfunction, thrombocytopenia
   2. Reverse with: cryoprecipitate
3. Clot strength (MA, maximum amplitude): GPIIb/IIIa platelet-fibrin interaction
   1. Lower with: platelet dysfunction // Higher with: hypercoagulable state
   2. Reverse with: platelet transfusion // antiplatelet agent
4. Clot lysis over time (Ly30, percent clot lysis 30 minutes after the MA is reached)
   1. Prolonged with: enzyme mediated or mechanical fibrinolysis
   2. Reverse with: tranexamic acid

TEG is a rapid test, used widely in anesthesiology and surgery departments. It’s utility in general practice/critical care is more uncertain.

**What are the contents of the different products you will consider transfusing and what complications of transfusion should you be aware of?**

Different products contain various components of primary and secondary hemostasis. A brief overview of physiology:

1. Primary Hemostasis: The Platelet Plug

- Intimal injury of endothelium results in exposure of collagen
- Thrombin production stimulated, causing platelet aggregation
- Glycoprotein Ib on the platelet wall links to von Willebrand factor (vWF), allowing the platelet adhesion process
- Fibrinogen links platelets to one another eventually forming the platelet thrombus

2. Secondary Hemostasis: The Clotting Cascade

***Intrinsic Pathway Extrinsic Pathway***

XI XIa VII TF (vascular injury)

IX IXa TF – VIIa

VIII VIIIa

X Xa X

V Va

Prothrombin Thrombin

Fibrinogen XIIIa XIII

Soluble fibrin Crosslinked fibrin

*Packed red blood cells (PRBCs)*: For active hemorrhage, hemoglobin <7mg/dL, or poor oxygen delivery.

*Platelets*: Indications include absolute platelet count <10K to prevent spontaneous hemorrhage, <50K for those undergoing invasive procedures, <100K for CNS injury/neurosurgery/intrathecal catheter placement.

*Plasma Products*:

1. Fresh frozen plasma (FFP): Separated from fresh drawn blood by removing the red blood cells, white blood cells, and platelets.

2. Cryoprecipitate: Made by thawing FFP and collecting the white precipitate, providing large quantities of vWF, factor VIII, factor XIII, and fibrinogen. The volume is significantly smaller than FFP.

3. Prothrombin complex concentrate (PCC): Vitamin-K dependent factors (II, VII, IX, and X).

References

1. Laine L. Upper gastrointestinal bleeding due to a peptic ulcer. N Engl J Med 2016; 374:2367-2376.

2. Hebert PC, et al. A multicenter, randomized, controlled clinical trial of transfusion requirements in critical care. The New England Journal of Medicine. 1999. 340(6):409-417.

3. Villanueva C et al. Transfusion strategies for acute upper gastrointestinal bleeding. The New England Journal of Medicine. 2013. 368(1):11-21.

4. Sachar H, Vaidya K, Laine L. Intermittent vs continuous proton pump inhibitor therapy for high-risk bleeding ulcers: a systematic review and meta-analysis. JAMA Intern Med 2014;174:1755-1762.

5. Barkun AN, Bardou M, Martel M, et al. Prokinetics in acute upper GI bleeding: a meta-analysis. Gastrointest Endosc 2010; 72:1138.

**12. Toxidromes**

Learning Objectives

1. List common poisoning syndromes (toxidromes) and associated clinical signs and symptoms.
2. Describe the initial management strategies for the critically ill patient with presumed overdose.
3. Discuss treatment strategies for calcium channel blocker (CCB) overdose.
4. Identify unique treatment strategies for various ingestions.

Case

It’s a busy night in the medical ICU. The emergency department calls you with two admissions. You go down to see them and discuss each with the resident in the ED, after which you agree to admit both. Your buddy resident is also managing a decompensating patient in the medical ICU and you both work on the three cases together.

CASE 1:

A 36-year-old unresponsive female is brought to the emergency room by the police. They were called to her residence by her husband who found her at home after work with empty pill bottles at her side. He had last spoken to her about four hours prior, at which time she had sounded her normal self. He reports she had filled the prescriptions the day prior, so you assume she has ingested full quantities of each medication. He knows she was being treated for depression and insomnia but is unsure of the names of medications and doses. He agrees to go home and bring in all of the pill bottles he found.

On physical exam:

Vital signs with T 103.3F, HR 124bpm, BP 164/99 mmHg, RR 25/min, SpO2 94% on ambient air. She does not arouse to verbal stimuli but does withdraw extremities to pain and is moving all extremities spontaneously in the hospital bed. Pupils are dilated and reactive to light, bowel sounds are hyperactive. Reflexes are normal in the upper extremities and increased in the lower extremities with 8 beats of clonus present bilaterally.

CASE 2:

A 24-year-old male is brought to the emergency room by ambulance. His mother is with him. He has a history of depression, which has recently been uncontrolled. He and his mother relay that he had an intentional suicide attempt by ingestion of 30 tablets of his mother’s diltiazem 240mg ER about one hour prior to presentation. He currently complains only of nausea and mild lightheadedness.

On physical exam:

Vital signs with T 98.2F, HR 62bpm, BP 95/60 mmHg, RR 18/min, SpO2 96% on ambient air. He was awake, alert, oriented and neurologic exam was non-focal. Pulses were equal bilaterally but 1+ with capillary refill <2 seconds. Lung fields were clear, abdomen was benign, and no peripheral edema was present.

CASE 3:

Your buddy resident was called about one of your patients when the nurse noted his urine output had dropped off over the preceding three hours and sudden increase in vasopressor requirements. The patient is a 31-year-old male with past medical history of who was initially admitted for status epilepticus after loss of access to insurance and running out of his medications. He was started on burst suppression with propofol and attempts to wean him had been unsuccessful, resulting in recurrent episodes of his status. Earlier today, he had been febrile to 39C and cultures drawn at that time were currently negative.

On physical exam:

Vital signs with T 99.8F, HR 140bpm, BP 80/40 mmHg on AC/CMV 400/12/8/50% with SpO2 85%. The patient was sedated with an unchanged exam compared to earlier that day. The ABG drawn by the nurse was 7.2/25/80/12.

Questions

**What are the typical physical exam findings for the most common toxicological overdose syndromes?**

Adapted from Chu et al, Update in Clinical Toxicology^1^:

| **Syndrome** | **Vitals** | **CNS Effects** | **Peripheral Effects** |
| --- | --- | --- | --- |
| Anti-cholinergic | Hyperthermia  Tachycardia | Hallucinations, confusion, sedation, seizures | Decreased GI motility  Dry mucosa/skin  Urinary retention  Mydriasis |
| Cholinergic | Bradycardia | Confusion, lethargy, seizures | Salivation  Lacrimation  Urinary retention  Diarrhea/emesis/GI motility  Bronchorrhea/bronchospasm |
| Opioid | Bradycardia  Hypotension | Overall depression | Decreased GI motility  Miosis  Respiratory depression |
| Sedative-hypnotic | Bradycardia  Hypotension | Overall depression | Respiratory depression |
| Benzodiazepine | Normal | Overall depression | Respiratory depression (if concomitant CNS depressants) |
| Sympathomimetic | Hyperthermia  Tachycardia  (dysrhythmia)  Hypertension | Agitation, hallucinations, seizures | Diaphoresis |

**What toxicologic syndromes are on your differential diagnosis for the patients in Cases 1 and 3?**

Case 1: serotonin syndrome, anticholinergic toxidrome, neuroleptic malignant syndrome, malignant hyperthermia

Case 3: sepsis (sinusitis, ventilator-associated pneumonia, catheter-associated urinary tract infection), drug-induced liver injury, propofol infusion syndrome

**What labs should you consider in all of these patients besides a BMP, LFTs, CBC, and urine toxicology screen?**

- Evaluate for additional ingestions with serum ethanol, salicylates, and acetaminophen levels.
- Consider evaluation for toxic alcohols if there is a high anion-gap metabolic acidosis.
- Evaluate for rhabdomyolysis with a urinalysis and CK, especially if there is evidence of renal insufficiency.
- Obtain a lactate if there is any concern for poor peripheral perfusion or there is an anion gap metabolic acidosis present.
- Consider DIC labs if there is evidence of anemia or thrombocytopenia and then obtain a peripheral blood smear (looking for schistocytes), LDH, d-dimer, PTT/PT/INR, and fibrinogen. You can also consider a TEG if any of these values are severely deranged and there is need for product transfusions.
- Obtain an EKG to assess for any arrhythmia or interval prolongation that may suggest impending hemodynamic instability.

**What components of the history may be crucial to ask family members or loved ones?**

- History of psychiatric disease (namely depression, bipolar disorder, schizoaffective disorder) and any history of suicide attempts
- Family history of psychiatric disease
- Medications that the patient may have had access to (old medications, family members’ medications)
- Last known normal
- Hobbies or daily work with any implicated substances

**Based on your history and physical exam, what is your diagnosis for the patient in the first case and what treatment will you initiate?**

Serotonin syndrome. Treatment is typically supportive but can require invasive measures in severe cases:

- Hold all home medications/serotonergic agents.
- Supplemental oxygenation and intravenous crystalloid fluid replacement.
- Sedation with benzodiazepines (avoid restraints as they will lead to worsening lactic acidosis and hyperthermia).
- Severely hyperthermic patients (>41C) may require neuromuscular blockade and intubation.
- If there is evidence of autonomic instability, consider treatment with a serotonin antagonist such as cyproheptadine and vasopressors (phenylephrine, epinephrine, and norepinephrine preferred over dopamine given its metabolism to epinephrine and norepinephrine at the cellular level, which can potentiate the hemodynamic response.

**What is the typical course for serotonin syndrome?**

Symptoms usually have a rapid onset (within 24 hours) and offset after serotonergic agents are held (24 hours). This is contrasted with NMS, which may develop progressively over the course of weeks. Prognosis is favorable in serotonin syndrome.

**What additional clinical and serologic evaluation can be helpful in Case 2?**

- Evidence of volume overload may be seen in patients developing heart failure from calcium channel blocker overdose.
- Serum glucose may be elevated related to the inhibition of calcium-mediated insulin release.

**Would you consider gastric lavage for the patient in Case 2?**

This may be necessary in patients who present within 1-2 hours of a potentially dangerous ingestion (5-10 times the standard dose). However, the vagal stimulation from lavage may further precipitate bradycardia and hypotension. Consult with toxicology before moving forward with this.

**What are the manifestations of CCB overdose and the concurrent steps of management?**

The main hemodynamic considerations in the treatment of CCB overdose are rectifying refractory bradycardia and hypotension. Patients clinically may appear well and symptomatic in the early stages after ingestion despite these parameters being abnormal.

1. Hypotension 🡪 Intravenous fluids
2. Bradycardia 🡪 Atropine
3. IV calcium salts (overcome cardiovascular effects of CCBs; often ineffective)
4. IV glucagon (increases intracellular cAMP and increases heart rate in CCB toxicity but does not affect MAP)
5. IV high-dose insulin and glucose (high dose insulin has positive inotropic effects in patients with CCB toxicity)
6. Vasopressors (norepinephrine preferentially, with subsequent addition of epinephrine)
7. Lipid emulsion therapy (binding of the lipophilic drug and therefore decreased efficacy)
8. Transvenous pacing (if refractory bradycardia and shock)
9. Intra-aortic balloon pump
10. Extra-corporeal membrane oxygenation (last resort, case-by-case basis)

**What other labs will you ask for to confirm your suspicion for propofol infusion syndrome (PRIS) in Case 3?**

Cardiovascular:

- Widening QRS complex
- Brugada syndrome-like patterns
- Ventricular tachyarrhythmias
- Cardiogenic shock
- Bradyarrhythmias and asystole

Skeletal muscle:

- Myopathy
- Rhabdomyolysis
  - Hyperkalemia
  - AKI

Metabolic:

- Lactic acidosis 🡪 worsening hyperkalemia

**What are risk factors for PRIS?**

- Risk increases with length of infusion (more than 3 days) or higher rates on infusion, at least 5mg/kg/hour and doubles in patients receiving 6mg/kg/hour
- Shock, especially with the use of catecholamine vasopressors
- Use of glucocorticoids
- Carbohydrate depletion (liver disease, starvation, malnutrition)

**What is the treatment for PRIS?**

Treatment is supportive. There is no dedicated therapy nor antidote. Most patients who have developed PRIS have died, and all therapeutic options come from case reviews.

1. Immediately discontinue propofol.
2. Utilize RRT and bicarbonate for correction of metabolic acidosis.
3. Consider RRT also for hyperkalemia and AKI with rhabdomyolysis.
4. Bradyarrhythmias can be managed with transvenous pacing in select cases.
5. ECMO can be considered in refractory cases.

The best treatment is prevention by limiting the quantity and duration of propofol exposure in critically ill patients.

References

1. Chu J, Wang RY, and Hill NS. Update in clinical toxicology. Am J Respir Crit Care Med Vol 166. pp 9–15, 2002.

2. Mirrakhimov AE, Voore P, Halytskyy O, Khan M, Ali AM. Propofol Infusion Syndrome in Adults: A Clinical Update. Crit Care Res Pract. 2015; 2015: 260385.

**13. Cardiac Arrest**

Learning Objectives

1. Discuss an approach as the team leader/physician leader with regards to logistics of a code event.
2. Outline the algorithmic approach to cardiac arrest.
3. List the most common causes of pulseless electrical activity.
4. Compare and contrast post-ROSC strategies of out-of-hospital and in-hospital cardiac arrest.
5. Describe the assessment for neurologic recovery after cardiac arrest.

Case

You are called to a code on the surgical floor. The patient is a 56-year-old previously healthy male who recently underwent a left total knee replacement. The surgery was uncomplicated, however he had chest pain post-operatively when he arrived to the floor. When the EKG tech came to the room to perform a 12-lead, he found the patient unresponsive and called a code. You arrive and chest compressions are being performed by a nurse on the floor. The patient’s nurse and surgical intern arrive shortly after you and give you a brief history. They report all of the patient’s labs were normal this morning pre-operatively.

Questions

**Where do you position yourself in the room and how do you address the logistics of the room and personnel present?**

The leader of the code (you!) should remain at the head of the bed. It is important to establish your role from the moment you walk into the room if there is not already a code leader present. Additional individuals to identify include the patient’s nurse, who is documenting the events of the code, who is checking for a pulse, who is doing compressions (and who is lined up for relief), who will be managing the airway with bag-mask ventilation, and if a pharmacist is present. Quickly scanning the room and identifying individuals in each of these roles early on will be crucial to organization moving forward. You can then ask the recorder what medications have been administered and how long has passed since the code began to determine additional management. If the cardiac rhythm at the time of the code is known, this will also be important to move along the pathway.

**The provider whose hand is on the patient’s pulse reports a backboard was placed under the patient already. You identify everyone in the room, clarify their roles quickly, and ask what the cardiac rhythm at the time of arrest was. The recorder reports they did not get a good look at the rhythm. How do you proceed?**

The rhythm will determine the pathway you choose to manage this patient. Cardiac arrest is managed with basic life support (BLS) or advanced cardiovascular life support (ACLS) for in-hospital and out-of-hospital events depending on the availability and training level of the providers. Most commonly in the hospital setting, we run ACLS algorithms given our training and the personnel available for aggressive work-up and treatment. The basic algorithm is outlined below:

1. Check for signs of breathing and measure carotid or femoral pulse for 10 seconds or less.

2. If there is no pulse and no chest rise/breathing appreciated, activate a Code and begin resuscitation with chest compressions.

3. Begin bag-valve ventilation and attach a cardiac monitor to check the rhythm.

Shockable rhythm (VT, VF) Non-shockable rhythm (PEA, asystole)

Deliver shock^#^ Give epinephrine (1mg q3-5 minutes)

Rhythm check: still VT/VF* CPR x2 minutes

Give epinephrine Consider reversible causes:

(1mg q3-5 minutes) *Hypovolemia 🡪 1-2 L of NS or LR

*Hypoxia 🡪 Intubation (PETCO2 35-40mmHg)

Consider amiodarone *Hydrogen ion (acidosis) 🡪 50mEq bicarb

(300mg once) *Hypo/hyperkalemia 🡪 bicarb + calcium; HD

*Hypothermia 🡪 warming

*If the rhythm changes to a *Tension pneumothorax 🡪 needle decompression

non-shockable rhythm at any point, *Tamponade (cardiac) 🡪 pericardiocentesis

switch to the non-shockable pathway. *Toxins

^#^Shocks should be 120-200 joules (biphasic defibrillator) *Thrombosis (coronary, pulmonary) 🡪 tPA

or 360 joules (monophasic defibrillator).

4. Once ROSC is achieved, move to post-cardiac arrest care.

**You ask the team to stop for a pulse check when they are able and during this time, you assess the cardiac rhythm. You see electrical activity, but the report from the provider holding the femoral pulse is that the patient remains pulseless. You do not see any evidence of chest rise or spontaneous breathing. You tell the team to resume compressions. What other management steps should you take?**

You have identified pulseless electrical activity with associated cardiac arrest. This is a non-shockable rhythm. You ask the pharmacist and one of the nurses delivering medications to prepare and give 1mg epinephrine. As the code continues, you discuss the possibilities for reversible causes aloud and consider giving bicarbonate and calcium. You ask your timer to let you know when another 2 minutes have expired to repeat a pulse check.

**At the next pulse check, the provider reports she feels a faint femoral pulse. A blood pressure is obtained manually at 60/30. What are your next steps?**

You ask the team to deliver additional epinephrine and begin a bolus of whatever fluids are available (usually normal saline or Lactated Ringer’s on the code cart). You also ask them to prepare whatever pressor is available. Dopamine is typically stocked on code carts because it has the longest shelf life. You can begin this while you await transport to the ICU or if the patient maintains a stable blood pressure, have the pharmacy prepare your pressor of choice. This may also be an appropriate time to secure an airway with an endotracheal tube if this has not been done already.

**You travel with transport to the ICU and the patient is successfully moved over to the ICU bed. Now what?**

If it hasn’t already been done, you ask for a full set of labs (BMP, CBC, LFTs, INR, troponin, lactate), 12-lead EKG, and chest x-ray. You then perform a thorough exam. You note that the patient does not have any evidence of response to verbal nor noxious stimuli. You determine it is appropriate to move forward with therapeutic hypothermia.

Targeted temperature management (TTM) or therapeutic hypothermia is utilized to provide neuroprotection after cardiac arrest, minimizing anoxic brain injury and resultant reperfusion injury, often associated with cerebral edema. Original guidelines suggested 32-34 degrees Celsius as the goal, however there is evidence that 36 degrees Celsius results in the same mortality and neurologic events with less adverse effects of a lower core body temperature (coagulopathy, shivering, etc)^1^. Evidence in patients with shockable rhythms (VF, VT) suggests improved survival and neurologic outcomes, whereas evidence in patients with non-shockable rhythms (PEA) is conflicting.

TTM is typically used in out-of-hospital cardiac arrest patients. There is significant debate over the use of TTM for in-hospital arrest. There is evidence that a labile core temperature is associated with increased mortality for in-hospital arrest, but without any significant benefit in recovery of neurologic function. Regardless, it is an AHA Class IIb indication to perform TTM for in-hospital cardiac arrest of any rhythm.

During TTM, take the following into consideration:

- Therapeutic hypothermia should be initiated within 8-12 hours after ROSC.
- Initial brain imaging with CT should be performed.
- Patient is intubated and mechanically ventilated.
  - The goal PaCO2 is typically 40 mmHg or higher to avoid hypocapnia-induced cerebral vasoconstriction.
  - PaO2 >300 or hyperoxia is associated with worse outcomes. SpO2 should be maintained at 94% or greater.
- A core temperature probe must be placed (usually an endovascular catheter, similar to a central line).
- Sedatives of choice include Propofol and fentanyl (also minimize shivering).
- Neuromuscular blockade can be utilized (but may mask seizure activity).
- Mitigate shivering (blankets, sedatives including benzodiazepines).
- Maintain core temperature at target for at least 48 hours and subsequently rewarm patient over 12-24 hours.

Absolute contraindications for therapeutic hypothermia include a responsive patient with rapid and complete neurologic recovery or any desire for limited resuscitative efforts.

**While your ICU team sets up the devices needed for TTM, you place the endovascular cooling probe with ultrasound-guided femoral access. You take a minute to sit down and think about your next steps in evaluation of the etiology for the patient’s cardiac arrest. How will you evaluate each of your considerations?**

His chest pain preceding the event and age put him at risk for an acute coronary syndrome as the etiology for his arrest. We classically think of VT and VF arrest in patients with ACS, but PEA also occurs. Your 12-lead EKG does not demonstrate any deep Q waves nor ST elevations to suggest infarct or injury. His initial troponin in 0.5.

His recent surgery puts him at risk for pulmonary embolus, which can cause PEA arrest as well. His creatinine post-code is 2.0 from a normal baseline, so you cannot immediately evaluate this with a CTA chest, but you do consider anticoagulating him empirically with a heparin drip.

His labs reveal normokalemia, anion-gap metabolic acidosis with a lactate of 9, and transaminitis with AST and ALT in the 200s. His chest x-ray does not reveal cardiomegaly nor pneumothorax, and his ETT is in place 2cm above the carina.

**You decide to call the fellow to assist you with management and get the bedside echo ready to perform an assessment of his cardiac function grossly. What will you look for?**

- A large pericardial effusion
- Obvious LV or RV dysfunction
- RV pressure/volume overload or septal bowing
- Obvious mitral or tricuspid valve regurgitation

**You have supported the patient through 48 hours of TTM. Labs have remained stable to improved and you have diagnosed the etiology of their PEA arrest. It is time to rewarm the patient, and you do so uneventfully over the course of 12-24 hours as advised. The patient’s family asks you if they can speak to their loved one and if they are awake yet. How will you manage this patient, assess for irreversible anoxic neurologic injury, and how would you counsel this family?**

Definitions to consider:

- Coma: patients are unaware of their environment and unarousable, caused by dysfunction to the reticular activating system above the level of the mid-pons or dysfunction of both hemispheres.
  - May progress to recovery, persistent vegetative state or death within two weeks.
- Persistent vegetative state: patients are unaware, similar to coma, but have normal sleep-wake cycles and are arousable.
  - Deemed to be permanent after three months if induced non-traumatically.
- Brain death: irreversible cessation of cerebral and brainstem function with no respiratory drive, no spontaneous breathing, no cranial nerve reflexes, however spinal reflexes may be intact.

Treatment of patients with anoxic brain injury is mostly supportive and preventive. The following measures are important practices to consider in the post-arrest time period:

- Raising the head of the bed.
- Stress ulcer and DVT prophylaxis.
- Early occupational and physical therapy.
- Resume enteral feeds after therapeutic hypothermia is complete.
- Antibiotic prophylaxis has been used for pneumonia prevention, however without survival nor functional benefit.

Prognostic considerations:

- Duration of CPR correlates significantly with outcomes. In one study, no patient who required more than 15 minutes of CPR survived more than 6 weeks^2^.
- Age >70, history of CVA, ESRD, or recent CHF, and fever within the first 48 hours are all negative prognostic factors.
- Ventricular fibrillation or ventricular tachycardia have better outcomes than PEA arrest.
- Outcomes for out-of-hospital cardiac arrests have about 10.8% survival to discharge in those who receive resuscitation from EMS and about 22.3-25.5% among patients who received resuscitative efforts after in-hospital cardiac arrest in the US^3^.

Definition of Brain Death:

- Clinical or neuroimaging evidence consistent with a clinical diagnosis of brain death.
- Exclusion of other major medical conditions, intoxication nor poisoning.
- Core temperature >36 degrees C.
- Systolic blood pressure >100mmHg (which may include the use of vasopressors).
- Neurologic exam with absent cerebral or brainstem function (pain responses, pupillary light reflex, corneal reflex, oculovestibular reflex, jaw jerk, gag, cough, sucking/rooting reflex).

Testing involved:

- Apnea test: disconnecting the ventilator after preoxygenation (FiO2 1.0 for 10 minutes up to PaO2 200mmHg or greater or PaCO2 greater than 40 mmHg), positive if no respiratory response to PaCO2 >60mmHg or 20mmHg greater than baseline values and final arterial pH of <7.28.
- Criteria for the examiners varies by state. In Colorado, one physician needs to perform the exam, document the etiology and irreversibility of the condition, clinical criteria met, and date/time of death (when Apnea test was done).

If traditional testing (above) is unable to be performed, consider:

- EEG
- CT angiography
- Radionuclide scanning
- MRA brain with absent blood flow
- Transcranial dopplers

It can take a significant amount of time to begin to see neurologic recovery after cardiac arrest.. Families should be counseled that there is no way to reliably predict how much neurologic recovery an individual patient will have, usually time will tell. A formal brain death test can be performed if there is no evidence of neurologic recovery 48 hours following rewarming.

References

1. Nielsen N, Wetterslev J, Cronberg T, et al. Targeted temperature management at 33°C versus 36°C after cardiac arrest. N Engl J Med 2013; 369:2197.

2. Berek K, Jeschow M, Aichner F. The prognostication of cerebral hypoxia after out-of-hospital cardiac arrest in adults. Eur Neurol 1997; 37:135.

3. Kleinman ME, et al. Part 5: Adult Basic Life Support and Cardiopulmonary Resuscitation Quality: 2015 American Heart Association Guidelines Update for Cardiopulmonary Resuscitation and Emergency Cardiovascular Care. Circulation. 2015 Nov 3;132(18 Suppl 2):S414-35.

**14. Alcohol Withdrawal and Complications**

Learning Objectives

1. Describe the clinical and serologic assessment of the patient with alcohol withdrawal and its complications.
2. Compare and contrast strategies for monitoring alcohol withdrawal in the hospital (including CIWA, SEWS, and MINDS protocols).
3. Discuss the evidence for symptom-triggered versus scheduled medications for alcohol withdrawal.
4. List treatment options for alcohol withdrawal and its complications.

Case

The emergency department calls you to admit a 43-year-old woman who presents with severe alcohol withdrawal. She has a history of alcohol withdrawal seizures, last admitted for this one month ago, but has never required intubation during withdrawal episodes. She is well-known to the staff and typically drinks about a pint of vodka or whiskey daily. She suffers from anxiety and depression and has been unable to find a consistent primary care provider, psychologist, nor psychiatrist to manage her symptoms. Her husband brings her in as he found she had stolen a few bottles of whiskey from a birthday party they went to the previous night. Her last drink was about four hours prior to presentation.

Vitals: T 99F, BP 155/95 mmHg, HR 110 bpm, RR 22/min, SpO2 94% on ambient air

Physical exam is notable for disorientation (to place and situation) with noted visual hallucinations, tremulousness, tongue fasciculations, mild epigastric tenderness, and ataxic gait.

Labs reveal WBC 8, Hb 14, platelets 180, Na 139, Cr 1.1, AST 112, ALT 60, Tbili 2, lipase 200. Her blood alcohol level is 320.

An EKG is notable for sinus tachycardia and a CXR is unremarkable.

Questions

**Which stage of alcohol withdrawal would you classify this patient within? Why is important to admit this patient to the intensive care unit?**

There are four clinical stages of alcohol withdrawal:

| **Stage** | **Name** | **Clinical Features** | **Onset After Cessation** |
| --- | --- | --- | --- |
| 1 | Minor withdrawal | Tremors, insomnia, irritability, agitation, nausea, vomiting, anxiety, sweating, restlessness, anorexia | 6-12 hours |
| 2 | Alcoholic hallucinosis | Auditory, visual, or tactile hallucinations | 12-24 hours |
| 3 | Withdrawal seizures | Typically tonic-clonic seizures | 12-48 hours |
| 4 | Withdrawal delirium (delirium tremens) | Typically visual hallucinations, disorientation, tachycardia, hypertension, agitation, diaphoresis, low-grade fever | Usually 3-7 days, up to 14 |

This patient appears to be in the fourth stage of withdrawal based on their vital signs and clinical symptoms. ICU admission should be considered in all patients with severe alcohol withdrawal (CIWA 20 or greater), hallucinations, need for sedation and monitoring, respiratory failure, hemodynamic instability, and comorbidities including gastrointestinal bleeding, ingestion/intoxication or other substances, sepsis, significant fluid or electrolyte derangements, or organ failure. Each hospital has a different comfort level for admitting patients with alcohol withdrawal to various levels of care. It is best to make a decision based off of clinical assessment, patient history, and ancillary staff impression/assistance.

**What complications of alcohol ingestion should be considered in this patient and/or are present based on evaluation thus far?**

Testing in patients with alcohol withdrawal includes basic chemistry (including magnesium and phosphorus to assess for electrolyte derangements), glucose (to assess for hypoglycemia), complete blood count (to assess thrombocytopenia, which may discern additional risk for severe withdrawal), alcohol and toxicology screening, liver function tests (including INR), lipase (to assess for concurrent pancreatitis if nausea and vomiting are present) and if the patient exhibits seizure-like activity or delirium additional imaging (CT of the head) and infectious work-up may also be considered (including consideration of a lumbar puncture). An ECG may be helpful to track the QTc in patients who may require anti-emetics.

**What would be your treatment strategy for this patient?**

Benzodiazepines are the first-line treatment in patients with alcohol withdrawal as they reduce the symptoms of withdrawal and seizures.

Regimens include:

- Symptom-triggered
- Fixed-dosing
- Loading dose protocols

Symptom-triggered is preferred in most institutions where patients can be closely monitored. If symptoms are not easily assessed, fixed-tapering may be useful. These strategies are equally effective for improving withdrawal symptoms, however symptom-triggered dosing can be associated with greater improvement in symptoms at 48 hours, shorter hospitalization, and less use of benzodiazepines overall^1,2^. In the elderly and those with severe liver disease, regimens with lorazepam and oxazepam are preferred given more predictable elimination, volume of distribution, and less accumulation of metabolites (other agents utilize P450 system, which can be impaired with advanced age and cirrhosis).

The CIWA-Ar (Clinical Institute Withdrawal Assessment – Alcohol, revised)^3^ is the most commonly recognized and utilized assessment scale for establishing baseline severity, monitoring symptoms, and treating subsequently.

At Denver Health we utilize the Severity of Ethanol Withdrawal Scale (SEWS) protocol on the inpatient floor units and MINDS (Minnesota Detoxification Scale) protocol in the ICU. As compared to the CIWA-Ar scoring, the SEWS scoring system decreases the length of treatment for alcohol withdrawal, with better early assessment and improved medication administration early in the course of the syndrome^4^.

The MINDS protocol has been validated in the ICU, whereas the CIWA-Ar has not. Littlefield and colleagues^5^ compared the CIWA-Ar and MINDS (modified) and found there was a strong correlation between the two in patiets in the ICU and step-down units. There was also a decrease in correlation between the two as alcohol withdrawal worsened.

In the ED, phenobarbital loading is often performed based on initial withdrawal assessment scores, given evidence in the emergency medicine literature for this treatment strategy to prevent severe withdrawal requiring ICU admission^6^.

Summary of Alcohol Withdrawal Treatment Protocols:

| **Protocol** | **Components** | **Medications Utilized** | **Specific Considerations** |
| --- | --- | --- | --- |
| CIWA-Ar | Nausea/Vomiting  Tremor  Paroxysmal Sweats  Anxiety  Agitation  Tactile Disturbances  Auditory Disturbances  Headache/Fullness in Head  Orientation/Clouding of Sensorium | Chlordiazepoxide 50-100mg PO  Diazepam 10-20mg PO  Lorazepam 2-4mg PO | *Scoring 0-67 pts  <8 absent  9-14 Mild  15-20 Moderate  >20 Severe  *Mostly mild to moderate withdrawal  *Scores 8 or greater necessitate treatment |
| SEWS | Anxiety  Nausea/Vomiting  Sweating  Tremor  Agitation  Orientation  Hallucinations  Vitals (HR, DBP, Temp) | Phenobarbital 65, 130, or 260mg IV  Lorazepam 2-4mg IV | *Scoring  High risk 6 or more  Low risk <6  *Includes VS (vs. CIWA)  *Does not include headache (vs. CIWA) |
| MINDS | Pulse  DBP  Tremor  Sweat  Hallucinations  Agitation  Orientation  Delusions  Seizures | Mild: q2h scoring  Lorazepam 1mg q1h PRN  Mod: q1h scoring  Lorazepam 2mg q1h PRN  Severe: q20 min  Midazolam infusion @ 5mg/hr, titrate q1h PRN  OR  Lorazepam 4mg q20min PRN | *Scoring 0-46 pts  Mild <15  Mod 15-19  Severe >19  *Specifically studied in the ICU setting |

You also order the patient high-dose thiamine given this is a recommendation in acute alcohol withdrawal, folic acid, and a multivitamin. You plan to follow electrolytes closely.

**You are called to the bedside as the patient is becoming more agitated despite the use of benzodiazepines per the MINDS protocol. What adjunctive therapies can you consider and what side effects should you be aware of?**

These medications should not be used alone to treat withdrawal, are less well studied and may mask the hemodynamic instability of withdrawal that precedes seizures.

- Dexmedetomidine (alpha-2 agonist)
  - Benefits: no respiratory suppression, short half-life (onset less than 5 minutes, peak effect in 15 minutes)
  - Adverse Effects: bradycardia, hypotension, dry mouth, nausea
- Antipsychotic agents: quetiapine, olanzapine (dopaminergic)
  - Benefits: treatment of concomitant thought disorder
  - Adverse Effects: prolonged QTc, extrapyramidal symptoms, dystonia, lower the seizure threshold

If the patient is intubated or refractory to high doses of benzodiazepines (in which case intubation should be a consideration), think about:

- Propofol (GABA agonist)
- Phenobarbital (barbiturate)
  - One RCT^3^ of 198 patients presenting to the ED studied single dose of IV phenobarbital and symptom-guided lorazepam versus placebo with symptom-guided lorazepam resulted in decreased ICU admission (8% versus 52%) without increased adverse outcomes.
  - Studies in patients with refractory DTs also demonstrate a role for phenobarbital (dosed 130 to 260 mg IV, repeated every 15 to 20 minutes, until symptoms are controlled).
- Midazolam drip (GABA agonist) [avoid lorazepam given its longer half-life and trend towards prolonged intubation and oversedation; also can cause increased delirium (drug toxicity), increased LOS, and increased cost]

References

1. Saitz R, Mayo-Smith MF, Roberts MS, et al. Individualized treatment for alcohol withdrawal. A randomized double-blind controlled trial. JAMA 1994; 272:519.

2. Daeppen JB, Gache P, Landry U, et al. Symptom-triggered vs fixed-schedule doses of benzodiazepine for alcohol withdrawal: a randomized treatment trial. Arch Intern Med 2002; 162:1117.

3. Sullivan J. et al. Assessment of alcohol withdrawal: the revised clinical institute withdrawal clinical institute withdrawal assessment for alcohol scale. British Journal of Addiction. 1989;84:1353-1357.

4. Beresford T, et al. The Severity of Ethanol Withdrawal Scale in Scale-Driven Alcohol Withdrawal Treatment: A Quality Assurance Study, Alcoholism Treatment Quarterly, 2017, 35:3, 232-242.

5. Littlefield AJ, et al. Correlation Between mMINDS and CIWA-Ar Scoring Tools in Patients With Alcohol Withdrawal Syndrome. Am J Crit Care July 2018 27:280-286.

6. Rosenson J, Clements C, Simon B, et al. Phenobarbital for acute alcohol withdrawal: a prospective randomized double-blind placebo-controlled study. J Emerg Med 2013; 44:592.

**15. Hyponatremia**

Learning Objectives

1. Outline the diagnostic approach to hyponatremia and possible causes.
2. Describe the management of hyponatremia as related to severity.
3. Differentiate the mechanism of hyponatremia in patients with cirrhosis, heart failure, and renal disease.
4. Discuss the complications of severe hyponatremia and overcorrection.

Case

A 60-year-old female is brought to the emergency department by her husband after he noticed her having seizure-like activity at home. She was seemingly at her baseline the morning prior to presentation then became acutely confused about 2-3 hours before presenting. At 3pm in the afternoon she was laying down and started shaking her arms and legs in rhythmic movements and subsequently vomited. Her husband noted she was incontinent of urine during this episode and profoundly confused, unable to participate in conversation afterwards.

On exam T 97.3F, HR 79 bpm, RR 13/min, BP 90/63 mmHg, Spo2 100% on room air

She was alert but not oriented to person, place, nor time, and not cooperating with questions but otherwise in no distress. Pupils were 4mm bilaterally and sluggishly reactive. No visible tongue lacerations were present. Cardiopulmonary and abdominal exams were unremarkable. She was moving all extremities spontaneously and had no clonus.

Labs included WBC 11.8, Hb 13.7, platelets 331

Troponin <0.02

Na 115, K 3.8, Cl 79, CO2 15, glucose 112, Cr 0.57, AST 38, ALT 20

Serum alcohol <10, APAP <2, salicylate <1.7

Urinalysis with trace ketones, no protein, no blood, no WBC nor RBC

Urine toxicology negative

Questions

**What is your initial approach to the patient for hyponatremia? How would you characterize this patient’s hyponatremia?**

Think about the following:

1. Duration: acute (less than 48 hours) or chronic (present for more than 48 hours or unknown)

- Acute: greater risk of immediate complications and greater need for aggressive therapy
- Chronic: higher risk of osmotic demyelination with aggressive correction

2. Severity: numeric classification or by symptoms

- Severe: Na <120 mEq/L OR seizures, obtundation, coma, cardiac arrest
- Moderate: Na 120-129 mEq/L OR headache, fatigue, lethargy, nausea, vomiting, gait disturbance, confusion, muscle cramps
- Mild: Na 130-134 mEq/L (symptoms similar to moderate)

This patient’s hyponatremia is severe given the presence of sodium < 120 mEq/L and seizure, obtundation at the time of presentation.

Goals of treating hyponatremia focus on the following:

- Preventing additional decline in the serum sodium while concurrently avoiding rapid overcorrection (patients at highest risk of this include those with hypokalemia, alcoholism, malnutrition, and liver disease)
- Prevent brain herniation due to cerebral edema
- Relieving symptoms related to hyponatremia

**What additional work-up would you obtain at this time?**

Many patients with hyponatremia have more than a single cause for the fall in plasma sodium. Hyponatremia is a disorder of water secretion, which is typically dependent on antidiuretic hormone (ADH) function. There are multiple ways to approach the patient with hyponatremia, with a few outlined below.

1. Focus the history and physical on fluid loss (vomiting, diarrhea, diuretics), if there is a predisposing condition (HIV, CNS disease, malignancy, heart failure, hepatic failure, plasma cell dyscrasia, pulmonary disease, psychiatric disease), and possible contributing medications (diuretics, SSRIs, antiepileptics) or drugs (MDMA), evidence of volume overload on exam, recent surgery, and signs of adrenal insufficiency.

2. Follow an algorithmic approach.

Utilize the history and physical exam to determine if hyperglycemia or a hyperosmolar state is at play.

Correct Na for hyperglycemia.

Assess for the presence of solutes or lipemia, hyperbilirubinemia, or plasma cell dyscrasia.

Is cirrhosis, decompensated heart failure, or renal disease playing a role?

Check urine Na and osmolarity to evaluate for sodium avidity of renal tubules (urine Na <20).

Assess for the presence of endocrine disease: thyroid disorders, glucocorticoid deficiency.

Based on the above, we could algorithmically work the patient up. Often times, baseline labs are obtained including serum glucose, creatinine, complete blood count, liver function tests, markers of cardiac disease (EKG, troponin, BNP), TSH, and AM cortisol to assess for specific disease states (bolded in blue).

3. Urine excretion of sodium is relatively independent of plasma sodium, and therefore can help distinguish between SIADH and hypovolemic hyponatremia^1^. Vasopressin (ADH) is usually present when the plasma sodium is above 135 mmol/L and levels increase linearly as the plasma sodium increases. If there is inadequate effective arterial blood volume, vasopressin is also secreted. ADH is “inappropriate” when there is no osmotic (sodium level related) nor hemodynamic (effective circulating blood volume) stimulus. When it is secreted, ADH binds the V2 receptor of the principal cells in the collecting duct of the renal tubule. Subsequently, the insertion of aquaporins in the luminal membrane allows for water to flow out (drawn this direction by the highly osmotic medulla of the kidney). At the upper limit of normal of sodium (145 mmol/L), ADH levels maximally concentrate the urine (1200 mosm/kg). If urine is dilute when plasma sodium is above 145 mmol/L, this indicates diabetes insipidus (central or nephrogenic). With SIADH, there is a high content of sodium in the urine despite hyponatremia because extra water retention causes increased extracellular fluid volume.

Other ineffective osmoles that can falsely elevate the serum sodium include urea and ethanol, and these should be taken into consideration if the algorithm takes you down the path of calculating serum osmolality.

Serum Osm = (2*[Na]) + (BUN / 2.8) + (glucose / 18) + (ethanol / 3.7)

**Your additional work-up is in process. What is your overall goal at this point?**

Increase the sodium by 4-6 mmol/L and absolutely no more than 8 mmol/L in the first 24 hours from the time of presentation. Rapid changes in the plasma sodium concentration can cause severe and permanent brain injury. Hyponatremia or profound hypotonicity can cause rupture of cell membranes, brain swelling, increased intracranial pressure, and eventual herniation. This progressive injury is called the osmotic demyelination syndrome and occurs in two phases:

1. Reduction in symptoms
2. Gradual onset of new neurologic findings including seizures, behavioral change, and movement disorders

Demyelination of the central pons causes the “locked in” phenomenon, which is typically what we associate as the most severe consequence of rapid overcorrection^1^.

*SCENARIO #1:*

**You receive labs back that the patient’s urine sodium is 8 mmol/L, urine creatinine is 20 mg/dL, and urine osm is 66 mmol/L. What additional labs would you like to assist with your diagnosis and how will you treat this patient?**

You choose to calculate the free water clearance. This is a surrogate for the amount of iso-osmotic urine produced. A value greater than zero implies dilute urine with solute-free water being excreted. Negative values imply the kidney is retaining or conserving solute-free water (likely due to ADH), which then results in concentrated urine. Calculating free water clearance in the urine:

ml/minute of urine produced – [ (urine osm / plasma osm) * ml/minute urine produced ]

Urine potassium was 5 and estimated free water clearance was 88%. You decide to manage the patient with hypertonic saline 3% at 15ml/hour for one hour. The literature supports 100ml boluses of 3% saline over an hour and rechecking the sodium (and repeating this up to three times in succession)^1^.

She underwent additional work-up for adrenal insufficiency, which was unremarkable. She did have a history of hypothyroidism and a TSH was found to be significantly elevated to 32 with a free T4 of 0.018, thought to be consistent with a diagnosis of myxedema coma. She overall was determined to have hypovolemic hyponatremia, with a component of severe hypothyroidism playing a role in her hyponatremia.

*SCENARIO #2:*

**You receive labs back that the patient’s urine sodium is 79 mmol/L and urine osm is 508 mmol/L. What additional labs would you like to assist with your diagnosis and how will you treat this patient?**

Treatment is similar to the first case. The patient should be treated with hypertonic saline given the severity of the hyponatremia and presenting symptom of seizure.

As part of the diagnostic work-up, consider why both the urine osmolality and urine sodium concentration is high. Thiazide diuretics may cause hyponatremia by prevention of sodium chloride reabsorption in the distal convoluted tubule. They can still concentrate the urine in the medulla and therefore can respond to ADH. Patients with thiazide-related hyponatremia can appear euvolemic and the metabolic abnormality usually occurs within 1-2 weeks of diuretic initiation.

Adrenal insufficiency can cause hyponatremia as the lack of cortisol does not feed back to appropriately inhibit ADH secretion. This increase in ADH release causes hyponatremia. Aldosterone deficiency in primary adrenal insufficiency also contributes to hyponatremia with significantly high urine sodium levels due to lack of reabsorption in the collecting duct.

Ecstasy (MDMA) can directly stimulate ADH release and increased thirst to prevent against hyperthermia. Additional complications of ingestion include hypertension, tachycardia, rhabdomyolysis and the serotonin syndrome. MDMA is readily screened for with urine toxicology.

The basic metabolic panel can assist with diagnosis. Hyperkalemia may occurs in primary adrenal insufficiency because of hypoaldosteronism. Hypoaldosteronism can also cause a Type IV renal tubular acidosis, resulting in a non-anion gap metabolic acidosis. Hypokalemia is seen with diuretic use, as well as alkalosis rather than acidosis.

Either of these could be the cause of the patient’s hyponatremia, and additional history from family members can give you clues towards the diagnosis.

**Why is it important to differentiate hyponatremia in the setting of chronic kidney disease, heart failure, and cirrhosis?**

They are pathophysiologically different entities from the other types of hyponatremia discussed above.

| *Disease State* | *Pathophysiology* |
| --- | --- |
| Heart failure | Decreased cardiac output and systemic blood pressure stimulates ADH, renin, and norepinephrine release. ADH enhances water reabsorption in collecting tubule. AT II and norepi limit distal water delivery/lower renal perfusion. Low cardiac output and high AT II stimulate thirst. |
| Cirrhosis | Systemic vasodilation lowers MAP and activates ADH, sympathetic nervous system, and RAAS. |
| Chronic kidney disease | Ability of the kidney to excrete free water is reduced when GFR declines < 15ml/min |

**What if you find that you have overcorrected a patient?**

If there are risk factors for osmotic demyelination or correction is 10-12 mmol/L or greater, desmopressin can be administered to preemptively halt additional rise in the serum sodium^2^. However, it should be cautioned that desmopressin does not have a clear dose-dependent effect on sodium and levels need to be monitored closely for excessive drops in serum sodium once again. Free water can also be given to match urinary output in order to maintain serum sodium at an appropriate level if the free water clearance is nearly iso-osmotic.

References

1. Sterns RH. Disorder of plasma sodium – causes, consequences, and correction. N Engl J Med 2015; 372:55-65.

2. Sood L, Sterns RH, Hix JK, Silver SM, Chen L. Hypertonic saline and desmopressin: a simple strategy for safe correction of severe hyponatremia. Am J Kidney Dis 2013;61:571-578.

**16. Management of the Patient with a Cerebrovascular Accident and Hypertensive Emergency**

Learning Objectives

1. Define criteria for the spectrum of hypertensive disorders.
2. Discuss the clinical complications seen with hypertensive crisis, work-up, and related treatment options.
3. Compare and contrast the approach to ischemic and hemorrhagic stroke in the setting of hypertensive crisis.

Case

A 57-year-old man is brought to the emergency department by his wife. She recalls hearing a thud at home about one hour prior and found her husband in their bedroom on the floor. He was awake but not responding appropriately and has not been able to move his left arm nor his left leg. She tells you he has a history of hypertension, atrial fibrillation and currently takes both aspirin and warfarin. He currently reports a headache.

Vitals: T98.2F, HR 88bpm, RR 28/min, BP 180/140mmHg , SpO2 96% on 4LPM nasal cannula

Exam reveals dense left-sided hemiplegia with left-sided facial droop, neglect and gaze palsy. Laboratory evaluation initially reveals:

WBC 8.6, Hb 17.2, platelets 225

Na 139, K 3.9, CO2 23, Cr 1.1, glucose 93, INR 1.5

A stroke alert is activated and both you and the neurology resident are called to the bedside in the emergency department. His NIH Stroke Scale is 20.

Questions

**How would you define this patient’s current clinical syndrome?**

Patients with significantly elevated blood pressure (systolic 180mmHg or greater and/or diastolic 120mmHg or greater) may be symptomatic or asymptomatic. When there is evidence of target organ damage and related symptoms, this is termed hypertensive emergency (also known as malignant hypertension). Hypertensive urgency is present when blood pressure is severely elevated without end-organ damage, also symptoms including headache, dyspnea, anxiety, epistaxis, or palpitations may be present.

Given likely end-organ damage in this patient with concern for ischemic or hemorrhagic stroke, we would define this as hypertensive emergency.

**What additional serologic and radiographic work-up would you perform in this patient and why?**

Work-up in hypertensive emergency is targeted towards identifying end-organ dysfunction:

- Serum creatinine to evaluate for renal dysfunction
- Complete blood count (and hemolysis labs if concerning) to evaluate for a microangiopathic anemia
- Cardiac biomarkers (troponin) if acute coronary syndrome is suspected
- BNP if heart failure is suspected
- Urinalysis to evaluate for microscopic hematuria, proteinuria, casts
- Electrocardiogram to assess for ischemia, infarction, or signs of left ventricular hypertrophy
- Chest radiography to assess for acute pulmonary edema
- Computed tomography or MRI of the brain to assess for acute infarct (hemorrhagic or ischemic)
- CT chest angiography or transesophageal echocardiography to assess for acute aortic dissection
- Urine or serum pregnancy test in females to assess for preeclampsia or eclampsia
- Urine toxicology if there is concern for ingestion of sympathomimetic agent

Consider secondary hypertension in patients who are < 30 years old, have resistant hypertension (on adequate doses of three or more medications, one of which is a diuretic), severe hypertension or hypertensive emergency presentation, or target-organ damage already present (left ventricular hypertrophy or retinopathy for example). In patients whom there is concern for a secondary cause of hypertension, consider work-up for:

- Thyroid dysfunction
  - TSH
- Obstructive sleep apnea
  - Polysomnography
- Hyperaldosteronism (hypokalemia, fatigue, constipation, muscle weakness)
  - Plasma aldosterone to renin ratio (>15 typically, most common cutoff >30)
- Renal artery stenosis (especially in young women or individuals who recently started an ACE inhibitor)
  - Renal ultrasound with dopplers
- Cushing disease (in patients with weight gain, striae, fatigue, truncal obesity, buffalo hump, psychological changes, polydipsia and polyuria)
  - Urinary free cortisol, dexamethasone suppression test, or late-night salivary cortisol level
- Pheochromocytoma (paroxysmal events of palpitations, hypertension, headache, flushing/perspiration, syncope, anxiety)
  - Blood preferred over urine for metanephrine and normetanephrine
- Coarctation of the aorta (arm-to-leg blood pressure measurements >20mmHg difference), delayed femoral pulse, murmur, headache, rib notching on CXR)
  - TTE including suprasternal notch view for arch visualization
- Primary hyperparathyroidism
- Congenital adrenal hyperplasia
- Mineralocorticoid excess syndrome

You obtain a portion of these labs in your patient. The troponin is negative, EKG with afib and no evidence of ischemia, CXR with bibasilar opacities possibly representing aspiration, urine toxicology and serum EtOH unremarkable, and urinalysis with 1+ blood, 0-1 RBC, no casts, no protein.

**You obtain a computed tomography scan of the head with angiography given the patient’s weakness on exam. It reveals acute hemorrhage in the right lentiform nucleus without vascular malformation and mild compression on the third ventricle with slight midline shift. What are your next management steps? How would this be different if there was an ischemic stroke or no evidence of an acute cerebral event?**

In most hypertensive emergencies, lowering the blood pressure too quickly or too much can can cause ischemic damage to vascular beds that have been habituated to a higher blood pressure because of autoregulation. In most cases, blood pressure should be reduced gradually by 10-20% in the first hour and further 5-15% over the next 23 hours. Most commonly this results in blood pressure <180/<120 in the first hour and subsequently <160/<110 in the next 23 hours. Systolic blood pressure or mean arterial pressure can be used in this calculation. Oral drugs can be used in hypertensive urgency (nicardipine, captopril, labetalol).

Exceptions to this rule include the following manifestations of hypertensive emergency^1^:

1. Acute ischemic stroke: Blood pressure should not be lowered unless greater than 180/110 mmHg in patients who are candidates for reperfusion (thrombolysis or thrombectomy).
2. Intracerebral hemorrhage: Risk for reducing cerebral perfusion versus reducing further bleeding are competing risks in blood pressure management. Typically, SBP should be lowered to 140 mmHg per guideline recommendations, with a range between 140-160, but not below this within the first hour, as this may increase the risk of adverse events.
3. Acute aortic dissection: Systolic blood pressure should be rapidly lowered to a target of 100-120mmHg and heart rate <60 bpm within 20 minutes to reduce shearing forces in the aorta and progression of the dissection.

For our patient, we should lower the blood pressure to between 140-160 systolic. The INTERACT2 trial^2^ randomly assigned 2800 patients with acute ICH and elevated blood pressure to intensive lowering (<140) within one hour or traditional management (<180). Intensive BP lowering was associated with improved measures of disability and adverse events were similar.

Multiple medications are approved for the treatment of hypertensive emergency and one should be initiated immediately as an IV infusion:

- Nicardipine (5-15 mg/hour) – can be used in pregnancy, caution in heart failure/ACS
- Clevidipine (1-21 mg/hour) – avoid in aortic stenosis, disordered lipids, or allergies to eggs or soy
- Nitroglycerin (5-100 mcg/minute) – additional benefit if ACS or pulmonary edema
- Nitroprusside (0.25-10 mcg/kg/minute) – avoid in patients with elevated ICP
- Fenoldopam (0.1-1.6 mcg/kg/minute) – avoid in glaucoma, sulfite sensitivity
- Labetalol (0.5-2 mg/minute) – avoid in asthma, COPD, heart failure, bradycardia, or AVB
- Esmolol (25-50 mcg/kg/minute) – avoid in heart failure, AVB
- Hydralazine (10-20mg IV boluses) – prolonged and unpredictable hypotensive effect
- Enalaprilat (1.25-5mg every 6 hours IV bolus) – slow and long duration so rarely use; avoid in LV failure, ACS, pregnancy, renal impairment
- Phentolamine (5-15mg bolus every 5-15 minutes) – for adrenergic crisis with pheochromocytoma or cocaine overdose

Additional Considerations:

- Consult neurosurgery in case of further clinical decompensation and need for surgical relief of increased cerebral edema.
- Consider risk versus benefit of reversing anticoagulation in the setting of intracerebral hemorrhage as well. FFP or PCC can be used for rapid INR reversal if supratherapeutic, DDAVP may be used to counteract aspirin.
- Consider giving intravenous fluids if the patient appears to be significantly volume depleted (pressure natriuresis and relative intravascular volume depletion) to avoid precipitous drops in blood pressure when anti-hypertensives are administered^3^.
- Patients who received tPA must be monitored in an ICU setting for 24 hours after receiving therapy for potential complications. Resumption of antiplatelet or anticoagulation is usually performed within 72 hours, but is up to the discretion of the medical team.

You start nicardipine (one of the two underlined preferred agents) with a goal systolic BP 140-160 mmHg and insert an arterial line for close blood pressure monitoring. You elect to hold off on additional fluid given concern for mild volume overload on exam.

**What additional stroke management and work-up should you consider for this patient?**

Physical therapy, occupational therapy, and formal speech and swallow evaluation must be pursued in any patient who suffers an ischemic or hemorrhagic stroke.

In embolic stroke, vascular assessment of the extracranial and intracranial arteries should be assessed if:

- Infarct is in the anterior circulation (carotids) 🡪 duplex US of neck and transcranial doppler of intracranial arteries
- Inafrct is in the posterior circulation (vertebrobasilar) 🡪 duplex US of origins of vertebral arteries +/-subclavian arteries

CTA or MRA of the head and neck may be sufficient.

For small vessel or lacunar stroke, patients should be assessed for underlying hypertension (BP assessment), dyslipidemia (lipid panel), diabetes mellitus (A1c), or polycythemia (CBC).

For ischemic stroke, all patients should have monitoring for the first 24 hours after stroke onset to look for occult atrial fibrillation. Ambulatory monitoring for subsequent 30 days should also be considered in cryptogenic ischemic stroke or TIA, with the CRYSTAL AF and EMBRACE trials^4,5^ noting higher rates of detection in monitored groups.

All patients with suspected embolic stroke should have an echocardiogram. Younger patients (<45 years old), those with a high pretest probability of negative TTE, those with atrial fibrillation and suspected left atrial appendage thrombus, those with a mechanical heart valve, or those with suspected aortic pathology should be considered for TEE rather than TTE up-front.

Hypercoagulable conditions should only be explored in patients with a personal or family history of systemic thromboses, those with no clear etiology for ischemic stroke or TIA despite cardiac and vascular imaging, or those with other systemic findings suggestive of lupus or the antiphospholipid antibody syndrome.

Atypical appearing hemorrhages may necessitate work-up for alternative, more rare etiologies including vasculitis, cerebral amyloid angiopathy, and vascular malformation or tumor.

References

1. Hemphill JC 3rd, Greenberg SM, Anderson CS, et al. Guidelines for the Management of Spontaneous Intracerebral Hemorrhage: A Guideline for Healthcare Professionals From the American Heart Association/American Stroke Association. Stroke 2015; 46:2032.

2. Anderson CS, Heeley E, Huang Y, et al. Rapid blood-pressure lowering in patients with acute intracerebral hemorrhage. N Engl J Med 2013; 368:2355.

3. Marik PE and Varon J. Hypertensive crises: Challenges and management. Chest 2007 Jun;131(6):1949

4. Sanna T, Diener HC, Passman RS, et al. Cryptogenic stroke and underlying atrial fibrillation. N Engl J Med 2014; 370:2478.

5. Gladstone DJ, Spring M, Dorian P, et al. Atrial fibrillation in patients with cryptogenic stroke. N Engl J Med 2014; 370:2467.
