# Supplementary material for: A Case-Based Critical Care Curriculum for Internal Medicine Residents Addressing Social Determinants of Health
Source: MedEdPORTAL. 2021 Mar 18;17:11128. doi: 10.15766/mep_2374-8265.11128 (PMC8015637; doi:10.15766/mep_2374-8265.11128)
Supplement: Supplementary file 1 — Needs Assessment.docxFacilitator Guide.docxSDOH Topics Guide.docxCritical Care Cases.docxMDR Checklist.docxPre- and Postcurriculum Surveys.docxCare Team Checklist.docxAttending Checklist.docx [file mep_2374-8265.11128-s001.zip › E. MDR Checklist.docx]

Trainees can use the following checklist during their multidisciplinary rounds (MDR) discussion of each patient.

- Provide a one-liner of the patient, focused history, and reason for intensive care unit (ICU) admission.
- Comment on any affirmative answers from your social determinants of health (SDH) screen:
  - Housing Instability
  - Interpersonal Safety
  - Transportation Needs
  - Food Insecurity
  - Utility Needs
- Acknowledge any known advanced care planning documentation the patient may have completed prior to or upon ICU admission.
- Identify the patient’s stated surrogate decision maker or proxy for the patient.
