# Supplementary material for: A Case-Based Critical Care Curriculum for Internal Medicine Residents Addressing Social Determinants of Health
Source: MedEdPORTAL. 2021 Mar 18;17:11128. doi: 10.15766/mep_2374-8265.11128 (PMC8015637; doi:10.15766/mep_2374-8265.11128)
Supplement: Supplementary file 1 — Needs Assessment.docxFacilitator Guide.docxSDOH Topics Guide.docxCritical Care Cases.docxMDR Checklist.docxPre- and Postcurriculum Surveys.docxCare Team Checklist.docxAttending Checklist.docx [file mep_2374-8265.11128-s001.zip › F. Pre- and Postcurriculum Surveys.docx]

**Critical Care and Health Disparities Curriculum**

Resident Pre-Rotation Survey

1. So we can compare pre- and post- survey answers, while maintaining your anonymity, please list the two digits for your birth month followed by your last two social. For example if you were born in January and your social ends in 56, enter 0156.

Knowledge:

2. I can identify and describe how social determinants of health affect quality of care for patients experiencing disparities in health care quality.

*Strongly Disagree*

*Disagree*

*Agree*

*Strongly Agree*

3. I can recognize more than one social domain that may contribute to a patient’s social risk.

*Strongly Disagree*

*Disagree*

*Agree*

*Strongly Agree*

4. I can name specific resources that may be valuable for patients who experience health disparities related to substance abuse.

*Strongly Disagree*

*Disagree*

*Agree*

*Strongly Agree*

5. I can name specific resources that may be valuable for patients who experience health disparities related to housing insecurity.

*Strongly Disagree*

*Disagree*

*Agree*

*Strongly Agree*

6. I can name specific resources that may be valuable for patients who experience health disparities related to financial constraints.

*Strongly Disagree*

*Disagree*

*Agree*

*Strongly Agree*

7. As healthcare providers, we have an obligation to screen patients for social risk and provide resources for patients who experience disparities in health care quality when appropriate.

8. The interdisciplinary team is valuable in the assessment and management of social risk in our intensive care unit patients.

*Strongly Disagree*

*Disagree*

*Agree*

*Strongly Agree*

9. I utilize a standardized screening tool to assess social risk for my patients in more than one social domain.

*Strongly Disagree*

*Disagree*

*Agree*

*Strongly Agree*

10. I routinely include my understanding of a patient’s social risk in the medical plan while the patient is hospitalized.

*Strongly Disagree*

*Disagree*

*Agree*

*Strongly Agree*

11. I receive formal training to assess social risk.

*Strongly Disagree*

*Disagree*

*Agree*

*Strongly Agree*

12. I receive formal feedback on my ability to assess social risk.

*Strongly Disagree*

*Disagree*

*Agree*

*Strongly Agree*

13. I receive evaluations from individuals outside of my discipline of practice.

*Strongly Disagree*

*Disagree*

*Agree*

*Strongly Agree*

14. If you have receiving training to assess social risk OR utilize a standardized screening tool to assess social risk, please describe the training or tool: ______________________________.

**Critical Care and Health Disparities Curriculum**

Resident Post-Rotation Survey

1. So we can compare pre- and post- survey answers, while maintaining your anonymity, please list the two digits for your birth month followed by your last two social. For example if you were born in January and your social ends in 56, enter 0156.

Knowledge:

2. I can identify and describe how social determinants of health affect quality of care for patients experiencing disparities in health care quality.

*Strongly Disagree*

*Disagree*

*Agree*

*Strongly Agree*

3. I can recognize more than one social domain that may contribute to a patient’s social risk.

*Strongly Disagree*

*Disagree*

*Agree*

*Strongly Agree*

4. I can name specific resources that may be valuable for patients who experience health disparities related to substance abuse.

*Strongly Disagree*

*Disagree*

*Agree*

*Strongly Agree*

5. I can name specific resources that may be valuable for patients who experience health disparities related to housing insecurity.

*Strongly Disagree*

*Disagree*

*Agree*

*Strongly Agree*

6. I can name specific resources that may be valuable for patients who experience health disparities related to financial constraints.

*Strongly Disagree*

*Disagree*

*Agree*

*Strongly Agree*

7. As healthcare providers, we have an obligation to screen patients for social risk and provide resources for patients who experience disparities in health care quality when appropriate.

8. The interdisciplinary team is valuable in the assessment and management of social risk in our intensive care unit patients.

*Strongly Disagree*

*Disagree*

*Agree*

*Strongly Agree*

9. I utilize a standardized screening tool to assess social risk for my patients in more than one social domain.

*Strongly Disagree*

*Disagree*

*Agree*

*Strongly Agree*

10. I routinely include my understanding of a patient’s social risk in the medical plan while the patient is hospitalized.

*Strongly Disagree*

*Disagree*

*Agree*

*Strongly Agree*

11. I received formal training to assess social risk.

*Strongly Disagree*

*Disagree*

*Agree*

*Strongly Agree*

12. I received formal feedback on my ability to assess social risk.

*Strongly Disagree*

*Disagree*

*Agree*

*Strongly Agree*

13. I have received evaluations from individuals outside of my discipline of practice.

*Strongly Disagree*

*Disagree*

*Agree*

*Strongly Agree*

14. What component of this curriculum did you find most valuable?

15. What component of this curriculum did you find least valuable?

16. Please indicate any additional topics you would like to learn about with regards to social determinants of health: _____________________________________________.
