# Supplementary material for: A Case-Based Critical Care Curriculum for Internal Medicine Residents Addressing Social Determinants of Health
Source: MedEdPORTAL. 2021 Mar 18;17:11128. doi: 10.15766/mep_2374-8265.11128 (PMC8015637; doi:10.15766/mep_2374-8265.11128)
Supplement: Supplementary file 1 — Needs Assessment.docxFacilitator Guide.docxSDOH Topics Guide.docxCritical Care Cases.docxMDR Checklist.docxPre- and Postcurriculum Surveys.docxCare Team Checklist.docxAttending Checklist.docx [file mep_2374-8265.11128-s001.zip › G. Care Team Checklist.docx]

**MICU Health Disparities Curriculum:** Care Team Survey Checklist

Resident: ____________________________________________

| **Objective** | **Yes** | **Yes with reminder** | **No** | **Notes** |
| --- | --- | --- | --- | --- |
| 1. The resident provided with me with pertinent information for each patient case. |  |  |  |  |
| 2. The resident appropriately utilized my expertise. |  |  |  |  |
| 3. The resident used unbiased and respectful vernacular or language when discussing socially complex patients. |  |  |  |  |

Resident: ____________________________________________

| **Objective** | **Yes** | **Yes with reminder** | **No** | **Notes** |
| --- | --- | --- | --- | --- |
| 1. The resident provided with me with pertinent information for each patient case. |  |  |  |  |
| 2. The resident appropriately utilized my expertise. |  |  |  |  |
| 3. The resident used unbiased and respectful vernacular or language when discussing socially complex patients. |  |  |  |  |

Resident: ____________________________________________

| **Objective** | **Yes** | **Yes with reminder** | **No** | **Notes** |
| --- | --- | --- | --- | --- |
| 1. The resident provided with me with pertinent information for each patient case. |  |  |  |  |
| 2. The resident appropriately utilized my expertise. |  |  |  |  |
| 3. The resident used unbiased and respectful vernacular or language when discussing socially complex patients. |  |  |  |  |
