# Supplementary material for: A Case-Based Critical Care Curriculum for Internal Medicine Residents Addressing Social Determinants of Health
Source: MedEdPORTAL. 2021 Mar 18;17:11128. doi: 10.15766/mep_2374-8265.11128 (PMC8015637; doi:10.15766/mep_2374-8265.11128)
Supplement: Supplementary file 1 — Needs Assessment.docxFacilitator Guide.docxSDOH Topics Guide.docxCritical Care Cases.docxMDR Checklist.docxPre- and Postcurriculum Surveys.docxCare Team Checklist.docxAttending Checklist.docx [file mep_2374-8265.11128-s001.zip › H. Attending Checklist.docx]

**MICU Health Disparities Curriculum:** Attending Survey Checklist

**Resident: _________________________________**

Patient 1:

| **Objective** | **Yes** | **Yes with reminder** | **No** | **Notes** |
| --- | --- | --- | --- | --- |
| 1. The resident appropriately formulates a clinical care plan in the context of the patient’s specific social needs. |  |  |  |  |
| 2. The resident identified the patient’s surrogate appropriately and communicated this effectively with the team. |  |  |  |  |
| 3. The resident appropriately screened and identified social risk within their assessment of the critically ill patient. |  |  |  |  |
| 4. The resident formulated a plan to address one social risk factor for the critically ill patient. |  |  |  |  |
| 5. The resident used unbiased and respectful language in their discussion of the critically ill patient and their socioeconomic challenges. |  |  |  |  |

Patient 2:

| **Objective** | **Yes** | **Yes with reminder** | **No** | **Notes** |
| --- | --- | --- | --- | --- |
| 1. The resident appropriately formulates a clinical care plan in the context of the patient’s specific social needs. |  |  |  |  |
| 2. The resident identified the patient’s surrogate appropriately and communicated this effectively with the team. |  |  |  |  |
| 3. The resident appropriately screened and identified social risk within their assessment of the critically ill patient. |  |  |  |  |
| 4. The resident formulated a plan to address one social risk factor for the critically ill patient. |  |  |  |  |
| 5. The resident used unbiased and respectful language in their discussion of the critically ill patient and their socioeconomic challenges. |  |  |  |  |
